# Supplementary material for: Child and adolescent food insecurity in South Africa: A household-level analysis of hunger
Source: PLoS One. 2022 Dec 28;17(12):e0278191. doi: 10.1371/journal.pone.0278191 (PMC9797094; doi:10.1371/journal.pone.0278191)
Supplement: S1 Fig — (DOCX) [file pone.0278191.s001.docx]

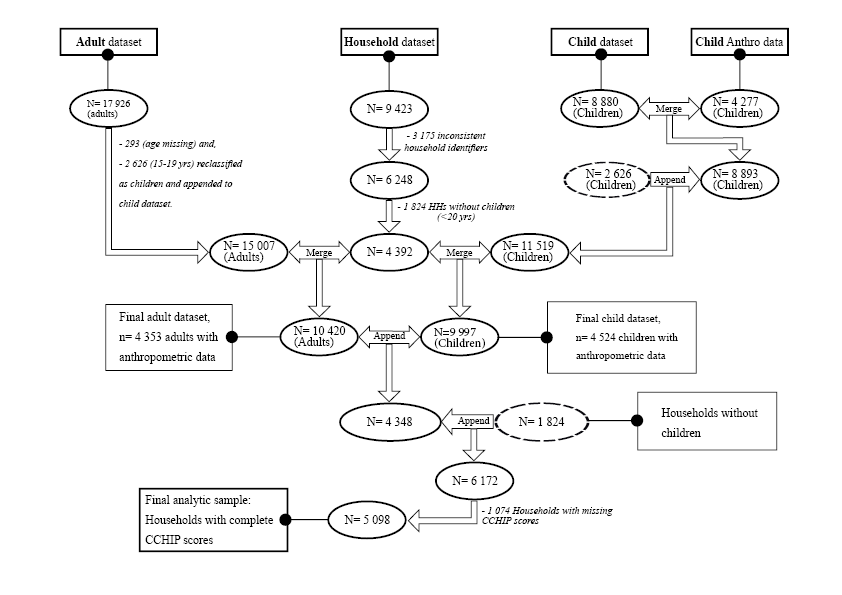
**S1 Fig. Database management, organisation and integration of data extracted from the four household and individual level datasets of the SANHANES-1**

**S1 Fig** illustrates the database management, organisation and integration of the different household and individual-level variables from the five SANHANES-1 questionnaires shared in the four separate databases (‘adult dataset’, “household dataset’, ‘child dataset’ and ‘child anthro dataset’) described earlier. This database management was necessary because the variables of interest were scattered throughout the four different datasets. Furthermore, we needed to reconcile data from adults and children with data contained in the household dataset for CCHIP scores as well as some anthropometric data; and, the “adults” aged 15 – 19 years had to be re-classified as children.

In order to reach the final analytic sample of 5 098 households and their occupants, both adults and children, with complete CCHIP scores, we performed the following steps with the four separate datasets:

1. Firstly, we extracted all persons between the ages of 15 – 19 years from the ‘adult dataset’ to re-classify them as children. This resulted in a decrease in the number of true adults to 15 007, with re-classified children numbering 2 626.
2. Then, we merged the two child datasets (‘child dataset’ and ‘child anthro’), yielding 8 893 children. We then appended the 2 626 re-classified children to the 8 893, yielding a final child database sample of 11 519.
3. From the original ‘household dataset’ of 9 423, we excluded 3 175 households where we could not confirm the inhabitants of the household due to inconsistent unique household identifiers and individual questionnaire numbers. A further 1 824 households without children were set aside (but not excluded) as these households did not require merging with child data. The resulting ‘household dataset’ comprised 4 392 households with children.
4. We then merged the child and adult datasets with this ‘household dataset’ using individual questionnaire numbers and household identifiers to link the individuals within the same household. In this way, we reconciled 10 420 adults and 9 997 children back to their corresponding households (n = 4 348 households with children).
5. At this point, households without children (n= 1 824) were appended to this dataset after which (n= 1 074) households with missing CCHIP scores were dropped, yielding a final analytic sample of 5 098 households with complete CCHIP scores.
