# Supplementary material for: Child and adolescent food insecurity in South Africa: A household-level analysis of hunger
Source: PLoS One. 2022 Dec 28;17(12):e0278191. doi: 10.1371/journal.pone.0278191 (PMC9797094; doi:10.1371/journal.pone.0278191)
Supplement: S1 Table — (DOCX) [file pone.0278191.s002.docx]

**S1 Table.** **Weighted univariable multinomial logistic regression analysis of factors associated with being at risk of hunger and experiencing hunger against being food secure in households with and without children.**

| **Characteristics** | **At risk of hunger** | | **p value** | **Experiencing hunger** | |  |
| --- | --- | --- | --- | --- | --- | --- |
|  | **OR** | **95% CI** |  | **OR** | **95% CI** | **p value** |
| Household size | 0.99 | 0.95 – 1.04 | 0.862 | 1.15 | 1.10 – 1.20 | <0.001 |
| **Children** |  | | | | | |
| Without | ***REF*** | ***REF*** | ***REF*** | ***REF*** | ***REF*** | ***REF*** |
| With | 1.06 | 0.87 – 1.29 | 0.557 | 2.97 | 2.24 – 3.93 | <0.001 |
| **Gender** |  | | | | | |
| Male | ***REF*** | ***REF*** | ***REF*** | ***REF*** | ***REF*** | ***REF*** |
| Female | 1.44 | 1.18 – 1.77 | <0.001 | 2.42 | 1.98 – 2.97 | <0.001 |
| **Age, years** |  | | | | | |
| Age | 1.00 | 0.99 – 1.00 | 0.541 | 1.00 | 1.00 – 1.01 | 0.005 |
| **Race** |  | | | | | |
| African | ***REF*** | ***REF*** | ***REF*** | ***REF*** | ***REF*** | ***REF*** |
| Coloured | 0.49 | 0.36 – 0.65 | <0.001 | 0.23 | 0.15 – 0.33 | <0.001 |
| White/Indian/Asian | 0.24 | 0.11 – 0.53 | <0.001 | 0.05 | 0.01 – 0.15 | <0.001 |
| **Marital status** |  | | | | | |
| Married | ***REF*** | ***REF*** | ***REF*** | ***REF*** | ***REF*** | ***REF*** |
| Living together/civil union | 1.43 | 1.03 – 2.01 | 0.033 | 1.86 | 1.32 – 2.62 | <0.001 |
| Never married | 1.79 | 1.37 – 2.35 | <0.001 | 2.05 | 1.57 – 2.66 | <0.001 |
| Widowed | 1.50 | 1.13 – 1.98 | 0.004 | 2.11 | 1.55 – 2.88 | <0.001 |
| Separated/divorced | 1.63 | 1.06 – 2.52 | 0.025 | 1.83 | 1.12 – 2.98 | 0.015 |
| **Educational attainment** |  | | | | | |
| Primary | ***REF*** | ***REF*** | ***REF*** | ***REF*** | ***REF*** | ***REF*** |
| Secondary | 0.65 | 0.50 – 0.84 | 0.001 | 0.37 | 0.29 – 0.48 | <0.001 |
| Tertiary/Higher degree | 0.18 | 0.12 – 0.29 | <0.001 | 0.04 | 0.02 – 0.08 | <0.001 |
| No schooling/other | 0.73 | 0.52 – 1.01 | 0.064 | 0.79 | 0.60 – 1.04 | 0.098 |
| **Source of income** |  | | | | | |
| Salaries and/or wages | ***REF*** | ***REF*** | ***REF*** | ***REF*** | ***REF*** | ***REF*** |
| Pensions/Grants/Remittances | 1.98 | 1.53 – 2.55 | <0.001 | 3.16 | 2.36 – 4.23 | <0.001 |
| Sale of products and services | 1.36 | 0.83 – 2.21 | 0.210 | 1.16 | 0.58 – 2.31 | 0.670 |
| No income | 2.96 | 2.17 – 4.05 | <0.001 | 3.75 | 2.67 – 5.28 | <0.001 |
| **Locality** |  |  |  |  |  |  |
| Urban formal | ***REF*** | ***REF*** | ***REF*** | ***REF*** | ***REF*** | ***REF*** |
| Urban informal | 2.57 | 1.76 – 3.75 | <0.001 | 3.19 | 2.09 – 4.85 | <0.001 |
| Rural formal | 2.04 | 1.40 – 2.96 | <0.001 | 2.12 | 1.43 – 3.13 | <0.001 |
| Rural informal | 2.00 | 1.48 – 2.68 | <0.001 | 3.58 | 2.60 – 4.92 | <0.001 |
