# Supplementary material for: Child and adolescent food insecurity in South Africa: A household-level analysis of hunger
Source: PLoS One. 2022 Dec 28;17(12):e0278191. doi: 10.1371/journal.pone.0278191 (PMC9797094; doi:10.1371/journal.pone.0278191)
Supplement: Attachment — Submitted filename: PONE-D-20-39991_R1_reviewerEA.pdf [file pone.0278191.s006.pdf]

PLOS ONE

Childhood food insecurity in South Africa: A household-level analysis of hunger

--Manuscript Draft--

Manuscript Number:	PONE-D-20-39991R1
Article Type:	Research Article
Full Title:	Childhood food insecurity in South Africa: A household-level analysis of hunger
Short Title:	Childhood food insecurity
Corresponding Author:	Siluleko Advice Mkhize, MSc Med<br>University of the Witwatersrand<br>Johannesburg, Gauteng SOUTH AFRICA
Keywords:	Keywords: children; household; Food Insecurity; Hunger; CCHIP index; South Africa; SANHANES
Abstract:	Abstract<br><br>Food insecurity impacts childhood nutritional status, physical and cognitive development, and increases lifetime risk for chronic disease. Previous South African studies have examined hunger at sub-national level without a specific focus on children. We therefore determined the national prevalence of childhood food insecurity and identified factors associated with hunger within the household. Individual and household-level data were extracted from the South African National Health and Nutrition Examination Survey (SANHANES-1). Prevalence of food insecurity was assessed using the Community Childhood Hunger Identification Project (CCHIP) index. Multinomial logistic regression analyses were conducted on all households (with and without children) to determine the predictors of food insecurity. Of 5 098 households surveyed, 68.6% had children (0-19 years). Of the households with children, 32.5% [95% CI (29.5 – 35.7)] were experiencing hunger and 26.3% [95% CI (23.9 – 28.8)] were at risk of hunger. Among all the households, significant associations for households experiencing hunger were the presence of children: AOR [95% CI]: 1.68 [1.12 – 2.53]; being female-headed: AOR [95% CI]: 1.53 [1.21 – 1.94] and informally-located; AOR [95% CI]: 1.61 [1.07 – 2.43]. Having a non-African household head, Coloured: AOR [95% CI]: 0.29 [0.19 – 0.44] and White/Indian/Asian: 0.12 [0.04 – 0.33] conferred lower odds of experiencing hunger and, as well as the household head having secondary/tertiary education conferring lower odds of experiencing hunger; AOR [95% CI]: 0.40 [0.28 – 0.56] and being at risk of hunger; AOR [95% CI]: 0.69 [0.52 – 0.92] respectively. Receiving social grants, pensions, or remittances more than doubled the risk of experiencing hunger; AOR [95% CI]: 2.15 [1.49 – 3.09]. In summary, only 41% of South African households with children were food secure. These findings indicate a critical need for multi-sectoral interventions to bolster sustainable food systems for households with children, and to improve public protections for female-headed, African-headed and informally-located households dependent on social grants.
Order of Authors:	Siluleko Advice Mkhize, MSc Med
	Elena Libhaber
	Ronel Sewpaul
	Priscilla Reddy
	Laurel Baldwin-Ragaven
Response to Reviewers:	See attached "Response to Reviewers" file
Additional Information:	
Question	Response
**Financial Disclosure**<br><br>Enter a financial disclosure statement that	The author(s) received no specific funding for this work

Powered by Editorial Manager® and ProduXion Manager® from Aries Systems Corporation

describes the sources of funding for the work included in this submission. Review the submission guidelines for detailed requirements. View published research articles from *PLOS ONE* for specific examples.<br><br>This statement is required for submission and **will appear in the published article** if the submission is accepted. Please make sure it is accurate.<br><br>**Unfunded studies**<br>Enter: *The author(s) received no specific funding for this work.*<br><br>**Funded studies**<br>Enter a statement with the following details:<br>• Initials of the authors who received each award<br>• Grant numbers awarded to each author<br>• The full name of each funder<br>• URL of each funder website<br>• Did the sponsors or funders play any role in the study design, data collection and analysis, decision to publish, or preparation of the manuscript?<br>• **NO** - Include this sentence at the end of your statement: *The funders had no role in study design, data collection and analysis, decision to publish, or preparation of the manuscript.*<br>• **YES** - Specify the role(s) played.<br><br>* typeset	
**Competing Interests**<br><br>Use the instructions below to enter a competing interest statement for this submission. On behalf of all authors, disclose any competing interests that could be perceived to bias this work—acknowledging all financial support and any other relevant financial or non-financial competing interests.<br><br>This statement **will appear in the published article** if the submission is accepted. Please make sure it is accurate. View published research articles from *PLOS ONE* for specific examples.	The authors have declared that no competing interests exist

Powered by Editorial Manager® and ProduXion Manager® from Aries Systems Corporation

**NO authors have competing interests**<br><br>Enter: *The authors have declared that no competing interests exist.*<br><br>**Authors with competing interests**<br><br>Enter competing interest details beginning with this statement:<br><br>*I have read the journal's policy and the authors of this manuscript have the following competing interests: [insert competing interests here]*<br><br>* typeset	
**Ethics Statement**<br><br>Enter an ethics statement for this submission. This statement is required if the study involved:<br><br>• Human participants<br>• Human specimens or tissue<br>• Vertebrate animals or cephalopods<br>• Vertebrate embryos or tissues<br>• Field research<br><br>Write "N/A" if the submission does not require an ethics statement.<br><br>General guidance is provided below. Consult the submission guidelines for detailed instructions. **Make sure that all information entered here is included in the Methods section of the manuscript.**	The present study received ethics approval from the Human Research Ethics Committee (Medical) of the University of the Witwatersrand, Johannesburg, South Africa (Clearance certificate number: M180775).

Powered by Editorial Manager® and ProduXion Manager® from Aries Systems Corporation

**Format for specific study types**<br><br>**Human Subject Research (involving human participants and/or tissue)**<br>• Give the name of the institutional review board or ethics committee that approved the study<br>• Include the approval number and/or a statement indicating approval of this research<br>• Indicate the form of consent obtained (written/oral) or the reason that consent was not obtained (e.g. the data were analyzed anonymously)<br><br>**Animal Research (involving vertebrate animals, embryos or tissues)**<br>• Provide the name of the Institutional Animal Care and Use Committee (IACUC) or other relevant ethics board that reviewed the study protocol, and indicate whether they approved this research or granted a formal waiver of ethical approval<br>• Include an approval number if one was obtained<br>• If the study involved *non-human primates*, add *additional details* about animal welfare and steps taken to ameliorate suffering<br>• If anesthesia, euthanasia, or any kind of animal sacrifice is part of the study, include briefly which substances and/or methods were applied<br><br>**Field Research**<br><br>Include the following details if this study involves the collection of plant, animal, or other materials from a natural setting:<br>• Field permit number<br>• Name of the institution or relevant body that granted permission	
**Data Availability**<br><br>Authors are required to make all data underlying the findings described fully available, without restriction, and from the time of publication. PLOS allows rare exceptions to address legal and ethical concerns. See the PLOS Data Policy and FAQ for detailed information.	Yes - all data are fully available without restriction

Powered by Editorial Manager® and ProduXion Manager® from Aries Systems Corporation

A Data Availability Statement describing where the data can be found is required at submission. Your answers to this question constitute the Data Availability Statement and **will be published in the article**, if accepted.<br><br>**Important:** Stating 'data available on request from the author' is not sufficient. If your data are only available upon request, select 'No' for the first question and explain your exceptional situation in the text box.<br><br>Do the authors confirm that all data underlying the findings described in their manuscript are fully available without restriction?	
**Describe where the data may be found in full sentences. If you are copying our sample text, replace any instances of XXX with the appropriate details.**<br><br>• If the data are **held or will be held in a public repository**, include URLs, accession numbers or DOIs. If this information will only be available after acceptance, indicate this by ticking the box below. For example: *All XXX files are available from the XXX database (accession number(s) XXX, XXX.*).<br>• If the data are all contained **within the manuscript and/or Supporting Information files**, enter the following: *All relevant data are within the manuscript and its Supporting Information files.*<br>• If neither of these applies but you are able to provide **details of access elsewhere**, with or without limitations, please do so. For example:<br><br>*Data cannot be shared publicly because of [XXX]. Data are available from the XXX Institutional Data Access / Ethics Committee (contact via XXX) for researchers who meet the criteria for access to confidential data.*<br><br>*The data underlying the results presented in the study are available from (include the name of the third party*	See response to reviewers

and contact information or URL).<br>• This text is appropriate if the data are owned by a third party and authors do not have permission to share the data.<br><br>* typeset	
Additional data availability information:	

Powered by Editorial Manager® and ProduXion Manager® from Aries Systems Corporation

Manuscript (Revised clean copy)

Click here to access/download;Manuscript;Manuscript(revised 25 June 2021).docx

Childhood food insecurity in South Africa:

A household-level analysis of hunger

Siluleko A. Mkhize[1], Elena Libhaber[2], Ronel Sewpaul[3], Priscilla Reddy[3,4] and Laurel Baldwin-Ragaven[1*]

[1]Department of Family Medicine and Primary Care, School of Clinical Medicine, Faculty of Health Sciences, University of the Witwatersrand, Johannesburg, South Africa

[2]School of Clinical Medicine and Health Sciences Research Office, Faculty of Health Sciences, University of the Witwatersrand, Johannesburg, South Africa

[3]Health and Wellbeing, Human and Social Capabilities Division, Human Sciences Research Council, Cape Town, South Africa

[4]Faculty of Health Sciences, Nelson Mandela University, Port Elizabeth, South Africa

*****Corresponding author

E-mail: laurel.baldwin-ragaven@wits.ac.za (LBR)

Contributions

SM, LBR and EL conceptualised the study and its design. SM, EL, LBR and RS conducted and interpreted the analyses. SM wrote the first draft of the manuscript; and, EL LBR and RS worked on subsequent iterations of the manuscript. RS and PR critically revised the manuscript for important intellectual content. All authors approved the final manuscript and take primary responsibility for its contents. The contents of the manuscript are solely the responsibility of the

authors and do not necessarily represent the official views of the HSRC or the University of the Witwatersrand, Johannesburg, South Africa.

ORCID

Siluleko Mkhize https://orcid.org/ 0000-0002-8351-6891

Elena Libhaber https://orcid.org/ 0000-0002-7043-4002

Laurel Baldwin-Ragaven https://orcid.org/ 0000-0002-6744-3768

Ronel Sewpaul https://orcid.org/ 0000-0002-2523-1222

Priscilla Reddy https://orcid.org/ 0000-0002-0432-9060

Abstract

Food insecurity impacts childhood nutritional status, physical and cognitive development, and increases lifetime risk for chronic disease. Previous South African studies have examined hunger at the sub-national level without a specific focus on children. We, therefore, determined the national prevalence of childhood food insecurity and identified factors associated with hunger within the household. Individual and household-level data were extracted from the South African National Health and Nutrition Examination Survey (SANHANES-1). Prevalence of food insecurity was assessed using the Community Childhood Hunger Identification Project (CCHIP) index. Multinomial logistic regression analyses were conducted on all households (with and without children) to determine the predictors of food insecurity. Of 5 098 households surveyed, 68.6% had children (0-19 years). Of the households with children, 32.5% [95% CI (29.5 – 35.7)] were experiencing hunger and 26.3% [95% CI (23.9 – 28.8)] were at risk of hunger. Among all the households, significant associations for households experiencing hunger, were the presence of children: AOR [95% CI]: 1.68 [1.12 – 2.53]; being female-headed: AOR [95% CI]: 1.53 [1.21 – 1.94] and informally-located; AOR [95% CI]: 1.61 [1.07 – 2.43]. Having a non-African household head, coloured of the racial groups: AOR [95% CI]: 0.29 [0.19 – 0.44] and White/Indian/Asian: 0.12 [0.04 – 0.33] conferred lower odds of experiencing hunger and, as well as the household head having secondary/tertiary education conferring lower odds of experiencing hunger; AOR [95% CI]: 0.40 [0.28 – 0.56] and being at risk of hunger; AOR [95% CI]: 0.69 [0.52 – 0.92] respectively. Receiving social grants, pensions, or remittances more than doubled the risk of experiencing hunger; AOR [95% CI]: 2.15 [1.49 – 3.09]. In summary, only 41% of South African households with children were food secure. These findings indicate a critical need for multi-sectoral interventions to bolster sustainable food systems for households with children and to improve

Formatted: Right: 0"

Deleted: therefore

Deleted: C

Deleted: ,

public protections for female-headed, African-headed and informally-located households dependent on social grants.

Keywords: children, household, food insecurity, hunger, CCHIP index, South Africa, SANHANES

Introduction

Food insecurity has emerged as a major public health concern; and, the obligation to realise the "*right to food and basic nutrition"* is enshrined in both the United Nations Charter [1] and the South African constitution [2]. The Food and Agriculture Organization (FAO) of the United Nations states that "food security exists when all people, at all times, have physical, social and economic access to sufficient, safe and nutritious food that meets their dietary needs and food preferences for an active and healthy life" [3], emphasising that the availability of food must be beyond mere subsistence [4]. In Sub-Saharan Africa, one in four people were undernourished in 2017 which represents about one-third of the 821 million people suffering from chronic hunger globally [5]. According to actuarial projections, Africa will be home to 90% of the world's poorest by 2030 [6].

Deleted: charter

Deleted: ,

Deleted: one

In 2017, 6.8 million South Africans were affected by hunger [7]. Although this number has halved from 13.5 million in 2002, about 1.7 million households across the country remain food insecure [7]. Moreover, a considerable but undocumented proportion of these households comprise children and adolescents who are vulnerable to food deprivation and hunger [4]. Indeed, the presence of children in households may confer additional "*pressure of mouths to feed*" [8,9].

Throughout the world, household food insecurity is associated with malnutrition, stunting and wasting among children [10]. The largest national census of South African households, the South African National Health and Nutrition Examination Survey (SANHANES), reported in 2011/12 that 23.5% of children aged 0-14 years were malnourished (stunted, wasted, or undernourished) [11]. In 2019, Sung-King, et al. [12], using a different methodology, found that ~48.8% of children under five years were malnourished. Nationally, severe-acute and moderate-

acute malnutrition accounts for between 4% to 25% of child mortality [13]. Hunger has profound health consequences that not only affect physical growth and cognitive development in childhood [14,15], but endure throughout the life-course, into adulthood [16,17] and extend into subsequent generations [18,19]. Two longitudinal cohort studies of the consequences of hunger that began in the throes of the Second World War, the Dutch Winter Famine [19] and the siege of Leningrad [18,20] indicate that the effects of intra-uterine exposure to food insecurity can traverse through multiple generations, predisposing to a "thrifty phenotype" among progeny. Bjerregaard, et al. [21] found that another form of malnutrition, obesity in childhood (measured at ages 7 and 13 years), significantly increased the hazards of developing type-2 diabetes in late adulthood among Danish men. Conversely, reversal of obesity in adolescence reduced the risk by 4%, a finding that may be explained, in part, by food insecurity given that obesity and type-2 diabetes are primarily diet-sensitive adverse health outcomes [15].

Deleted: moreover

Deleted: second

Deleted: ,

In South Africa, three national surveys conducted between 1999 and 2008 estimated the prevalence of food insecurity using the Community Childhood Hunger Identification Project (CCHIP) index: the National Food Consumption Surveys of 1999 and 2005, and the South African Social Attitudes Survey of 2008. A review of these studies, which included children from 1-9 years old with at least one adult in the household, indicated a marked decline of household food insecurity from 52.3% in 1999 to 25.9% in 2008 [22]. These studies looked at a segment of children (those 1-9 years old), thus perhaps underestimating the true extent of childhood food insecurity [23,24]. Much of what is known about the sociodemographic correlates of food insecurity in South Africa has been gleaned from sub-national cross-sectional studies. Food insecurity measured using the Household Food Insecurity Access Scale (HFIAS) [25] indicated a gradient across the rural-

urban continuum of South African towns, with households in rural areas reporting higher scores, and therefore more food insecurity, than peri-urban households (households located in urban-informal settlements) [25]. Households that are reliant on private food charities and government assisted social grants are at an increased risk of food insecurity [26]. It is speculated that while social grants are regular and predictable, they are, however, not sensitive to the changing needs of a household [26,27]. The gender of the household head is also an important factor predicting household vulnerabilities, with the highest prevalence of food insecurity in South Africa, reported among female-headed households [9,13].

There is a paucity of nationally representative data to assess the granular detail about food insecurity prevalence across the country. The critical need to address hunger in childhood necessitates particular attention to factors affecting food insecurity in households with children and adolescents, so that nutrition-sensitive interventions can be developed and/or strengthened [28]. Such interventions would address the underlying determinants of child hunger and include, for example, multi-sectoral approaches to social safety netting through minimum guaranteed income and/or cash transfers; financial, educational and parenting support to female-headed households with children; and bolstering health services and sustainable agricultural practices [5,29]. Such multi-pronged approaches are necessary to mediate the short-term, long-term and inter-generational adverse health consequences of food insecurity on childhood health. Therefore, drawing from a large-scale population-based sample of households, the SANHANES-1, we set out to determine the prevalence of food insecurity in households with and without children as well as to assess associations between the sociodemographic characteristics of the household head and degrees of hunger.

Commented [EA1]: Any reason found in the literature for this. It will be nice if you add few reasons as being found in the literature. If there is none, it may be stated.

Deleted: ,

Deleted: ,

Methods

Study design

We conducted a secondary data analysis of the SANHANES-1 [11]. The SANHANES-1 is a cross-sectional nationally representative sample of households that employed a multi-stage disproportionate, stratified cluster sampling approach based on the 2001 census Enumeration Areas (EAs). The first 20 households were sampled from each of the 500 EAs, yielding a sample of 10 000 households. Ensuring national representativeness, the SANHANES-1 EA sampling was layered by type of locality (urban/rural; formal/informal), and province (there are nine in South Africa) as well as by "race" in formal urban areas. The race allows investigation of ongoing health disparities that have endured post-apartheid. In this way, the SANHANES captures the sociodemographic and economic profile of the country. Additional sample selection details have been published elsewhere [11]. In our study, we used the "household" as the unit of analysis. In South Africa, a household is defined as "a group of people living in the same dwelling and sharing food from the same cooking pot" [7]. In the SANHANES-1, household membership was attributed to persons who occupied the same dwelling and slept in the household for at least four nights a week. The household head was identified as a physically present member who was designated internally as the head of that household, and also served as the main respondent for household-level data acquisition.

Deleted: Race

Data extraction and study population

The SANHANES-1 database includes people of all ages residing in households in South Africa. For the present study, only occupied households were included **(S1 Fig)**. Five separate but inter-related SANHANES-1 questionnaires were administered at the household level by a trained field worker capturing information on everyone living in that household (between 1 and 20+ people). The variables of interest for this study were extracted from each of the five questionnaires: visiting point (household) questionnaire (administered to the household head); child questionnaire (0-14 years old); child clinical examination form (0-14 years old); adult questionnaire (administered to anyone ≥ 15 years old); and the adult clinical examination form (≥ 15 years old). After extraction, these data were entered into four separate newly created databases. Children, whom we defined as aged 0-19 years old, and adults aged ≥20 years old were extracted from both the child (<15 years) and adult (≥15 years) individual-level datasets, respectively. This age selection was made to align with global initiatives that target child and adolescent public health [30,31] as well as in keeping with the World Health Organization multicentre growth reference standards for assessing anthropometry in children and adolescents [32]. After merging data for individuals aged 15-19 with data from those 0-14 years old, thereby reclassifying everyone aged 0-19 years old as children, we then reconciled them back into their corresponding households. The final sample that we analysed included 5 098 households and their occupants, both adults and children. **S1 Fig** illustrates the database management, organisation and integration of the different household and individual-level variables from the five SANHANES-1 databases.

Deleted: -

Deleted: ,

Deleted: ,

Deleted: done

Ethical considerations

The present study received ethics approval from the Human Research Ethics Committee (HREC) (Medical) of the University of the Witwatersrand, Johannesburg, South Africa (Clearance certificate number: M180775).

Variables

Household composition and sociodemographic parameters

At the household level, the parameters included were household size, the number of adults and the number of children occupying a household. Age, self-reported sex (male and female), self-reported race (African, Coloured, White/Indian/Asian), educational attainment (no schooling, primary, secondary, tertiary/higher degrees), marital status (married, living together/civil union, never married, widowed, separated/divorced) and the primary source of income (salaries/wages, social grants/remittances/pensions, sale of products and services, no income) of all household heads were also included. Locality (urban, rural, formal and informal) was pre-assigned to the household from the EA sampling.

Household food insecurity

Food insecurity was measured using the CCHIP index, a validated tool for the assessment of food insecurity at the household level [33]. While five of the eight questions are child-referenced, making the CCHIP a specific tool for measuring *childhood* food insecurity, the CCHIP was also administered to households without children, generalising the child-referenced questions to everyone in the household. A score of ≥5 affirmative responses indicate the presence of hunger, understood as "*experiencing hunger*" and recorded as such. A score of 1-4 affirmative responses

Commented [EA2]: Was the ethical clearance obtained for the use of the secondary data or this was the ethical clearance obtained for the collection of the primary data. If so, please re-phrase to indicate that.

Commented [EA3]: Household-level or Household level. Please ccheck the netire manuscript and be consistent with one.

Deleted: -

Deleted: main

indicates that the household is "*at risk of hunger*". Lastly, a score of zero indicates that the household is "*food secure*".

Statistical analyses

STATA software, version 16.1 (STATA Institute Inc., College Station, TX, USA), was used for database management and statistical analyses. Results are presented as frequencies and percentages for categorical variables and Median [IQR] or Mean ± SD in the case of continuous variables. Comparisons between households with and without children were performed using Mann-Whitney U tests for continuous non-normally distributed data. Categorical variables were compared using Chi-Square tests. All analyses were weighted to offset for the over-allocation of EAs in areas where Indian, Coloured or White racial groups prevailed, to ensure that the minimum required sample size in those minority groups were obtained. All analyses were performed using *svy* function in STATA, incorporating sample weights and stratified cluster sampling design to provide estimates. Weighted prevalence of food security in households with children, stratified by sociodemographic characteristics of the household heads was ascertained from frequencies and percentages; however, age differences in prevalence estimates were computed from a Kruskal-Wallis test with a Bonferroni *post hoc* correction to adjust for multiple comparisons. To improve statistical power in the regression models, locality was dichotomised to formal vs informal; educational attainment (primary, secondary/tertiary/higher degrees and no schooling); marital status (married, never married, living together/civil unions, and widowed/separated/divorced). Weighted univariable and multivariable multinomial logistic regression analyses with robust standard errors were carried out to determine factors associated with being "at risk of hunger" and "experiencing hunger", where the "food secure" category was used as the referent group for

Commented [EA4]: Is it arithmetic or geometric mean? Please indicate?

Deleted: unions

comparison in all households, and separately in households with and without children. Statistical significance was set at $p<0.05$. Multivariable multinomial logistic regression for "risk of hunger" and "experiencing hunger" using adjusted odds ratios with their 95% confidence intervals as measures of association are presented as forest plots, which were produced using GraphPad Prism, version 7.00 for Windows (GraphPad Software, La Jolla, California USA).

Results

Household composition, living arrangements and main source of income of household heads

Table 1 shows the characteristics of South African households with and without children, and the living arrangements of the heads of households. Of the 5 098 households with complete CCHIP scores, 3 499 (68.6%) households contained children aged 0-19 years. Households with children were occupied by 9 505 adults and 9 184 children, while those without children were occupied by 3 243 adults. Households with children were 2.5 times larger than those without children ($p<0.001$). Forty percent of all households in South Africa contained children and were female headed. Of the households containing children, nine were child-headed. When comparing households with and without children, the proportions of African- and Coloured-headed households were not different ($p=0.417$) and, no differences between urban formal and urban informal settings were found following two by two comparisons ($p=0.361$). Furthermore, there was no statistically significant difference in the main source of income for households with and without children ($p=0.381$).

Table 1. Characteristics of occupied South African households with and without children, and sociodemographic characteristics of household heads.

	All Households N=5 098		p value
	With children n=3 499	Without children n=1 599	
**Household characteristics**			
Household size (n=21 932)	5 [4 – 6]	2 [1 – 2]	<0.001
No. of Adults, ≥20 years (n=12 748)	2 [2 – 3]	2 [1 – 2]	<0.001
No. of Children, <20 years (n=9 183)	2 [1 – 3]	-	-
**Gender (n=5 092)**			
Male	1 532 (31.77)	899 (15.76)	<0.001
Female	1 963 (40.44)	698 (12.02)	
**Age, years (n=5 093)**			
Age	49.22 ± 14.67	52.07 ± 15.91	<0.001
**Race (n=5 090) †**			
African	2 465 (60.88) [a]	849 (19.86)	
Coloured	659 (6.86) [b]	263 (2.48)	<0.001
White/Indian/Asian	373 (4.54) [ab]	481 (5.39)	
**Locality (n=4 889) †**			
Urban formal	1 838 (39.25) [ab]	892 (16.15)	
Urban informal	464 (7.1) [cd]	155 (2.54)	<0.001
Rural formal	359 (4.16) [ace]	222 (6.3)	
Rural informal	838 (22.82) [bde]	121 (1.68)	
**Marital status (n=4 998) †**			
Married	1 678 (34.68) [a]	687 (11.15)	
Living together/civil union	310 (6.1) [bc]	73 (1.28)	
Never married	757 (16.02) [abd]	486 (9.5)	<0.001
Widowed	522 (11.38) [d]	236 (4.07)	
Separated/divorced	156 (3.9) [c]	93 (1.93)	
**Source of income (n=4 608)**			
Salaries or wages	1 330 (29.52)	591 (11.00)	
Social grants/pensions/remittances	1 006 (24.7)	498 (9.49)	0.381
Products and services	138 (3.33)	78 (1.71)	
No income	644 (13.98)	323 (6.27)	

Data presented as median [IQR]; mean ± SD and n (%): unweighted (n) and weighted (%).

†Multiple 2x2 comparisons with Bonferroni correction: p<0.05 between households with and without children if categories share the same letter. Race and locality: p<0.001 for both (a) and (b) comparisons, Marital status: p=0.001 in married vs never married and living together vs never married whereas p=0.036 in Separated/divorced vs living together/civil unions and lastly p=0.013 in never married vs widowed.

Commented [EA5]: Indicate the type of mean(arithmetic or geometric)

Prevalence of food insecurity in households with children

In households with children, the prevalence of *experiencing hunger* and being *at risk of hunger* was 32.5% [95% CI (29.5 – 35.7)] and 26.3% [95% CI (23.9 – 28.8)], respectively (**Fig 1**). **Table 2** shows the weighted prevalence of food insecurity stratified by the household head sociodemographic characteristics. Households in which the household head was female, older, of African race, unmarried, not having a formal education, dependent on social grants/pensions/remittances and having no income showed a higher prevalence of experiencing hunger or being at risk of hunger (p<0.001). Further analysis of the collapsed locality categories (formal vs informal) yielded the following: 37.2% of households with children were informally located; and, of these, only 28.9% were food secure. In contrast, of the 62.9% of households with children located in formal areas, 53.6% were food secure (p<0.001).

Deleted: A f

Fig 1. National prevalence of food insecurity in households with children.

Table 2. Weighted prevalence of household food security, stratified by sociodemographic characteristics of designated household heads of South African households with children.

	Total N	Food secure n (%)	†At risk of hunger n (%)	‡Experiencing hunger n (%)	p value
**Gender (n=3 495)**					
Male	1 532	813 (22.16)	356 (11.09) a	363 (10.75) a	<0.001
Female	1 963	739 (19.07)	502 (15.13) a	722 (21.8) a	
**Age, years (n=3 499)**					
Age (Mean±SD)	3 499	47.87±13.85 *	49.76±15.15	50.70±15.25	0.001
**Race (n=3 497)**					
African	2 465	845 (30.18)	662 (23.16) ab	958 (30.89) ab	
Coloured	659	388 (5.85)	155 (22.6) a	116 (1.38) ac	0.001
White/Indian/Asian	373	319 (5.16)	43 (0.85) b	11 (0.27) bc	
**Marital status (n=3 423)**					
Married	1 678	899 (23.43)	375 (12.3) a	404 (12.39) abc	
Living together/civil union	310	112 (3.16)	80 (22.9)	118 (3.01) a	
Never married	757	256 (6.85)	212 (6.25) a	289 (9.13) b	0.001
Widowed	522	196 (5.69)	134 (3.98)	192 (6.12) c	
Separated/divorced	156	61 (1.96)	44 (1.38)	51 (2.07)	
**Educational attainment (n=3 499)**					
Primary	820	243 (6.48)	223 (6.28) a	354 (10.34) ab	
Secondary	1 524	762 (18.27)	390 (12.11) b	372 (11.49) acd	
Tertiary/higher degree	353	280 (8.4)	50 (1.81) abc	23 (0.81) bce	<0.001
No schooling/other	802	268 (8.05)	197 (6.05) c	337 (9.91) de	
**Source of income (n=3 118)**					
Salaries or wages	1 330	774 (22.93)	285 (9.32) ab	271 (9.01) ab	
Social grants/pensions/remittances	1 006	329 (10.69)	266 (9.34) a	411 (14.5) a	
Sale of products and services	138	71 (2.11)	37 (1.46)	30 (1.08) c	<0.001
No income	644	208 (5.54)	174 (5.86) b	262 (8.15) bc	
**Locality (n=3 499)**					
Urban formal	1 838	1 049 (27.85)	410 (12.9) ab	379 (12.77) abc	
Urban informal	464	131 (2.59)	140 (3.11) a	193 (3.98) a	<0.001
Rural formal	359	128 (2.18)	80 (1.19)	151 (2.3) b	
Rural informal	838	245 (8.58)	230 (9.05) b	363 (13.49) c	

Multiple 2x2 comparisons with Bonferroni correction: a) p<0.01 between food secure vs. experiencing hunger except for: ‡ Primary vs no schooling, salaries vs products/services; pensions/grants vs product/services and no income; b) p<0.01 between food secure vs. at risk of hunger except for: †Coloured vs White/Indian/Asian; primary vs secondary; primary vs no schooling and secondary vs no schooling; salary vs product/services, pension/grants vs product/services, pension/grants vs no income and product /services vs no income; c) p<0.01 between food secure and at risk of hunger and experiencing hunger for married vs never married and married vs widower; p<0.01 between food secure vs experiencing hunger for married vs living together. *p<0.001 for age when comparing food secure vs at risk of hunger and food secure vs experiencing hunger (Kruskal-Wallis with Bonferroni correction).

Factors associated with experiencing hunger and being at risk of hunger in all households with and without children

Weighted univariable multinomial logistic regression (**S1 Table**) of all the households exhibits that household heads who were female, of older age, unmarried/widowed/separated vs married were at higher risk of hunger and of experiencing hunger. However, when performing a weighted multivariable multinomial logistic regression (**Figs 2A and 2B**), age was no longer associated with experiencing hunger. Yet, female-headed households had ~1.53 times increased odds of experiencing hunger as compared to male-headed households ($p<0.001$). Having a larger household, households with children present, and having a female household head were predictors of experiencing hunger; AOR [95% CI]: 1.06 [1.00 – 1.21], 1.68 [1.12 – 2.53] and 1.53 [1.21 – 1.94], respectively. Having a household head who was either Coloured or White/Indian/Asian as opposed to African conferred lower odds of both experiencing hunger and being at risk of hunger; AOR [95% CI]: 0.29 [0.19 – 0.44] and 0.12 [0.04 – 0.33] for experiencing hunger, respectively; and, being at risk of hunger; AOR [95% CI]: 0.54 [0.39 – 0.75], 0.22 [0.12 – 0.40], respectively. Furthermore, secondary/tertiary education conferred lower odds of both experiencing hunger; AOR [95% CI]: 0.40 [0.28 – 0.56] and being at risk of hunger; AOR [95% CI]: 0.69 [0.52 – 0.92]. Households relying on pensions/social grants/remittances, or not having any source of income, were ~1.76 to 2.15 times more at risk of hunger and experiencing hunger than those receiving a salary/wage.

Fig 2A. Factors associated with being at risk of hunger, data presented as adjusted odds ratios (AOR) and 95% Confidence intervals (95% CI).

Reference categories: *Presence of children (no children present), Gender (male), Race (African), Marital status (married), Educational attainment (primary), Source of income (salaries/wages), Locality (formal)*

Fig 2B. Factors associated with experiencing hunger, data presented as adjusted odds ratios (AOR) and 95% Confidence intervals (95% CI).

Reference categories: *Presence of children (no children present), Gender (male), Race (African), Marital status (married), Educational attainment (primary), Source of income (salaries/wages), Locality (formal)*

Sensitivity analysis

S2 and S3 Tables show the weighted multivariable multinomial logistic regression analysis of factors associated with being at risk of hunger and experiencing hunger against being food secure when the analysis was restricted to households with and without children. In *households without children* (**S2 Table**), the non-African race, i.e. Coloured AOR [95% CI]: 0.44 [0.27 – 0.73] and White/Indian/Asian 0.28 [0.15 – 0.53] race groups and, secondary/tertiary/higher degrees 0.52 [0.30 – 0.88] had lower odds of being at risk of hunger. No income 3.78 [2.01 – 7.08] was not associated with being at risk of hunger. The female sex AOR [95% CI]: 2.00 [1.15 – 3.50] and no income 5.16 [2.25 – 11.82] conferred increased odds of experiencing hunger while the Coloured 0.15 [0.06 – 0.34] and White/Indian/Asian 0.12 [0.03 – 0.38] race groups and households with heads who attained secondary/tertiary/higher degrees 0.31 [0.14 – 0.70] had lower odds of experiencing hunger.

In *households with children* (**S3 Table)**, only the non-African race groups had lower odds of being at risk of hunger, Coloured AOR [95% CI]: 0.64 [0.45 – 0.92] and White/Indian/Asian 0.16 [0.07 – 0.33]. Whereas, pensions/social grants/remittances 1.69 [1.18 – 2.42], no income 2.08 [1.41 – 3.07] and informal location 1.64 [1.20 – 2.24] increased the odds of being at risk of hunger. Again, the non-African race groups, Coloured 0.36 [0.24 – 0.55] and White/Indian/Asian 0.11 [0.03 – 0.36] had lower odds of experiencing hunger. To summarise, in households with children,

Deleted: ,

factors that increased the odds of experiencing hunger were household size AOR [95% CI]: 1.06 [1.00 – 1.12], female sex 1.45 [1.07 – 1.95], living together/civil unions 1.72 [1.11 – 2.68], never married 1.46 [1.02 – 2.09], relying on social grants/pensions/remittances and sale of products and services 1.92 [1.31 – 2.79], having no income 2.83 [1.89 – 4.24] and residing in informal settings 1.78 [1.27 – 2.49].

We further illustrate the association due to the presence of children in the household with food security, adjusting for the number of adults in households and the same sociodemographic factors, including the main source of income, as in Figs 2A and 2B (**Table 3**). In the univariate analysis, the presence of children confers nearly three times the odds of experiencing hunger OR [95% CI]: 2.97 [2.24 – 3.93]. After adjustment for the number of adults in a household and the sociodemographic characteristics of the household head, including source of income, the presence of children remained independently associated with experiencing hunger AOR [95% CI]: 2.59 [1.78 – 3.77], but not with being at risk of hunger (**Table 3**).

Deleted: ,

Table 3. Unadjusted and adjusted weighted multinomial logistic regression analyses between the presence of children in a household and being at risk of hunger or experiencing hunger, against food security.

	**Model 1**<br><br>**Odds Ratio [95% CI] Unadjusted**	**p-value**	**Model 2**<br><br>**Adjusted Odds Ratio [95% CI] for the number of adults**	**p-value**	**Model 3**<br><br>**Adjusted Odds Ratio [95% CI] for number of adults + sociodemographic characteristics of household head[1]**	**p-value**
**Food security status**						
Food secure	***REF***		***REF***		***REF***	
At risk of hunger	1.06 [0.87 – 1.29]	0.557	1.11 [0.90 – 1.36]	0.303	0.88 [0.69 – 1.12]	0.330
Experience hunger	**2.97 [2.24 – 3.93]**	<0.001	**3.07 [2.29 – 4.11]**	<0.001	**2.59 [1.78 – 3.77]**	<0.001

[1] Variables included: sex, race, marital status, education, location and source of income.

Discussion

To the best of our knowledge, the present study is the first to objectively quantify the burden of food insecurity among children and adolescents in South Africa and draw associations using a large-scale, population-based sample of households with children. No studies in South Africa have disaggregated households with and without children thus possibly underestimating the true extent of childhood food insecurity and limiting direct comparisons to the present study. Indeed, there is an observed increase in food insecurity (experiencing hunger—25.9% to 32.5%) by 6.6% between the prevalence estimate reported for 2008 by Labadarios et al. [22] and the estimate generated in the present study. This lack of improvement may be accounted for by the enduring socio-political and economic challenges sustained since the end of apartheid [22,34]. Drawing from a cross-sectional, population-based national sample of South African households, we have shown that the prevalence of food insecurity in households with children was nearly 60%. This is similar to the original SANHANES study where food insecurity among all households was 54% [11]. In the present study, after adjusting for the number of adults in a household, the sociodemographic characteristics of the household head and the main source of income, the presence of children conferred ~2.59 increased odds of food insecurity (experiencing hunger) compared to households without children. Having a female vs male household head, having an African household head compared to all other race groups, living in informal settings vs formal settings and having no income compared to relying on salaries or wages increased the household risk of experiencing hunger between 53% and 300%. Secondary/tertiary/higher educational attainment of the household head reduced the risk of experiencing hunger by at least 40% relative

to primary education. However, having a household head with no formal schooling was not a predictor for experiencing hunger. In addition, the marital status of the household head was not independently associated with being at risk of hunger but living together/civil unions and never married increased the odds of experiencing hunger by 61% and 41%, respectively. Relying on social grants, pensions, and remittances as the main source of income were not protective against food insecurity.

Deleted: pensions

Deleted: was

Gender, presence of children, racial differences, rurality, and informality

The majority of households with children in our sample were headed by women, had African household heads, or were living in urban informal or rural informal areas; therefore, the increased vulnerability of households with children may be explained, in part, by the additive effects of these known predictors of food insecurity [35]. A mounting body of evidence has corroborated that the presence of children in households is independently associated with food insecurity, beyond sociodemographic characteristics and measures of socioeconomic status [36–39]. Our findings also reveal that the presence of children confers 2.5 times increased odds of experiencing hunger beyond sociodemographic characteristics of the household head, including the head's main source of income and educational attainment. Additional research has demonstrated that the sex of the household head is an important factor predicting household vulnerabilities, with the highest prevalence of hunger reported among female-headed households [7,9,13,26], a risk that worsens with rural location [25,40]. In contrast to a study by Chakona and Shackleton [25], in which food insecurity increased along the urban-rural continuum of South African towns selected along differences and becomes more pronounced when stratifying by

Commented [EA6]: Not too clear, please rephrase

formal vs informal type of household location. In their large-scale, population-based longitudinal analysis of the Indonesian Family Life Survey, Vaezghasemi et al. [41] further argued that while women, who are at the bottom of the family hierarchy with low social capital, may be conscious of healthy food options but are nonetheless defenseless against household hunger. Given South Africa's colonial and apartheid history, racial differences persist in the prevalence of food insecurity, with black or African households bearing the greatest burden compared to minority groups [7,12,13,26].

Deleted: still

Main source of household income and social grants

Using a self-reported primary source of income to indicate a household's economic stability, we note that there were no differences in income source between households with and without children. We show that not having any source of income predicts that a household will experience a three-fold increase in experiencing hunger. Furthermore, similar to Ruiters and Wildschutt [27], receiving government social grants and pensions, or remittances did not alleviate hunger nor the risk of being food insecure for households with and without children. Social grants were not protective in these households, with a 76% increase in being at risk of food insecurity and double the odds for being food insecure. Furthermore, even when controlling for the sociodemographic characteristics of the household head and the main source of income, the risk of experiencing hunger was increased with the presence of children in a household. Other contexts show mixed results for government assistance programmes on food security. Tarasuk, Fafard St-Germain and Mitchell [42] have shown that in Canada, household before-tax income adjusted for family size was protective against food insecurity; however, households receiving social assistance were three times more likely to be food insecure. In contrast, Brown and Tarasuk [43] reported

Deleted: main

Deleted: ,

Deleted: In fact, s

reductions in severe food insecurity among households with children subsequent to the roll-out of the Canada Child Benefit (CCB), a country-wide non-means-tested cash transfer programme. In Brazil, participation in the Bolsa Familia Programme [44] and in the United States receiving Supplemental Nutrition Assistance Program (SNAP, formerly the Food Stamp Program) support [8,45], were not effective in preventing food insecurity. In one South African study where social grants did show improvements in household food insecurity and dietary quality, the authors [26] note that these findings were not accompanied by concomitant improvements in anthropometric indicators of child malnutrition. Furthermore, while they observed that the Older Persons Grant and remittances had substantial and favorable effects on the dietary diversity score, the Child Support Grant did not [26]. Our findings did not support a similar benefit in households without children. Moreover, when children were present, reliance on social grants, pensions or remittances as the main source of income, was not protective against food insecurity.

Educational attainment

` Previous studies have demonstrated strong positive associations between the lowest levels of educational attainment and food insecurity, mitigated by higher levels of educational attainment [5,46–49]. Likewise, we found that improved levels of education (secondary and tertiary educational attainment) reduced the risk of food insecurity by at least 40%; yet, having no formal schooling did not predict food insecurity. As Chakona and Shackleton [25] also found, we posit that household heads without a formal education may rely on subsistence farming or implement other skills-based practices which act as a safety net against food insecurity, thereby insulating the adverse effects of any transitory financial shocks on household food expenditure.

Marital status

The Born in Bradford cohort, a longitudinal study, showed associations between cohabitation status and food insecurity, where pregnant women not living with a partner had a two-fold increased risk of food insecurity [50]. Hanson, Sobal, and Frongillo [51] further demonstrated that marital status was associated with food insecurity among men, but not women. In concert with these findings, we found significant associations between the marital status of the household head and risk of experiencing hunger, even after adjustments for sex, where living together/civil unions and never married increased the odds of experiencing hunger by 61% and 41%, respectively. We surmise that unmarried women in extended families may share food costs with other adults co-resident in the household; and, over generations, there is resilience and better coping strategies within the household as the burden of providing food is shared among members.

Contemporary considerations and current context

The present COVID-19 pandemic, occurring alongside household and child food and nutrition insecurity, presents a disconcerting syndemic [52] that is capable of reversing any recent mitigation of hunger at household and individual levels in the country [53]. Indeed, the COVID-19 pandemic has forced South Africa into an abrupt and prolonged lockdown threatening job and societal security [54]. Data from the National Income Dynamics Study-Coronavirus Rapid Mobile Survey (NID-CRAMS) [55], a four-wave longitudinal survey of adult South Africans, indicate that even with re-opening of the economy, lifting restrictions on movement and returning to work, 37-47% households did not have sufficient money to buy food, with 16-22% stating that they experienced hunger, during the period from May/June 2020 to February/March 2021 [55]. Overall,

while hunger declined somewhat over this period, the poorest quintile suffered more hunger than before [55]. Without augmented social supports for households with children and an emphasis on nutrition-sensitive interventions [40,49,56], it is likely that food insecurity will pervade [14,52] and possibly impede the country from realising its 2030 Sustainable Development Goal of achieving zero hunger [29,57]. As was evident in our sensitivity analysis when deciphering the differential risk of food insecurity between households with and without children, we surmise that these findings may indicate the preponderance of socioeconomic disadvantage among female-headed households. Affirmatively, Broussard [58] found that gender differences in income and education explain the majority of the gender gap in the prevalence of food insecurity.

Deleted: ,

Limitations

The present study is not without limitations. The SANHANES-1 was conducted in 2011/12. Although never repeated, the data are at least eight years old and may not reflect the current status of food security in South Africa. Nonetheless, the SANHANES provides the most recent and comprehensive dataset on food security at the national and household level using the CCHIP index that explores the context and loci within which food insecurity exists. This is in contrast to other surveys which only record the frequency of problems satisfying food needs "in the past 12 months" [59]. Secondly, a comprehensive assessment of socioeconomic status was beyond the scope of this research. Having considered the primary source of household income and educational status as contributing factors to household economic stability, a more robust measure of socioeconomic status using household assets could add a more granular dimension to the analysis.

Conclusions

Our study confirms that South African children face a major challenge of food insecurity that is driven by a number of household risk factors. The untoward effects of food insecurity on child and adolescent health are not evanescent and have far-reaching adverse health outcomes. Therefore, this is a clarion call to bolster multi-sectoral interventions and healthy public policies, with a strong focus on the social determinants of health and poverty eradication strategies that target households with greater vulnerability, such as those with children.

Acknowledgements

The authors acknowledge Dr Innocent Maposa for his assistance with data analysis.

References

1. United Nations Office of the High Commissioner for Human Rights. Fact Sheet No. 34, The Right to Adequate Food [Internet]. Refworld. 2021 [cited 2021 Jun 7]. Available from: https://www.refworld.org/docid/4ca460b02.html

2. Food and Agriculture Organization. The Right to Food around the Globe [Internet]. Food and Agriculture Organization of the United Nations. 2015 [cited 2021 Jun 6]. Available from: http://www.fao.org/right-to-food-around-the-globe/countries/zaf/en/

3. Skoet J, Stamoulis KG. The state of food insecurity in the world 2006 [Internet]. Food and Agriculture Organization. 2006 [cited 2021 Jun 7]. Available from: http://www.fao.org/3/a0750e/a0750e00.pdf

4. Neuman N, Eli K, Nowicka P. Feeding the extended family: gender, generation, and socioeconomic disadvantage in food provision to children. Food, Cult Soc [Internet]. 2019;22(1):45–62. Available from: https://doi.org/10.1080/15528014.2018.1547066

5. Fraval S, Hammond J, Bogard JR, Ng'endo M, van Etten J, Herrero M, et al. Food Access Deficiencies in Sub-saharan Africa: Prevalence and Implications for Agricultural Interventions. Frontiers in Sustainable Food Systems [Internet]. 2019 Nov 19 [cited 2021 Jun 14];3. Available from: https://www.frontiersin.org/articles/10.3389/fsufs.2019.00104/full

6. The World Bank Annual Report 2019: Ending Poverty, Investing in Opportunity [Internet]. Washington, DC: World Bank. 2019 [cited 2021 Jun 7]. Available from: https://openknowledge.worldbank.org/handle/10986/32333

7. Towards measuring food security in South Africa: An examination of hunger and food inadequacy [Internet]. Stats-SA. 2019. [cited 2021 Jun 7]. Available from: http://www.statssa.gov.za/publications/03-00-14/03-00-142017.pdf

8. Mark Nord Prevalence, Severity, and Household Characteristics Food Insecurity in Households with Children [Internet]. 2009. Available from: https://www.ers.usda.gov/webdocs/publications/44419/9360_eib56_1_.pdf?v=0#:~:text=Nearly%2016%20percent%20of%20households

9. Ngema P, Sibanda M, Musemwa L. Household Food Security Status and Its Determinants in Maphumulo Local Municipality, South Africa. Sustainability [Internet]. 2018 Sep 16 [cited 2021 Jun 14];10(9):3307. Available from: https://www.mdpi.com/2071-1050/10/9/3307

10. Webb P, Stordalen GA, Singh S, Wijesinha-Bettoni R, Shetty P, Lartey A. Hunger and malnutrition in the 21st century. BMJ [Internet]. 2018 Jun 13 [cited 2021 Jun 14];k2238. Available from: https://www.bmj.com/content/361/bmj.k2238

11. Shisana O, Labadarios D, Rehle T, Simbayi L, Zuma K, Dhansay A, Reddy P, Parker W, Hoosain E, Naidoo P, Hongoro C, Mchiza Z, Steyn NP, Dwane N, Makoae M, Maluleke T, Ramlagan S, Zungu N, Evans MG, Jacobs L, Faber M & S-1 T. South African National Health and Nutrition Examination Survey, 2012 [Internet]. Human Sciences Research Council. 2013 [cited 2021 Jun 7]. Available at: http://repository.hsrc.ac.za/bitstream/handle/20.500.11910/2864/7844.pdf?sequence=1&isAllowed=y

12. Shung-King M, Lake L, Sanders D, Hendricks M. Child and adolescent health: Leave no

one behind [Internet]. South African Child Gauge. 2019 [cited 2021 Jun 7]. Available from: http://www.ci.uct.ac.za/sites/default/files/image_tool/images/367/Child_Gauge/South_African_Child_Gauge_2019/ChildGauge_2019_final_print%20%28sm%29.pdf

13. Hall K, Mokomane Z. The shape of children's families and households: A demographic overview [Internet]. South African Child Gauge Child Fam State. 2018 [cited 2021 Jun 7]. Available from: http://www.ci.uct.ac.za/ci/child-gauge/2018

14. Perez-Escamilla R, Pinheiro De Toledo Vianna R, De Toledo Vianna P. Review of the Evidence. Journal of Applied Research on Children: Informing Policy for Children at Risk [Internet]. 3(1):9. Available from: https://files.eric.ed.gov/fulltext/EJ1189045.pdf

15. Seligman HK, Laraia BA, Kushel MB. Food Insecurity Is Associated with Chronic Disease among Low-Income NHANES Participants. The Journal of Nutrition [Internet]. 2009 Dec 23 [cited 2021 Jun 14];140(2):304–10. Available from: https://www.ncbi.nlm.nih.gov/pmc/articles/PMC2806885/

16. Kirkpatrick SI, McIntyre L, Potestio ML. Child Hunger and Long-term Adverse Consequences for Health. Archives of Pediatrics & Adolescent Medicine [Internet]. 2010 Aug 1 [cited 2021 Jun 14];164(8). Available from: https://jamanetwork.com/journals/jamapediatrics/fullarticle/383613

17. Ke J, Ford-Jones EL. Food insecurity and hunger: A review of the effects on children's health and behaviour. Paediatrics & Child Health [Internet]. 2015 Mar [cited 2021 Jun 14];20(2):89–91. Available from: https://www.ncbi.nlm.nih.gov/pmc/articles/PMC4373582/

18. Sparén P, Vågerö D, Shestov DB, Plavinskaja S, Parfenova N, Hoptiar V, et al. Long term mortality after severe starvation during the siege of Leningrad: prospective cohort study. BMJ [Internet]. 2004 Jan 3 [cited 2021 Jun 14];328(7430). Available from: https://www.bmj.com/content/328/7430/11.long

19. Lumey L, Stein AD, Kahn HS, van der Pal-de Bruin KM, Blauw G, Zybert PA, et al. Cohort Profile: The Dutch Hunger Winter Families Study. International Journal of Epidemiology [Internet]. 2007 Jun 25 [cited 2021 Jun 14];36(6):1196–204. Available from: https://pubmed.ncbi.nlm.nih.gov/17591638/

20. Calkins K, Devaskar SU. Fetal Origins of Adult Disease. Current Problems in Pediatric and Adolescent Health Care [Internet]. 2011 Jul [cited 2021 Jun 14];41(6):158–76. Available from: https://www.ncbi.nlm.nih.gov/pmc/articles/PMC4608552/

21. Bjerregaard LG, Jensen BW, Ängquist L, Osler M, Sørensen TIA, Baker JL. Change in Overweight from Childhood to Early Adulthood and Risk of Type 2 Diabetes. New England Journal of Medicine [Internet]. 2018 Apr 5 [cited 2021 Jun 14];378(14):1302–12. Available from: https://www.nejm.org/doi/full/10.1056/NEJMoa1713231

22. Labadarios D, Mchiza Z, Steyn NP, Gericke G, Maunder E, Davids Y, et al. Food security in South Africa: a review of national surveys. Bulletin of the World Health Organization [Internet]. 2011 Dec 1 [cited 2021 Jun 14];89(12):891–9. Available from: https://pubmed.ncbi.nlm.nih.gov/22271946/

23. Labadarios D, Steyn N, Maunder E, MacIntryre U, Gericke G, Swart R, et al. The National Food Consumption Survey (NFCS): South Africa, 1999. Public Health Nutrition [Internet]. 2005 Aug [cited 2021 Jun 14];8(5):533–43. Available from:

https://pubmed.ncbi.nlm.nih.gov/16153334/

24. Executive summary of the National Food Consumption Survey Fortification Baseline (NFCS-FB-I) South Africa, 2005 – ScienceOpen [Internet]. Scienceopen.com. 2020 [cited 2021 Jun 14]. Available from: https://www.scienceopen.com/document?vid=4b09aeb3-5ae5-4a93-9f73-73210a39ff8e

25. Chakona G, Shackleton CM. Household Food Insecurity along an Agro-Ecological Gradient Influences Children's Nutritional Status in South Africa. Frontiers in Nutrition [Internet]. 2018 Jan 22 [cited 2021 Jun 14];4. Available from: https://www.ncbi.nlm.nih.gov/pmc/articles/PMC5786884/

26. Waidler J, Devereux S. Social grants, remittances, and food security: does the source of income matter? Food Security [Internet]. 2019 May 20 [cited 2021 Jun 14];11(3):679–702. Available from: https://link.springer.com/article/10.1007/s12571-019-00918-x

27. Ruiters M, Wildschutt A. Food insecurity in South Africa: Where does gender matter? [Internet]. Agenda. 2012 [cited 2021 Jun 14]. Available from: https://www.tandfonline.com/doi/abs/10.1080/10130950.2010.10540516

28. Ruel MT, Alderman H. Nutrition-sensitive interventions and programmes: How can they help to accelerate progress in improving maternal and child nutrition? Lancet [Internet]. 2013;382(9891):536–51. Available from: http://dx.doi.org/10.1016/S0140-6736(13)60843-0

29. Pérez-Escamilla R. Food Security and the 2015–2030 Sustainable Development Goals: From Human to Planetary Health. Current Developments in Nutrition [Internet]. 2017 Jun 20 [cited 2021 Jun 14];1(7). Available from: https://pubmed.ncbi.nlm.nih.gov/29955711/

30. de Onis M. Development of a WHO growth reference for school-aged children and adolescents. Bulletin of the World Health Organization [Internet]. 2007 Sep 1 [cited 2021 Jun 14];85(09):660–7. Available from: https://pubmed.ncbi.nlm.nih.gov/18026621/

31. de Onis M, Garza C, Onyango AW, Rolland-Cachera M-F. WHO growth standards for infants and young children. Arch Pediatr, [online] 16(1), pp.47–53. Available at: https://www.sciencedirect.com/science/article/pii/S0929693X08005101?via%3Dihub [Accessed 24 Jun. 2021].

32. Kuruvilla S, Bustreo F, Kuo T, Mishra C, Taylor K, Fogstad H, et al. The Global strategy for women's, children's and adolescents' health (2016–2030): a roadmap based on evidence and country experience. Bulletin of the World Health Organization [Internet]. 2016 May 1 [cited 2021 Jun 24];94(5):398–400. Available from: https://www.ncbi.nlm.nih.gov/pmc/articles/PMC4850541/#:~:text=article's%20original%20URL.-,The%20Global%20strategy%20for%20women's%2C%20children's%20and%20adolescents'%20health%20(,well%2Dbeing%20in%20all%20settings.

33. Swindale A, Bilinsky P. Development of a Universally Applicable Household Food Insecurity Measurement Tool: Process, Current Status, and Outstanding Issues. The Journal of Nutrition [Internet]. 2006 May 1 [cited 2021 Jun 14];136(5):1449S1452S. Available from: https://academic.oup.com/jn/article/136/5/1449S/4670078

34. Drimie S, Ruysenaar S. The integrated food security strategy of South Africa: An institutional analysis. [Internet]. 2019 [cited 2021 Jun 14]. Available from: https://www.semanticscholar.org/paper/The-Integrated-Food-Security-Strategy-of-South-

An-Drimie-Ruysenaar/a1047527a4d5a7e8eeb27b220e766533ede97fe1

35. Farrell P, Thow AM, Abimbola S, Faruqui N, Negin J. How food insecurity could lead to obesity in LMICs. Health Promotion International [Internet]. 2017 May 24 [cited 2021 Jun 14];33(5):812–26. Available from: https://pubmed.ncbi.nlm.nih.gov/28541498/#:~:text=While%20food%20insecurity%20is%20a,also%20been%20linked%20with%20obesity.&text=Affordability%20of%20high%2Denergy%2C%20processed,%2D%20and%20middle%2Dincome%20countries.

36. Chinnakali P, Upadhyay RP, Shokeen D, Singh K, Kaur M, Singh AK, et al. Prevalence of household-level food insecurity and its determinants in an urban resettlement colony in north India. Journal of health, population, and nutrition [Internet]. 2014 [cited 2021 Jun 14];32(2):227–36. Available from: https://www.ncbi.nlm.nih.gov/pmc/articles/PMC4216959/

37. Mortazavi Z, Dorosty AR, Eshraghian MR, Ghaffari M, Ansari-Moghaddam A, Mohammadi M. Household Food Insecurity in Southeastern Iran: Severity and Related Factors. International Journal of Food Science [Internet]. 2017 [cited 2021 Jun 14];2017:1–7. Available from: https://www.hindawi.com/journals/ijfs/2017/7536024/

38. Tadesse Tantu A, Demissie Gamebo T, Kuma Sheno B, Yohannis Kabalo M. Household food insecurity and associated factors among households in Wolaita Sodo town, 2015. Agriculture & Food Security [Internet]. 2017 Feb 27 [cited 2021 Jun 14];6(1). Available from: https://agricultureandfoodsecurity.biomedcentral.com/articles/10.1186/s40066-017-0098-4

39. Sisha TA. Household level food insecurity assessment: Evidence from panel data,

Ethiopia. Scientific African [Internet]. 2020 Mar [cited 2021 Jun 14];7:e00262. Available from: https://www.sciencedirect.com/science/article/pii/S2468227619308233

40. Hossain M, Choudhury N, Adib Binte Abdullah K, Mondal P, Jackson AA, Walson J, et al. Evidence-based approaches to childhood stunting in low and middle income countries: a systematic review. Archives of Disease in Childhood [Internet]. 2017 May 3 [cited 2021 Jun 14];102(10):903–9. Available from: https://pubmed.ncbi.nlm.nih.gov/28468870/

41. Vaezghasemi M, Öhman A, Eriksson M, Hakimi M, Weinehall L, Kusnanto H, et al. The Effect of Gender and Social Capital on the Dual Burden of Malnutrition: A Multilevel Study in Indonesia. Noor AM, editor. PLoS ONE [Internet]. 2014 Aug 25 [cited 2021 Jun 14];9(8):e103849. Available from: https://journals.plos.org/plosone/article?id=10.1371/journal.pone.0103849

42. Tarasuk V, Fafard St-Germain A-A, Mitchell A. Geographic and socio-demographic predictors of household food insecurity in Canada, 2011–12. BMC Public Health [Internet]. 2019 Jan 3 [cited 2021 Jun 14];19(1). Available from: https://bmcpublichealth.biomedcentral.com/articles/10.1186/s12889-018-6344-2

43. Brown EM, Tarasuk V. Money speaks: Reductions in severe food insecurity follow the Canada Child Benefit. Preventive Medicine [Internet]. 2019 Dec [cited 2021 Jun 14]. Available from: https://www.sciencedirect.com/science/article/pii/S0091743519303524

44. Costa NS, Santos MO, Carvalho CPO, Assunção ML, Ferreira HS. Prevalence and Factors Associated with Food Insecurity in the Context of the Economic Crisis in Brazil. Current Developments in Nutrition [Internet]. 2017 Sep 7 [cited 2021 Jun 14];1(10):e000869. Available from: https://academic.oup.com/cdn/article/1/10/e000869/4653477

45. Coleman-Jensen A, Nord M, Andrews M, Carlson S. Household Food Security in the United States in 2010. SSRN Electronic Journal [Internet]. 2011 [cited 2019 Mar 22]; Available from: https://papers.ssrn.com/sol3/papers.cfm?abstract_id=2116606

46. Kim J, Lim H. Nutritional Management in Childhood Obesity. Journal of Obesity & Metabolic Syndrome [Internet]. 2019 Dec 30 [cited 2021 Jun 24];28(4):225–35. Available from: https://www.ncbi.nlm.nih.gov/pmc/articles/PMC6939706/

47. Gebremedhin S. Prevalence and differentials of overweight and obesity in preschool children in Sub-Saharan Africa. BMJ Open [Internet]. 2015 Dec [cited 2021 June 24];5(12):009005. Available from: https://bmjopen.bmj.com/content/5/12/e009005.long

48. Devereux S, Hochfeld T, Karriem A, Mensah C, Morahanye M, Msimango T, et al. Food Security SA Working Paper Series: #004 School Feeding in South Africa: What we know, what we don't know, what we need to know, what we need to do Authors [Internet]. ; Available from: https://foodsecurity.ac.za/wp-content/uploads/2018/06/CoE-FS-WP4-School-Feeding-in-South-Africa-11-jun-18.pdf

49. Qar Z, Bhutta A, Das JK, Rizvi A, Gaff MF, Walker N, et al. Maternal and Child Nutrition 2 Evidence-based interventions for improvement of maternal and child nutrition: what can be done and at what cost? The Lancet Nutrition Interventions Review Group, and the Maternal and Child Nutrition Study Group. www.thelancet.com [Internet]. 2013 [cited 2021 Jun 6];382. Available from: http://dx.doi.org/10.1016/

50. Power M, Uphoff EP, Stewart-Knox B, Small N, Doherty B, Pickett KE. Food insecurity and socio-demographic characteristics in two UK ethnic groups: an analysis of women in the Born in Bradford cohort. Journal of Public Health [Internet]. 2017 Mar 28 [cited 2021

Jun 14];40(1):32–40. Available from: https://academic.oup.com/jpubhealth/article/40/1/32/3091690

51. Hanson KL, Sobal J, Frongillo EA. Gender and Marital Status Clarify Associations between Food Insecurity and Body Weight. The Journal of Nutrition [Internet]. 2007 Jun 1 [cited 2021 Jun 14];137(6):1460–5. Available from: https://academic.oup.com/jn/article/137/6/1460/4664800

52. Pérez- Escamilla R, Cunningham K, Moran VH. COVID- 19 and maternal and child food and nutrition insecurity: a complex syndemic. Maternal & Child Nutrition [Internet]. 2020 Jun 16 [cited 2021 Jun 14];16(3). Available from: https://onlinelibrary.wiley.com/doi/full/10.1111/mcn.13036

53. Key Indicator Report STATS SA [Internet].; Available from: https://www.statssa.gov.za/publications/Report%2003-00-09/Report%2003-00-092016.pdf

54. International Labour Organization. ILO Monitor: COVID-19 and the world of work. Third edition U [Internet]. 2020. 1–23 p. Available from: https://www.ilo.org/wcmsp5/groups/public/---dgreports/---dcomm/documents/briefingnote/wcms_743146.pdf

55. Patel L, Bridgman G. Hunger in South Africa during 2020: Results from Wave 3 of NIDS-CRAM [Internet]. CRAM. 2021 [cited 2021 Jun 7]. Available from: https://cramsurvey.org/wp-content/uploads/2021/02/10.-Van-der-Berg-S.-Patel-L.-Bridgman-G.-2021-Hunger-in-South-Africa-during-2020-Results-from-Wave-3-of-NIDS-CRAM-1.pdf

56. Bhutta ZA, Das JK, Rizvi A, Gaffey MF, Walker N, Horton S, et al. Evidence-based interventions for improvement of maternal and child nutrition: what can be done and at what cost? The Lancet [Internet]. 2013 Aug [cited 2021 Jun 14];382(9890):452–77. Available from: https://www.thelancet.com/journals/lancet/article/PIIS0140-6736(13)60996-4/fulltext

57. Gebre GG, Isoda H, Amekawa Y, Rahut DB, Nomura H, Watanabe T. What Explains Gender Gaps in Household Food Security? Evidence from Maize Farm Households in Southern Ethiopia. Social Indicators Research [Internet]. 2021 Jan 5 [cited 2021 Jun 24];155(1):281–314. Available from: https://link.springer.com/article/10.1007/s11205-020-02600-8

58. Broussard NH. What explains gender differences in food insecurity? Food Policy [Internet]. 2019 Feb [cited 2021 Jun 14]; 83:180–94. Available from: https://www.sciencedirect.com/science/article/pii/S0306919218300824#:~:text=The%20preference%20to%20ensure%20the,bargaining%20power%20within%20the%20household

59. Neupane S, K.C. P, Doku DT. Overweight and obesity among women: analysis of demographic and health survey data from 32 Sub-Saharan African Countries. BMC Public Health [Internet]. 2015 Dec [cited 2021 Jun 24];16(1). Available from: https://bmcpublichealth.biomedcentral.com/articles/10.1186/s12889-016-2698-5

Supporting information

S1 Fig. Database management, organisation and integration of data extracted from the four household and individual level datasets of the SANHANES-1

S1 Table. Weighted univariable multinomial logistic regression analysis of factors associated with being at risk of hunger and experiencing hunger against being food secure

S2 Table. Weighted multivariable multinomial logistic regression analysis of factors associated with being at risk of hunger and experiencing hunger against being food secure in households without children

S3 Table. Weighted multivariable multinomial logistic regression analysis of factors associated with being at risk of hunger and experiencing hunger against being food secure in households with children

S1 Dataset: Final analytic sample of households with food security categories

Fig1

Click here to access/download;Figure;Fig 1.tif

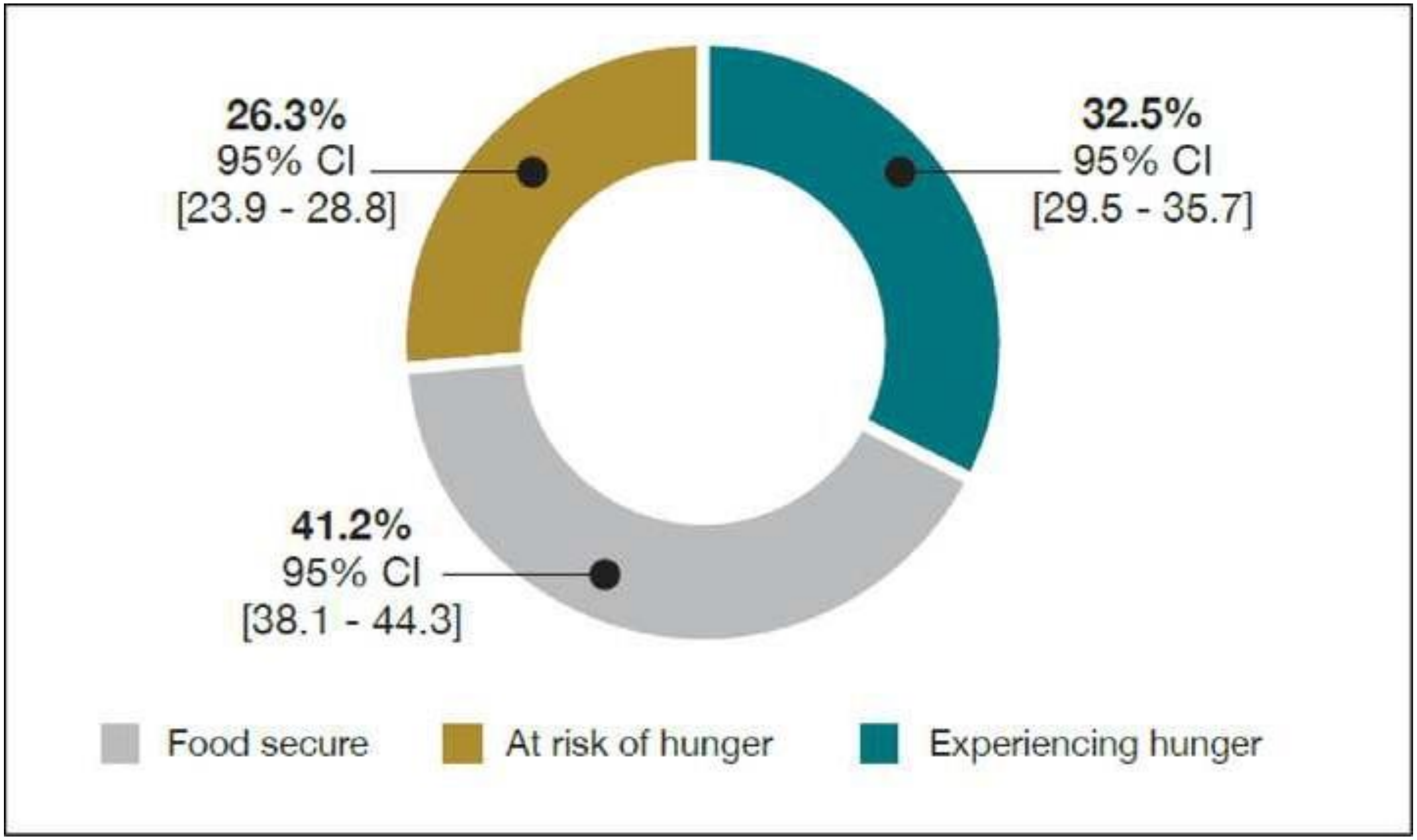

Fig2A

Click here to access/download;Figure;Fig 2A.tif

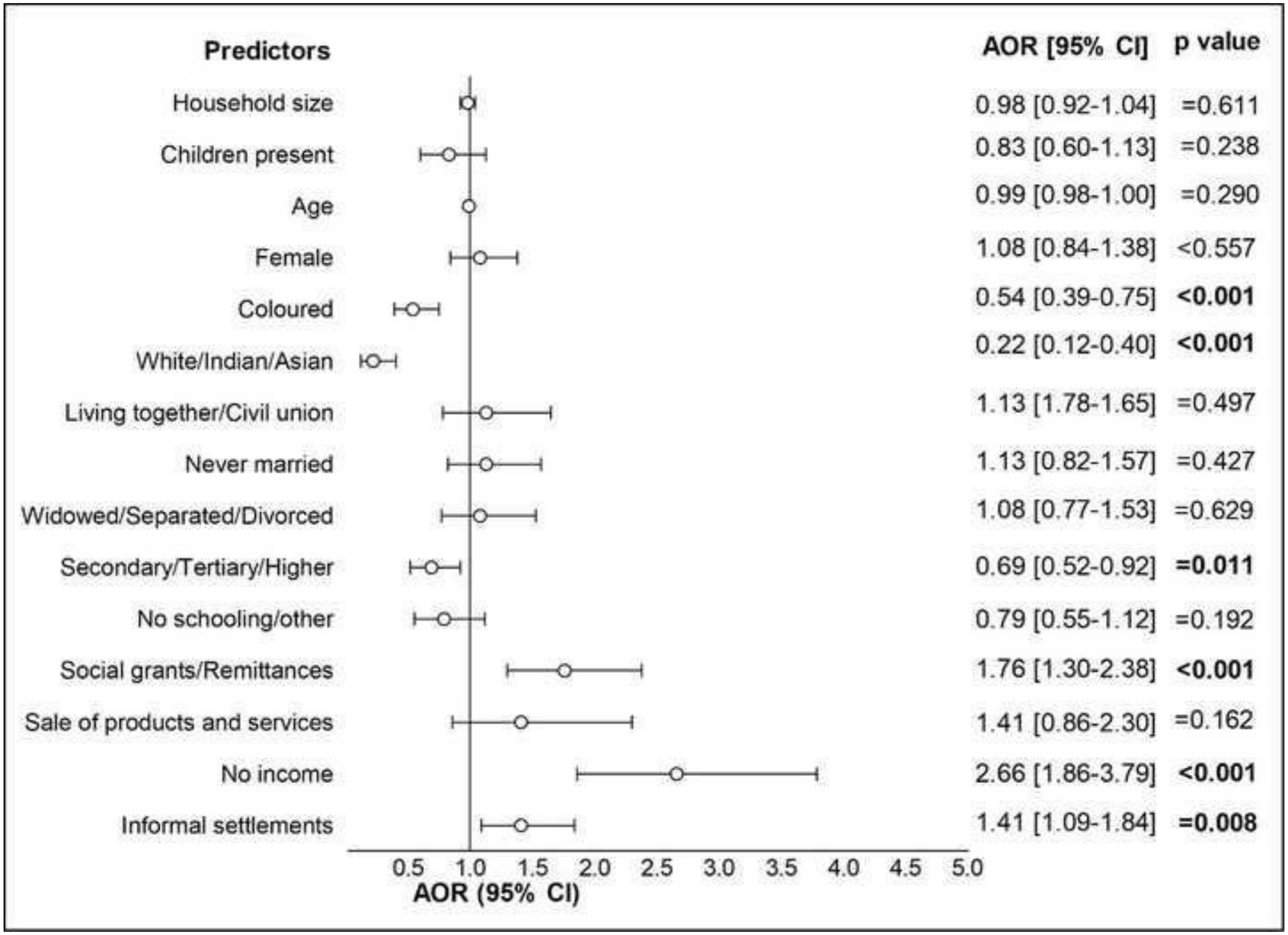

Fig2B

Click here to access/download;Figure;Fig 2B.tif

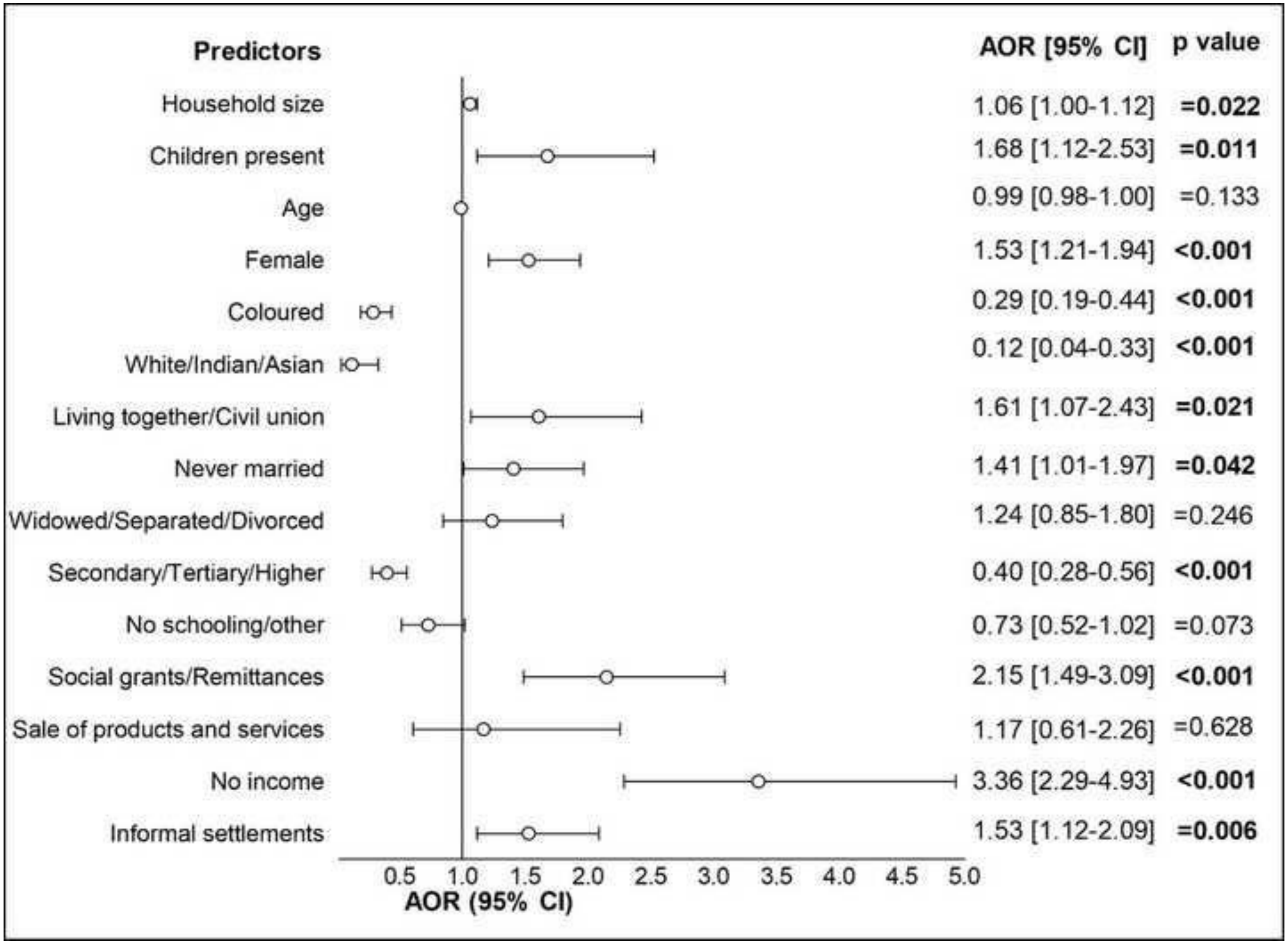

Supporting Information_S1_Fig

Click here to access/download
Supporting Information
S1 Fig.docx

Supporting Information_S1_Table

Click here to access/download
Supporting Information
S1 Table.docx

Supporting Information_S2_Table

Click here to access/download
Supporting Information
S2 Table.docx

Supporting Information_S3_Table

Click here to access/download
Supporting Information
S3 Table.docx

Supporting Information_S1_data

Click here to access/download
Supporting Information
S1 Database.csv

Revised Manuscript with Track Changes

Childhood food insecurity in South Africa: A household-level analysis of hunger

Siluleko A. Mkhize[1], Elena Libhaber[2], Ronel Sewpaul[3], Priscilla Reddy[3,4] and Laurel Baldwin-Ragaven[1]*

Formatted: No underline

[1]*Department of Family Medicine and Primary Care, School of Clinical Medicine, Faculty of Health Sciences, University of the Witwatersrand, Johannesburg, South Africa*

[2]*School of Clinical Medicine and Health Sciences Research Office, Faculty of Health Sciences, University of the Witwatersrand, Johannesburg, South Africa*

[3]*Health and Wellbeing, Human and Social Capabilities Division, Human Sciences Research Council, Cape Ttown, South Africa*

[4]*Faculty of Health Sciences, Nelson Mandela University, Port Elizabeth, South Africa*

***Corresponding author:** Professor Laurel Baldwin-Ragaven, University of the Witwatersrand, 29 Princess of Wales Terrace, Parktown, Johannesburg, 2193

Telephone: 011 717 2058

Email: laurel.baldwin-ragaven@wits.ac.za

Acknowledgements

The authors acknowledge Dr Innocent Maposa for his assistance with data analysis.

Data availability statement

The final de-identified anonymised dataset used in this study is provided as a supporting information file to this manuscript, entitled *Final Analytic Sample of Households with Food Security Categories* (S1 File). A Access to the data was granted through a data sharing agreement was signed with the Human Sciences Research Council (HSRC), , the entity that conducted the data curators of the SANHANES survey. Adult SANHANES data are available through registered access from the HSRC data repository at: http://curation.hsrc.ac.za/Datasets-XKAHAA.phtml. The SANHANES adult data are available through registered access from the HSRC data repository at http://curation.hsrc.ac.za/Datasets-XKAHAA.phtml. The other Child

data subsets are under third party rights and have not been publicly made availablecan be accessed upon reasonable request by contacting the HSRC at datahelp@hsrc.ac.za @hsrc.ac.za. Further information on how to access these datasets can be obtained by contacting Ronel Sewpaul at rsewpaul@hsrc.ac.za. The final dataset used in this study is available upon request to the corresponding author.

Conflicts of interest

None declared

Contributions

SM, LBR and EL conceptualised the study and its design. SM, EL, LBR and RS conducted and interpreted the analyses. SM wrote the first draft of the manuscript; and, EL LBR and RS worked on subsequent iterations of the manuscript. RS and PR critically revised the manuscript for important intellectual content. All authors approved the final manuscript and take primary responsibility for its contents. The contents of the manuscript are solely the responsibility of the authors and do not necessarily represent the official views of the HSRC or the University of the Witwatersrand, Johannesburg, South Africa.

ORCID

Siluleko Mkhize https://orcid.org/ 0000-0002-8351-6891

Elena Libhaber https://orcid.org/ 0000-0002-7043-4002

Laurel Baldwin-Ragaven https://orcid.org/0000-0002-6744-3768

Ronel Sewpaul https://orcid.org/ 0000-0002-2523-1222

Priscilla Reddy https://orcid.org/ 0000-0002-0432-9060

Childhood food insecurity in South Africa: A household-level analysis of hunger

Abstract

Food insecurity impacts childhood nutritional status, physical and cognitive development, and increases lifetime risk for chronic disease. Previous South African studies have examined hunger at sub-national level without a specific focus on children. We therefore determined the national prevalence of childhood food insecurity and identified factors associated with hunger within the household. Individual and household-level data were extracted from the South African National Health and Nutrition Examination Survey (SANHANES-1). Prevalence of food insecurity was assessed using the Community Childhood Hunger Identification Project (CCHIP) index. Multinomial logistic regression analyses were conducted on all households (with and without children) to determine the predictors of food insecurity. Of 5 098 households surveyed, 68.6% had children (0-19 years). Of the households with children, 32.5% [95% CI (29.5 – 35.7)] were experiencing hunger and 26.3% [95% CI (23.9 – 28.8)] were at risk of hunger. Among all the households, significant associations for households experiencing hunger were the presence of children: AOR [95% CI]: 1.68 [1.12 – 2.53]; being female-headed: AOR [95% CI]: 1.53 [1.21 – 1.94] and informally-located; AOR [95% CI]: 1.61 [1.07 – 2.43]. Having a non-African household head, Coloured: AOR [95% CI]: 0.29 [0.19 – 0.44] and White/Indian/Asian: 0.12 [0.04 – 0.33] conferred lower odds of experiencing hunger and, as well as the household head having secondary/tertiary education conferring lower odds of experiencing hunger; AOR [95% CI]: 0.40

[0.28 – 0.56] and being at risk of hunger; AOR [95% CI]: 0.69 [0.52 – 0.92] respectively. Receiving social grants, pensions or remittances more than doubled the risk of experiencing hunger; AOR [95% CI]: 2.15 [1.49 – 3.09]. In summary, only 41% of South African households with children were food secure., necessitating multi-sectoral intervention and healthy public policies. These findingsis indicates a critical need for multi-sectoral interventions to bolster sustainable food systems for households with children, and to improve socialpublic protections for whose heads are either female-headed, or African-headed and, informally-located and households dependent on social grants.

Keywords: children, household, food insecurity, hunger, CCHIP index, South Africa, SANHANES

Introduction

Food insecurity has emerged as a major public health concern; and, the obligation to realise the "*right to food and basic nutrition*" is enshrined in both the United Nations charter [1](1) and the South African constitution [2] (1). The Food and Agriculture Organization (FAO) of the United Nations states that, "food security exists when all people, at all times, have physical, social and economic access to sufficient, safe and nutritious food that meets their dietary needs and food preferences for an active and healthy life" ([3]2), emphasising that the availability of food must be beyond mere subsistence (3). In Sub-Saharan Africa, one in four people were undernourished in 2017 which represents about one third of the 821 million people suffering from chronic hunger globally (4). According to actuarial projections, Africa will be home to 90% of the world's poorest by 2030 (4,5).

In 2017, 6.8 million South Africans were affected by hunger (6). Although this number has halved from 13.5 million in 2002, about 1.7 million households across the country remain food insecure (6). Moreover, a considerable but undocumented proportion of these households comprise children and adolescents below the age of 20 years, who are vulnerable to food deprivation and hunger (3). Indeed, the presence of children in households may confer additional "*pressure of mouths to feed*" (7,8). The present COVID-19 pandemic, occurring alongside household and child food and

~~nutrition insecurity, presents a disconcerting syndemic (9,10) capable of reversing the improvements witnessed between 2002-2017 (6). Indeed, the pandemic has forced South Africa into an abrupt and prolonged lockdown threatening job and societal security (11,12); therefore, it is likely that food insecurity will pervade (13,14) and possibly impede the country from realising its 2030 Sustainable Development Goal of achieving zero hunger (15).~~

Throughout the world, household food insecurity is associated with malnutrition, stunting and wasting among children (16). The largest national census of South African households, the South African National Health and Nutrition Examination Survey (SANHANES), reported in 2011/12 that 23.5% of children aged 0-14 years were malnourished (stunted, wasted or undernourished) (20). In 2019, Sung-King, et al (18), using different methodology, found that ~48.8% of children under five years were malnourished. Nationally~~In South Africa~~, severe-acute and moderate-acute malnutrition accounts for between 4% (19) to 25% of child mortality (17,18). ~~In 2015, malnutrition contributed 4% to under-five mortality (19). According to Sung-King (18), ~48.8% of children under-five years were malnourished and the South African National Health And Nutrition Examination Survey (SANHANES) in 2011/12 reported that 51.5% of children aged 0-14 years were malnourished~~ (20). Hunger has profound health consequences that not only affect physical growth and cognitive development in childhood (19,20), but endure throughout the life-course, into adulthood (21,22) and moreover extend into subsequent generations (23,24). Two longitudinal cohort studies of the consequences of hunger that began in the throes of the second World War, the Dutch Winter Famine (24) and the siege of Leningrad (23,25), indicate that the effects of intra-uterine exposure to food insecurity can traverse through multiple generations, predisposing to a "thrifty phenotype" among progeny. Bjerregaard (28) found that another form of malnutrition, obesity in childhood (measured at ages 7 and 13 years), significantly increased the hazards of developing type-2 diabetes in late adulthood among Danish men. Conversely, reversal of obesity in adolescence reduced the risk by 4%, a finding that may be explained, in part, by food insecurity given that obesity and type-2 diabetes are primarily diet-sensitive adverse health outcomes (19,20).

In South Africa, three national surveys conducted ~~in~~ between 1999 and ~~-~~2008 estimated the prevalence of food insecurity using the Community Childhood Hunger Identification Project

Commented [LB1]: Correct this reference: should be SANHANES

Formatted: Not Highlight

(CCHIP) index (27,28); the National Food Consumption Surveys of 1999 and 2005, and the South African Social Attitudes Survey of 2008. A review of Tthese studies, which included children from 1-9 years old with at least one adult in the household, indicated a marked decline of household food insecurity from 52.3% in 1999 to 25.9% in 2008 (22,23,24). These studies looked at a segment of children (those 1-9 years old). None of these studies, however, distinguished between households with and without children thus perhaps underestimating the true extent of childhood food insecurity (29–31). Much of what is known about the sociodemographic correlates of food insecurity in South Africa has been gleaned from sub-national cross-sectional studies. Food insecurity measured using the Household Food Insecurity Access Scale (HFIAS) (32,33) was markedly highindicated a gradient across the rural-urban continuum of South African towns, with households in rural areas reporting higher scores, and therefore higher more food insecurity, than peri-urban households (households located in urban-informal settlements) (34). Households that are reliant on private food charities and government assisted social grants are at an three-fold increased risk of food insecurity (35). It is speculated that while social grants are regular and predictable, they are, however, not sensitive to the changing needs of a household (31,36). The gender of the household head is also an important factor predicting household vulnerabilities, with the highest prevalence of food insecurity in South Africa reported among female-headed households (31,35).

There is a paucity of nationally representative data to assess the from which the granular detail about prevalence of food insecurity prevalence can be determined across the country. The critical need to address hunger in childhood necessitates particular attention to factors affecting food insecurity in households with children and adolescents, so that. There is an urgent need for nutrition-sensitive interventions can be developed and/or strengthened (Ruel & Alderman, 2013). Such interventions would address the underlying determinants of child hunger, and include, for example, multi-sectoral approaches to social safety netting through minimum guaranteed income and/or cash transfers; financial, educational and parenting support to female-headed households with children; and, bolstering health services and sustainable agricultural practices (new REFS added). Such multi-pronged approaches are necessary to mediate the short-term, long-term and inter-generational adverse health consequences of food insecurity on childhood health. Therefore, drawing from a large-scale population-based sample of households, the SANHANES-1, we set out

Commented [LB2]: Why only two references?

Field Code Changed

to determine the prevalence of food insecurity in households with and without children as well as to assess associations between the sociodemographic characteristics of the household head and degrees of hunger.

Methods

Study design

We conducted a secondary data analysis of the SANHANES-1 (Shisana, et al 2013). The SANHANES-1 is a cross-sectional nationally representative sample of households that employed a multi-stage disproportionate, stratified cluster sampling approach based on the 2001 census Enumeration Areas (EAs). The first 20 households were sampled from each of the 500 EAs yielding a sample of 10 000 households. ~~Of these households, 9 423 valid households consisting of 26 819 non-institutionalised civilian adults and children were included in the study.~~ Ensuring national representativeness, the SANHANES-1 EA sampling was layered by type of locality (urban/rural; formal/informal), and province (there are nine in South Africa) as well as by “race” in formal urban areas. Race allows investigation of ongoing health disparities that have endured post-apartheid. In this way, the SANHANES captures the sociodemographic and economic profile of the country. Additional sample selection details have been published elsewhere (Shisana, et al 2013) (20). In our study, we used the “household” as the unit of analysis. In South Africa, a household is defined as “a group of people living in the same dwelling and sharing food from the same cooking pot” (6). In the SANHANES-1, household membership was attributed to persons who occupied the same dwelling and slept in the household for at least four nights a week. The household head was identified as a physically present member who was designated internally as the head of that household, and also served as the main respondent for household-level data acquisition.

Data extraction and study population

The SANHANES-1 database includes people of all ages residing in households in South Africa. For the present study, only occupied households were included ~~.~~(see S1 Fig). Five separate but inter-related SANHANES-1 questionnaires were administered at household-level by a trained field

worker capturing information on everyone living in that household (between 1 and 20+ people). The variables of interest for this study were extracted from each of the five questionnaires: visiting point (household) questionnaire (administered to the household head); child questionnaire (0-14 years old); child clinical examination form (0-14 years old); adult questionnaire (administered to anyone ≥ 15 years old); and, the adult clinical examination form (≥ 15 years old). After extraction, these data were entered into four separate newly created databases. Children, whom we defined as aged 0-19 years old, , and adults aged ≥20 years old, were extracted from both the child (<15 years) and adult (≥15 years) individual-level datasets respectively.and adults aged ≥20 years old were extracted from the child (<15 years) and adult (≥15 years) individual-level datasets, This age selection was done to align with global initiatives that target child and adolescent public health (REF) as well as in keeping with the World Health Organization multicentre growth reference standards for assessing anthropometry in children and adolescents (new REFS added). After merging data for individuals aged 15-19 with data from those 0-14 years old, thereby reclassifying everyone aged 0-19 years old as children, (37) we then after which they were reconciled them back into their corresponding households. In keeping with the World Health Organisation multicentre growth reference standards for assessing anthropometry in children, we re-defined children as 0-19 years old instead of the traditional 0-14 age classification used in the SANHANES and other epidemiological surveys. The final sample that we analysed included 5 098 households and their occupants, both adults and children. **Supplemental Figure 1S1 Fig** illustrates the database management, organisation and integration of the different household and individual-level variables from the five SANHANES-1 databases.

Formatted: Not Highlight

Ethical considerations

The present study received ethics approval from the Human Research Ethics Committee (HREC) (Medical) of the University of the Witwatersrand, Johannesburg, South Africa (Clearance certificate number: M180775).

Variables

Household composition and sociodemographic parameters

At household-level, the parameters included were household size, the number of adults and the number of children occupying a household. Age, self-reported sex (male and female), self-reported race (African, Coloured, White/Indian/Asian), educational attainment (no schooling, primary, secondary, tertiary/higher degrees), marital status (married, living together/civil union, never married, widowed, separated/divorced) and main source of income (salaries/wages, social grants/remittances/pensions, sale of products and services, no income) of all household heads were also included. Locality (urban, rural, formal and informal) was pre-assigned to the household from the EA sampling.

Household food insecurity

Food insecurity was measured using the CCHIP index, a validated tool for the assessment of food insecurity at the household level (38). While five of the eight questions are child-referenced, making the CCHIP a specific tool for measuring *childhood* food insecurity, the CCHIP was also administered to households without children, generalising the child-referenced questions to everyone in the household. A score of ≥5 affirmative responses indicates the presence of hunger, understood as "*experiencing hunger*" and recorded as such. A score of 1-4 affirmative responses indicates that the household is "*at risk of hunger*". Lastly, a score of zero indicates that the household is "*food secure*".

Statistical analyses

STATA software, version 15.1 (STATA Institute Inc., College ~~s~~Station, TX, USA) was used for database management and statistical analyses. Results are presented as frequencies and percentages for categorical variables and Median [IQR] or Mean ± SD in the case of continuous variables. Comparisons between households with and without children were performed using Mann-Whitney U tests for continuous non-normally distributed data. Categorical variables were compared using Chi-Square tests. All analyses were weighted to offset for the over-allocation of EAs in areas where Indian, Coloured or White racial groups prevailed, to ensure that the minimum required sample size in those minority groups were obtained. All analyses were performed using *svy* function in STATA, incorporating sample weights and stratified cluster sampling design to provide estimates. Weighted prevalence of food security in households with children, stratified by sociodemographic characteristics of the household heads was ascertained from frequencies and percentages; however, age differences in prevalence estimates were computed from a Kruskal-

Wallis test with a Bonferroni *post hoc* correction to adjust for multiple comparisons. To improve statistical power in the regression models, locality was dichotomi~~z~~sed to formal vs informal; educational attainment (primary, secondary/tertiary/higher degrees and no schooling); marital status (married, never married, living together/civil unions and widowed/separated/divorced). Weighted univariable and multivariable multinomial logistic regression analyses with robust standard errors were carried out to determine factors associated with being “at risk of hunger” and “experiencing hunger”, where the “food secure” category was used as the referent group for comparison in all households, and separately in households with and without children. Statistical significance was set at $p<0.05$. Multivariable multinomial logistic regression for “risk of hunger” and “experiencing hunger” using adjusted odds ratios with their 95% confidence intervals as measures of association are presented as forest plots, which were produced using GraphPad Prism, version 7.00 for Windows (GraphPad Software, La Jolla, California USA).

Results

Household composition, ~~and~~ living arrangements and main source of income of household heads

Table 1 shows the characteristics of South African households with and without children, and the living arrangements of the heads of households. Of the 5 098 households with complete CCHIP scores, 3 499 (68.6%) households contained children aged 0-19 years. Households with children were occupied by 9 505 adults and 9 184 children, while those without children were occupied by 3 243 adults. Households with children were 2.5 times larger than those without children (p<0.00~~0~~1). Forty percent of all households in South Africa contained children and were female headed. Of the households containing children, nine were child-headed. When comparing households with and without children, the proportions of African- and Coloured-headed households were not different (p=0.417~~1~~) and, no differences between urban formal and urban informal settings were found following two by two comparisons (p=0.361~~07~~). Furthermore, there was no statistically significant difference in the main source of income for households with and without children (p=0.381).

Table 1: Characteristics of occupied South African households with and without children, and sociodemographic characteristics of household heads.

	All Households AllN=5 098		p value
	With children Nn=3 499	Without children Nn=1 599	
Household characteristics			
Household size (n=21 932)	5 [4 – 6]	2 [1 – 2]	<0.0001
No. of Adults, ≥20 years (n=12 748)	2 [2 – 3]	2 [1 – 2]	<0.0001
No. of Children, <20 years (n=9 183)	2 [1 – 3]	-	-
**Gender (n=5 092)**			
Male	1 532 (31.77)	899 (15.76)	<0.0001
Female	1 963 (40.44)	698 (12.02)	
**Age, years (n=5 093)**			
Age	49.22 ± 14.67	52.07 ± 15.91	<0.0001
**Race (n=5 090) †**			
African	2 465 (60.88) [a]	849 (19.86)	<0.0001
Coloured	659 (6.86) [b]	263 (2.48)	
White/Indian/Asian	373 (4.54) [ab]	481 (5.39)	
**Locality (n=4 889) †**			
Urban formal	1 838 (39.25) [ab]	892 (16.15)	<0.0001
Urban informal	464 (7.1) [cd]	155 (2.54)	
Rural formal	359 (4.16) [ace]	222 (6.3)	
Rural informal	838 (22.82) [bde]	121 (1.68)	
**Marital status (n=4 998) †**			
Married	1 678 (34.68) [a]	687 (11.15)	<0.0001
Living together/civil union	310 (6.1) [bc]	73 (1.28)	
Never married	757 (16.02) [abd]	486 (9.5)	
Widowed	522 (11.38) [d]	236 (4.07)	

Separated/divorced	156 (3.9) [c]	93 (1.93)	
**Source of income (n=4 608)**			
Salaries or wages	1 330 (29.52)	591 (11.00)	
Social grants/pensions/remittances	1 006 (24.7)	498 (9.49)	0.38~~105~~
Products and services	138 (3.33)	78 (1.71)	
No income	644 (13.98)	323 (6.27)	

Data presented as median [IQR]; mean ± SD and n (%): unweighted (n) and weighted (%).
†Multiple 2x2 comparisons with Bonferroni correction: p<0.05 between households with and without children if categories share the same letter. Race and locality: p<0.00~~0~~1 for both (a) and (b) comparisons, Marital status: p=0.001 in married vs never married and living together vs never married whereas p=0.036 in Separated/divorced vs living together/civil unions and lastly p=0.013 in never married vs widowed.

Prevalence of food insecurity in households with children

In households with children, the prevalence of *experiencing hunger* and being *at risk of hunger* was 32.5% [95% CI (29.5 – 35.7)] and 26.3% [95% CI (23.9 – 28.8)], respectively (**Figure 1**). **Table 2** shows weighted prevalence of food insecurity stratified by household head sociodemographic characteristics. Households in which the household head was female, older, of African race, unmarried, not having a formal education, dependent on social grants/pensions/~~ and other~~ remittances and having no income showed a higher prevalence of experiencing hunger or being at risk of hunger (p<0.00~~0~~1). A further analysis of the collapsed locality categories (formal vs informal) yielded the following: 37.2% of households with children were informally located~~;~~ and, of these, only 28.9% were food secure. In contrast, of the 62.9% of households with children located in formal areas, 53.6% were food secure (p<0.00~~0~~1).

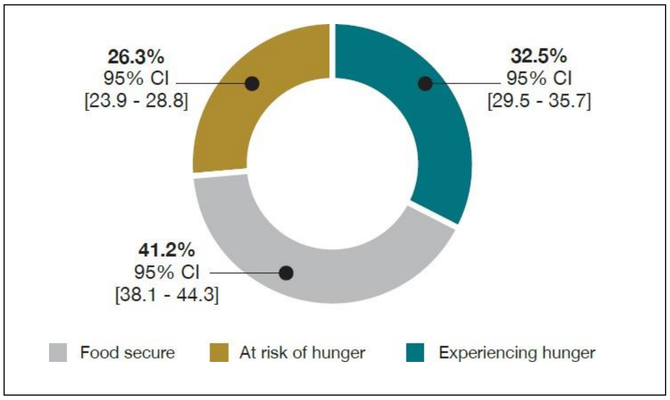

Figure 1: ~~Chart depicting the n~~National prevalence of food insecurity in households with children

Table 2: Weighted prevalence of household food security, stratified by sociodemographic characteristics of designated household heads of South African households with children.

	Total N	Food secure n (%)	†At risk of hunger n (%)	ƚExperiencing hunger n (%)	p value
**Gender (n=3 495)**					
Male	1 532	813 (22.16)	356 (11.09) a	363 (10.75) a	<0.00~~0~~1
Female	1 963	739 (19.07)	502 (15.13) a	722 (21.8) a	
**Age, years (n=3 499)**					
Age (Mean±SD)	3 499	47.87±13.85 *	49.76±15.15	50.70±15.25	0.00~~0~~1
**Race (n=3 497)**					
African	2 465	845 (30.18)	662 (23.16) ab	958 (30.89) ab	
Coloured	659	388 (5.85)	155 (22.6) a	116 (1.38) ac	0.00~~0~~1
White/Indian/Asian	373	319 (5.16)	43 (0.85) b	11 (0.27) bc	
**Marital status (n=3 423)**					
Married	1 678	899 (23.43)	375 (12.3) a	404 (12.39) abc	
Living together/civil union	310	112 (3.16)	80 (22.9)	118 (3.01) a	

Never married	757	256 (6.85)	212 (6.25) a	289 (9.13) b	0.00~~0~~1
Widowed	522	196 (5.69)	134 (3.98)	192 (6.12) c	
Separated/divorced	156	61 (1.96)	44 (1.38)	51 (2.07)	
**Educational attainment (n=3 499)**					
Primary	820	243 (6.48)	223 (6.28) a	354 (10.34) ab	
Secondary	1 524	762 (18.27)	390 (12.11) b	372 (11.49) acd	
Tertiary/higher degree	353	280 (8.4)	50 (1.81) abc	23 (0.81) bce	<0.00~~0~~1
No schooling/other	802	268 (8.05)	197 (6.05) c	337 (9.91) de	
**Source of income (n=3 118)**					
Salaries or wages	1 330	774 (22.93)	285 (9.32) ab	271 (9.01) ab	
Social grants/pensions/remittances	1 006	329 (10.69)	266 (9.34) a	411 (14.5) a	
Sale of products and services	138	71 (2.11)	37 (1.46)	30 (1.08) c	<0.00~~0~~1
No income	644	208 (5.54)	174 (5.86) b	262 (8.15) bc	
**Locality (n=3 499)**					
Urban formal	1 838	1 049 (27.85)	410 (12.9) ab	379 (12.77) abc	
Urban informal	464	131 (2.59)	140 (3.11) a	193 (3.98) a	<0.00~~0~~1
Rural formal	359	128 (2.18)	80 (1.19)	151 (2.3) b	
Rural informal	838	245 (8.58)	230 (9.05) b	363 (13.49) c	

~~-~~Multiple 2x2 comparisons with Bonferroni correction: a) p<0.01 between food secure vs. experiencing hunger except for: ‽ Primary vs no schooling, salaries vs products/services; pensions/grants vs product/services and no income; b) p<0.01 between food secure vs. at risk of hunger except for: †Coloured vs White/Indian/Asian; primary vs secondary; primary vs no schooling and secondary vs no schooling; salary vs product/services, pension/grants vs product/services, pension/grants vs no income and product /services vs no income; c) p<0.01 between food secure and at risk of hunger and experiencing hunger for married vs never married and married vs widower; p<0.01 between food secure vs experiencing hunger for married vs living together.
*p<0.001 for age when comparing food secure vs at risk of hunger and food secure vs experiencing hunger (Kruskal-Wallis with Bonferroni correction).

Factors associated with experiencing hunger and being at risk of hunger in all households with and without children

Weighted univariable multinomial logistic regression (**Table 1S**) of all the households exhibits that household heads who were female, of older age, unmarried/widowed/separated vs married were at higher risk of hunger and of experiencing hunger. However, when performing a weighted multivariable multinomial logistic regression (**Fig~~uress~~ 2A & 2B**), age was no longer associated with experiencing hunger. Yet, female-headed households had ~1.53 times increased odds of experiencing hunger as compared to male-headed households (p<0.001). Having a larger household, households with children present, and having a female household head were predictors of experiencing hunger; AOR [95% CI]: 1.06 [1.00 – 1.21], 1.68 [1.12 – 2.53] and 1.53 [1.21 – 1.94], respectively. Having a household head who was either Coloured or White/Indian/Asian as opposed to African ~~was negatively associated with~~conferred lower odds of both experiencing hunger and being at risk of hunger; AOR [95% CI]: 0.29 [0.19 – 0.44] and 0.12 [0.04 – 0.33] for

experiencing hunger, respectively; and, being at risk of hunger; AOR [95% CI]: 0.54 [0.39 – 0.75], 0.22 [0.12 – 0.40], respectively. Furthermore, secondary/tertiary education ~~was negatively associated~~conferred lower odds of ~~with~~ both experiencing hunger; AOR [95% CI]: 0.40 [0.28 – 0.56] and being at risk of hunger; AOR [95% CI]: 0.69 [0.52 – 0.92]. Households relying on pensions/social grants/remittances, or not having any source of income, were ~1.76 to 2.15 times more at risk of hunger and experiencing hunger than those receiving a salary/wage.

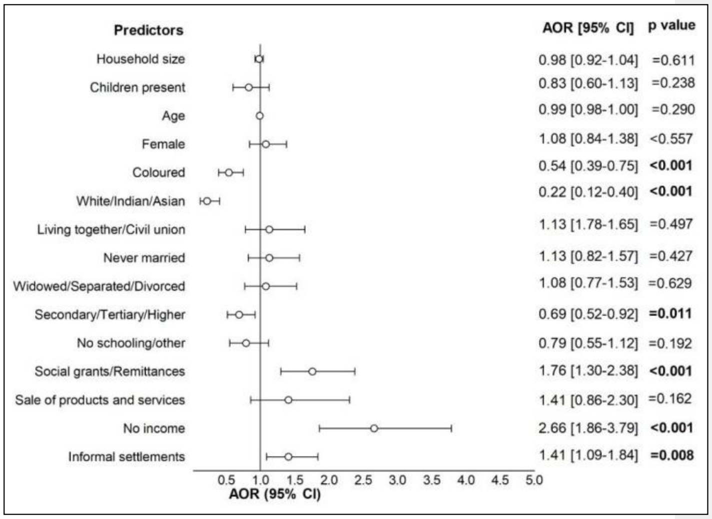

Figure 2A: Factors associated with being at risk of hunger, data presented as adjusted odds ratios (AOR) and 95% Confidence intervals (95% CI)

References categories: *Presence of children (no children present), Gender (male), Race (~~a~~African), Marital status (married), Educational attainment (primary), Source of income (salaries/wages), Locality (formal)*

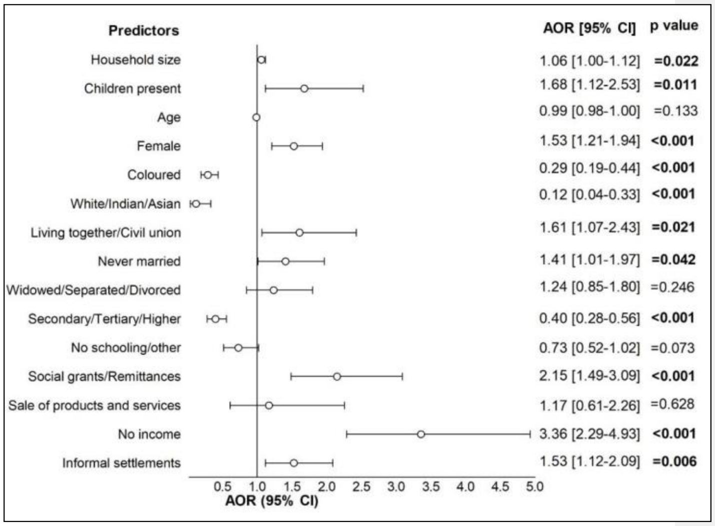

Figure 2B: Factors associated with experiencing hunger, data presented as adjusted odds ratios (AOR) and 95% Confidence intervals (95% CI)

References categories: *Presence of children (no children present), Gender (male), Race (~~a~~African), Marital status (married), Educational attainment (primary), Source of income (salaries/wages), Locality (formal)*

h

Sensitivity analysis

Supplemental Tables 2S and **3S** show the weighted multivariable multinomial logistic regression analysis of factors associated with being at risk of hunger and experiencing hunger against being food secure when the analysis was restricted to households with, and without children. In *households without children* (**Table 2S**), the non-African race i.e. Coloured AOR [95% CI]: 0.44 [0.27 – 0.73] and White/Indian/Asian 0.28 [0.15 – 0.53] race groups and, secondary/tertiary/higher degrees 0.52 [0.30 – 0.88] ~~was negatively associated~~had lower odds of ~~with~~ being at risk of hunger. No income 3.78 [2.01 – 7.08] was ~~a predictor of~~not associated with being at risk of hunger. The female sex AOR [95% CI]: 2.00 [1.15 – 3.50] and no income 5.16 [2.25 – 11.82] conferred increased odds of experiencing hunger while the Coloured 0.15 [0.06 – 0.34] and White/Indian/Asian 0.12 [0.03 – 0.38] race groups and households with heads who attained secondary/tertiary/higher degrees 0.31 [0.14 – 0.70] had lower odds of experiencing hunger.

Formatted: Font: Italic

In *households with children* (**Table 3S**), only the non-African race groups had lower odds of being at risk of hunger, Coloured AOR [95% CI]: 0.64 [0.45 – 0.92] and White/Indian/Asian 0.16 [0.07 – 0.33]. Whereas, pensions/social grants/remittances 1.69 [1.18 – 2.42], no income 2.08 [1.41 – 3.07] and informal location 1.64 [1.20 – 2.24] increased the odds of being at risk of hunger. Again, the non-African race groups, Coloured 0.36 [0.24 – 0.55] and White/Indian/Asian 0.11 [0.03 – 0.36] had lower odds of experiencing hunger. ~~Poignantly, in~~To summarise, in households with children, ~~were leaden by a host of~~ factors that increased the odds of experiencing hunger were~~:~~ ~~namely,~~ household size AOR [95% CI]: 1.06 [1.00 – 1.12], female sex 1.45 [1.07 – 1.95], living together/civil unions 1.72 [1.11 – 2.68], never married 1.46 [1.02 – 2.09], relying on social grants/pensions/remittances and sale of products and services 1.92 [1.31 – 2.79], having no income 2.83 [1.89 – 4.24] and residing in informal settings 1.78 [1.27 – 2.49].

Formatted: Font: Italic

We further illustrate the association due to the presence of children in the household with food security, adjusting for the number of adults in households and the same sociodemographic factors, including main source of income, as in Figures 2A and 2B (**Table 3**). In the univariate analysis, the presence of children confers nearly three times the odds of experiencing hunger OR [95% CI]: 2.97 [2.24 – 3.93]. After adjustment for the number of adults in a household, and the sociodemographic characteristics of the household head including source of income, the presence of children remained independently associated with experiencing hunger AOR [95% CI]: 2.59 [1.78 – 3.77], but not with being at risk of hunger (**Table 3**).

Table 3: Unadjusted and adjusted weighted multinomial logistic regression analyses between the presence of children in a household and being at risk of hunger or experiencing hunger, against food security.

Formatted: Font color: Dark Red

	**Model 1 OR [95% CI] Unadjusted**	**p-value**	**Model 2 AOR [95% CI] Adjusted for the number of adults**	**p-value**	**Model 3 AOR [95% CI] Adjusted for number of adults + sociodemographic characteristics of household head[1]**	**p-value**
**Food security status**						
Food secure	REF		REF		REF	
At risk of hunger	1.06 [0.87 – 1.29]	0.557	1.11 [0.90 – 1.36]	0.303	0.88 [0.69 – 1.12]	0.330

Experience hunger	**2.97 [2.24 – 3.93]**	<0.001	**3.07 [2.29 – 4.11]**	<0.001	**2.59 [1.78 – 3.77]**	<0.001

[1] Variables included were: sex, race, marital status, education, location and source of income

Discussion

To the best of our knowledge, the present study is the first to objectively quantify the burden of ~~childhood~~ food insecurity among children and adolescents in South Africa and draw associations using a large-scale, population-based sample of households with children. No studies in South Africa have disaggregated households with and without children thus possibly underestimating the true extent of childhood food insecurity and limiting direct comparisons to the present study. Indeed, there is an observed ~~discrepancy (~~increase in food insecurity (experiencing hunger~~—~~25.9% to 32.5%) by 6.6%~~)~~ between the prevalence estimate reported for 2008 by Labadarios et al Altman (30) and the estimate generated in the present study. This lack of improvement~~; however, these findings~~ may be accounted for~~,~~ by the enduring socio-political and economic challenges sustained ~~over the years~~since the end of apartheid (28,39,40). Drawing from a cross-sectional, population-based national sample of South African households, we have shown that the prevalence of food insecurity in households with children was nearly 60%. This is ~~.~~ similar to the original SANHANES study where food insecurity among all households was 54% (Shisana, et al 2013). In ~~a multivariable model~~the present study, after adjusting for the number of adults in a household, the sociodemographic characteristics of the household head and the main source of income, the presence of children conferred ~~~1.68~~2.59 increased odds of food insecurity (experiencing hunger) compared to households without children. Having a female vs male household head, having an African household head compared to all other race groups, living in informal settings vs formal settings and having no income compared to relying on salaries or wages increased the household risk of experiencing hunger between 53% and 300%. Secondary/tertiary/higher educational attainment of the household head reduced the risk of experiencing hunger by at least 40% relative to primary education. However, having a household head with no formal schooling was not a predictor for experiencing hunger. In addition, marital status of the household head was not

Commented [LB3]: Check year and reference!! Currently, Reference #28

Commented [LB4]: Attention to these references

independently associated with being at risk of hunger but living together/civil unions and never married increased the odds of experiencing hunger by 61% and 41%, respectively. Relying on social grants, pensions and remittances as the main source of income was not protective against food insecurity.

Gender, presence of children, racial differences, rurality and informality

The majority of households with children in our sample were headed by women, had African household heads, or were living in urban informal or rural informal areas; therefore, the increased vulnerability of households with children may be explained, in part, by the additive effects of these known predictors of food insecurity (41). A mounting body of evidence has corroborated that the presence of children ~~below age 18 years~~ in households is independently associated with food insecurity, beyond sociodemographic characteristics and measures of socioeconomic status (42–45). Our findings also reveal that the presence of children confers more 2.5 times the odds of experiencing hunger beyond sociodemographic characteristics of the household head, including the head's main source of income and educational attainment. Additional research has demonstrated that the sex of the household head is an important factor predicting household vulnerabilities, with the highest prevalence of hunger reported among female-headed households (31,35), a risk that worsens with rural location (31). In contrast to a study by Chakona (36), in which food insecurity increased along the urban-rural continuum of South African towns selected along an agro-ecological gradient, we show that the risk of food insecurity transcends rural-urban differences and becomes more pronounced when stratifying by formal vs informal type of household location. In their large-scale, population-based longitudinal analysis of the Indonesian Family Life Survey, Vaezghasemi (31) further argued that while women, who are at the bottom of the family hierarchy with low social capital, may be conscious of healthy food options but are nonetheless defenseless against household hunger. Given South Africa's colonial and apartheid history, racial differences still persist in the prevalence of food insecurity, with black or African households bearing the greatest burden compared to minority groups (30).

Main source of household income and social grants

Using self-reported main source of income to indicate a household's economic stability, we note that there were no differences in income source between households with and without children. ~~w~~We show that not having any source of income predicts that a household will experience a three-

fold increase in experiencing hunger. Furthermore, similar to Reuters and Wildscut (2010), receiving government social grants and pensions, or remittances, did not alleviate hunger nor the risk of being food insecure for households with and without children. In fact, social grants were not protective in these households, with a 76% increase in being at risk of food insecurity and double the odds for being food insecure. Furthermore, even when controlling for the sociodemographic characteristics of the household head and the main source of income, the risk of experiencing hunger was increased with the presence of children in a household. Other contexts show mixed results for government assistance programmes on food security. Tarasuk (47) have shown that in Canada, household before-tax income adjusted for family size was protective against food insecurity; however, households receiving social assistance were three times more likely to be food insecure. In contrast, Brown & Tarasuk (48) reported reductions in severe food insecurity among households with children subsequent to the roll-out of the Canada Child Benefit (CCB), a country-wide non means-tested cash transfer programme. In Brazil (48), participation in the Bolsa Familia Programme (49,50) and in the United States receiving Supplemental Nutrition Assistance Program (SNAP, formerly the Food Stamp Program) support (51), ~~-~~were not effective in preventing food insecurity ~~(51)~~. In one South African study where social grants did show improvements in household food insecurity and dietary quality Waidler and Devereux (38), the authors note ~~Waidler & Devereux (38) argue~~ that these findings were not accompanied by concomitant improvements in anthropometric indicators of child malnutrition. Furthermore, ~~In their study,~~ while they ~~Waidler & Devereux (38)~~ observed that the Older Persons Grant and remittances had substantial and favorable effects on the dietary diversity score, the Child Support Grant did not [38]. Our findings did not support a similar benefit in households without children. Moreover, when children were present, reliance on social grants, pensions or remittances as the main source of income, was not protective against food insecurity.

Educational attainment

Previous studies have demonstrated strong positive associations between the lowest levels of educational attainment and food insecurity, mitigated by higher levels of educational attainment (30,53). Likewise, we found that improved levels of education (secondary and tertiary educational attainment) reduced the risk of food insecurity by at least 40%; yet, having no formal schooling did not predict food insecurity. As Chakona & Shackleton (53) also found, we posit that household

heads without a formal education may rely on subsistence farming or implement other skills-based practices which act as a *safety net* against food insecurity, thereby insulating the adverse effects of any financial shocks on household food expenditure.

Marital status

The Born in Bradford cohort, a longitudinal study, showed associations between cohabitation status and food insecurity, where pregnant women not living with a partner had a two-fold increased risk of food insecurity (55). Hanson, Sobal, & Frongillo (56) further demonstrated that marital status was associated with food insecurity among men, but not women. In concert with these findings, we found significant associations between the marital status of the household head and risk of experiencing hunger, even after adjustments for sex, where living together/civil unions and never married increased the odds of experiencing hunger by 61% and 41%, respectively. We surmise that unmarried women in extended families may share food costs with other adults co-resident in the household; and, ~~,~~ over generations there is resilience and better coping strategies within the household as the burden of providing food is shared among members.

Contemporary considerations and current context

The present COVID-19 pandemic, occurring alongside household and child food and nutrition insecurity, presents a disconcerting syndemic (9,10) that is capable of reversing ~~the~~ any recent ~~improvements~~ mitigation of hunger at household and individual levels in the country. ~~witnessed between 2002-2017~~Indeed, the COVID-19 pandemic has forced South Africa into an abrupt and prolonged lockdown threatening job and societal security (11,12). Data from the National Income Dynamics Study-Coronavirus Rapid Mobile Survey (NID-CRAMS) (Nwosu, C., Kollamparambil, U., Oyenubi, A. 2021), a four-wave longitudinal survey of adult South Africans, indicate that even with re-opening of the economy, lifting restrictions on movement and returning to work, 37-47% households did not have sufficient money to buy food, with 16-22% stating that they experienced hunger, during the period from May/June 2020 to February/March 2021. Overall, while hunger declined somewhat over this period, the poorest quintile suffered more hunger than before.; Without augmented social supports for households with children and an emphasis on nutrition-sensitive interventions, ~~therefore,~~ it is likely that food insecurity will pervade (13,14) and possibly impede the country from realising its 2030 Sustainable Development Goal of achieving zero hunger (15). As was evident in our sensitivity analysis when deciphering the differential risk of

Commented [LB5]: Use the following references:

1. Nwosu, C., Kollamparambil, U., Oyenubi, A. (2021). Food insecurity and health outcomes during the coronavirus pandemic in South Africa.

ABSTRACT: Given that South Africa experienced significant food insecurity even before the COVID-19 pandemic, it is not surprising that the pandemic and associated control measures would result in even greater food insecurity in the country. This paper seeks to provide additional evidence on the well-known relationship between food insecurity and health. Data came from the National Income Dynamics Study-Coronavirus Rapid Mobile Survey, a four-wave longitudinal survey of adult South Africans. Health was captured using a self-reported indicator of general health status, while food insecurity was measured using information on household hunger, the frequency of hunger, and households running out of money to buy food. We performed descriptive and econometric analyses. Food insecurity has remained stubbornly high even in the face of greater re-opening of the economy, with 37-47% of the population indicating that their households ran out of money to buy food and 16-22% indicating that their households experienced hunger over the May/June 2020 to February/March 2021 period. While hunger generally declined somewhat over this period, the poorest quintile suffered more hunger than before. Moreover, among those whose households experienced hunger, between a quarter and a third struggled with hunger almost everyday or daily. Being in a household that experienced hunger was associated with a 5.5-7.3 percentage point higher probability of worse health compared to not experiencing hunger in May/June 2020-February/March 2021. Compared to being unaffected by hunger, being hungry everyday was associated with a 17-percentage point higher probability of worse health in May/June 2020. These results show the enormity of the hunger problem in South Africa and its adverse effects on welfare. In the face of economic uncertainty and the removal of COVID-19 palliatives like the grant top-ups, policymakers must think of ways to protect the vulnerable from food insecurity. NIDS-CRAM

Commented [LB6]: You should reference Stats-SA (2017) here

food insecurity between households with, and without children, we surmise that these findings may indicate the preponderance of socioeconomic disadvantage among female-headed households. Affirmatively, Broussard (52) found that gender differences in income and education explain the majority of the gender gap in the prevalence of food insecurity.

Limitations

The present study is not without limitations. The SANHANES-1 was conducted in 2011/12. Although never repeated, the data are at least eight years old and may not reflect the current status of food security in South Africa. Nonetheless, the SANHANES provides the most recent and comprehensive dataset ~~-~~on food security at the national and household level using ~~a scale~~the CCHIP index that explores the context and loci within which food insecurity exists. ~~As opposed~~This is in contrast to other surveys which ~~relying on the dimensionless indicator which explores~~ only record the frequency of problems satisfying food needs “in the past 12 months”~~, as is the case in the 2016 Demographic and Health Survey~~ (57). Secondly, a comprehensive assessment of socioeconomic status was beyond the scope of this research. Having considered the primary source of household income and educational status as contributing factors to household economic stability, a more robust measure of socioeconomic status using household assets could add a more granular dimension to the analysis.

Conclusions

~~The data obtained in o~~Our study ~~point to the real possibility that South Africa is still visible on the United Nations hunger map and~~ confirms that South African children face a major challenge of food insecurity that is driven by a number of household risk factors. ~~necessitating multi-sectoral intervention and~~ healthy public policies including the need to focus on the social determinants of ~~health.~~ The untoward effects of food insecurity on child and adolescent~~hood~~ health are not evanescent ~~but~~ and have far-reaching adverse health outcomes. Therefore, this is a clarion call to bolster multi-sectoral interventions and healthy public policies, with a strong focus on the social determinants of health and poverty eradication strategies that ~~strategies for poverty eradication, particularly~~ target~~ing~~ households with greater ~~social~~ vulnerability such as those with children. ~~necessitating multi-sectoral intervention and healthy public policies including the need to focus on the social determinants of health.~~ There is an urgent need for guaranteeing the constitutional *right*

to food and basic nutrition for children especially during the Covid-19 era, a time of heightened need.

Supporting information files

Figure 1S: A diagram illustrating the database management, organisation and integration of data extracted from the four household and individual level datasets of the SANHANES-1

Table 1S: Weighted univariable multinomial logistic regression analysis of factors associated with being at risk of hunger and experiencing hunger against being food secure

Table 2S: Weighted multivariable multinomial logistic regression analysis of factors associated with being at risk of hunger and experiencing hunger against being food secure in households without children

Table 3S: Weighted multivariable multinomial logistic regression analysis of factors associated with being at risk of hunger and experiencing hunger against being food secure in households with children

Excel file 1S:

Acknowledgements

The authors acknowledge Dr Innocent Maposa for his assistance with data analysis.

References

Commented [LB7]: References could not be updated in this copy; please see final manuscript clean copy with full and corrected reference list

Field Code Changed

1. OHCHR O of the UNHC for HR. The Right to adequate food Fact Sheet No. 34. Vol. 41987. 2006. 1–49 p.
2. Skoet J, Stamoulis KG, Food and Agriculture Organization of the United Nations. Economic and Social Department. The state of food insecurity in the world, 2006 : eradicating world hunger : taking stock ten years after the World Food Summit. 2006. 40 p.
3. Neuman N, Eli K, Nowicka P. Feeding the extended family: gender, generation, and socioeconomic disadvantage in food provision to children. Food, Cult Soc [Internet]. 2019;22(1):45–62. Available from: https://doi.org/10.1080/15528014.2018.1547066
4. Fraval S, Hammond J, Bogard JR, Ng'endo M, van Etten J, Herrero M, et al. Food Access Deficiencies in Sub-saharan Africa: Prevalence and Implications for Agricultural

Interventions. Front Sustain Food Syst. 2019;3(November).

5. World Bank. Ending Poverty, Investing in Opportunity. World Bank Gr. 2018;319.

6. Stats-SA. Towards measuring food security in South Africa: An examination of hunger and food inadequacy [Internet]. 2019. 26 p. Available from: www.statssa.gov.za

7. Nord M. Food Insecurity in Households With Children: Prevalence , Severity , and Household Characteristics. USDA Econ Res Rep [Internet]. 2009;(56):1–43. Available from: http://files.eric.ed.gov/fulltext/ED508211.pdf

8. Ngema PZ, Sibanda M, Musemwa L. Household food security status and its determinants in Maphumulo local municipality, South Africa. Sustain. 2018;10(9):1–23.

9. Pérez-Escamilla R, Cunningham K, Moran VH. COVID-19 and maternal and child food and nutrition insecurity: a complex syndemic. Matern Child Nutr. 2020 Jul;16(3):8–11. doi: 10.1111/mcn.13036.

10. Watanabe M, Risi R, Tuccinardi D, Baquero CJ, Manfrini S, Gnessi L. Obesity and SARS-CoV-2: a population to safeguard. Diabetes Metab Res Rev. 2020;(April):15–6.

11. International Labour Organization. ILO Monitor : COVID-19 and the world of work. Third edition U [Internet]. 2020. 1–23 p. Available from: https://www.ilo.org/wcmsp5/groups/public/---dgreports/---dcomm/documents/briefingnote/wcms_743146.pdf

12. Labadarios D, Mchiza Z, Steyn NP, Gericke G, Maunder E, Davids Y, et al. WHO | Food security in South Africa: a review of national surveys. WHO [Internet]. 2011 Dec 1 [cited 2018 Apr 29];89(12):891–9. Available from: http://www.who.int/bulletin/volumes/89/12/11-089243.pdf

13. Pedroso J, Buccini G, Venancio SI, Pérez-Escamilla R, Gubert MB. Maternal mental health modifies the association of food insecurity and early child development. Matern Child Nutr. 2020;(March):1–12.

14. Roberton T, Carter ED, Chou VB, Stegmuller AR, Jackson BD, Tam Y, et al. Early estimates of the indirect effects of the COVID-19 pandemic on maternal and child mortality in low-income and middle-income countries: a modelling study. Lancet Glob Heal [Internet]. 2020;(20):1–8. Available from: http://dx.doi.org/10.1016/S2214-109X(20)30229-1

15. Pérez-Escamilla R. Food security and the 2015-2030 sustainable development goals: From human to planetary health. Curr Dev Nutr. 2017;1(7):1–8.

16. Webb P, Stordalen GA, Singh S, Wijesinha-Bettoni R, Shetty P, Lartey A. Hunger and malnutrition in the 21st century. BMJ. 2018;361:1–5.

17. Hall K, Mokomane Z. The shape of children's families and households: A demographic overview. South African Child Gauge Child Fam State [Internet]. 2018;32–45. Available from: http://www.ci.uct.ac.za/ci/child-gauge/2018

18. Sung-King M, Lake L, Sanders D, Hendricks M. South African Child Gauge 2019: Child

and adolescent health leave no one behind. South African Child Gauge. 2019. 30–62 p.

19. Pérez-Escamilla R, de Toledo Vianna RP. Food insecurity and the behavioral and intellectual development of children: a review of the evidence. J Appl Res Child. 2012;3(1):18.

20. Seligman HK, Laraia B a, Kushel MB. Food Insecurity Is Associated with Chronic Disease among Low-Income. J Nutr. 2009;140:304–10.

21. Kirkpatrick SI, McIntyre L, Potestio ML. Child hunger and long-term adverse consequences for health. Arch Pediatr Adolesc Med. 2010;164(8):754–62.

22. Ke J, Ford-Jones EL. Food insecurity and hunger: A review of the effects on children's health and behaviour. Paediatr Child Heal. 2015;20(2):89–91.

23. Sparén P, Vågerö D, Shestov DB, Plavinskaja S, Parfenova N, Hoptiar V, et al. Long term mortality after severe starvation during the siege of Leningrad : prospective cohort study. Br Med J. 2003;(December):1–5.

24. Lumey LH, Stein AD, Kahn HS, Bruin KMVDP, Blauw GJ, Zybert PA, et al. Cohort Profile : The Dutch Hunger Winter Families Study. Int J Epidemiol. 2007;36(June):1196–204.

25. Neyroud P. Literature + IDEAS. Curr Probl Pediatr Adolesc Health Care. 2015;(310).

26. Bjerregaard LG, Jensen BW, Ängquist L, Osler M, Sørensen TIA, Baker JL. Change in overweight from childhood to early adulthood and risk of type 2 diabetes. N Engl J Med. 2018;378(14):1302–12.

27. Labadarios D, Steyn NP, Maunder E, Macintryre U, Gericke G, Swart R, et al. The National Food Consumption Survey (NFCS): South Africa , 1999. Public Health Nutr. 2005;8(5):533–43.

28. Altman M, Jacobs P, Aliber M, Baiphethi M, Davids YD, Ladzani R, et al. Food security in South Africa by Project Team. Access. 2009.

29. Vaezghasemi M, Öhman A, Eriksson M, Hakimi M, Weinehall L, Kusnanto H, et al. The Effect of Gender and Social Capital on the Dual Burden of Malnutrition: A Multilevel Study in Indonesia. Noor AM, editor. PLoS One [Internet]. 2014 Aug 25 [cited 2018 May 29];9(8):e103849. Available from: http://dx.plos.org/10.1371/journal.pone.0103849

30. Mutisya M, Ngware MW, Kabiru CW, Kandala N bakwin. The effect of education on household food security in two informal urban settlements in Kenya: a longitudinal analysis. Food Secur [Internet]. 2016;8(4):743–56. Available from: http://dx.doi.org/10.1007/s12571-016-0589-3

31. Akadiri S Saint, Nwaka ID, Jenkin GP. Are Female-Headed Households Less Food Secure? Evidence from Nigeria and Ethiopia. AFEA Sess Soc Sci Assoc Annu Conf Philadelphia 2018. 2018;.

32. Coates, J., Swindale, A., Bilinsky P. HFIAS for Measurement of Food Access Indicator Guide. J Chem Inf Model. 2013;53(9):1689–99.

33. Segall-Corrêa AM, Marin-León L, Melgar-Quiñonez H, Pérez-Escamilla R. Refinement of the Brazilian household food insecurity measurement scale: Recommendation for a 14-item EBIA. Rev Nutr. 2014;27(2):241–51.

34. Chakona G, Shackleton CM. Household Food Insecurity along an Agro-Ecological Gradient Influences Children ' s Nutritional Status in South Africa. Front Nutr. 2018;4(January):1–13.

35. Ruiters M, Wildschutt A. Food insecurity in South Africa: Where does gender matter? Agenda Empower women Gend equity. 2010;24(86):8–24.

36. Waidler J, Devereux S. Social grants, remittances, and food security: does the source of income matter? Food Secur. 2019;11(3):679–702.

37. de Onis M, Garza C, Onyango AW, Rolland-Cachera M-F. Les standards de croissance de l'Organisation mondiale de la santé pour les nourrissons et les jeunes enfants. Arch Pédiatrie. 2009;16(1):47–53.

38. Swindale A, Bilinsky P. Development of a Universally Applicable Household Food Insecurity Measurement Tool: Process, Current Status, and Outstanding Issues. J Nutr. 2006;136(5):1449S-1452S.

39. Drimie S, Ruysenaar S. The integrated food security strategy of South Africa: An institutional analysis. Agrekon. 2010;49(3):316–37.

40. de Oliveira KHD, de Almeida GM, Gubert MB, Moura AS, Spaniol AM, Hernandez DC, et al. Household food insecurity and early childhood development: Systematic review and meta-analysis. Matern Child Nutr. 2020;16(3):1–27.

41. Farrell P, Thow AM, Abimbola S, Faruqui N, Negin J. How food insecurity could lead to obesity in LMICs. Health Promot Int. 2018;33(5):812–26.

42. Chinnakali P, Upadhyay RP, Shokeen D, Singh K, Kaur M, Singh AK, et al. Prevalence of Household-level Food Insecurity and Its Determinants in an Urban Resettlement Colony in North India. J Heal Popul Nutr. 2014;32(2):227–36.

43. Mortazavi Z, Dorosty AR, Eshraghian MR, Ghaffari M, Ansari-moghaddam A, Mohammadi M. Household Food Insecurity in Southeastern Iran : Severity and Related Factors. Int J Food Sci. 2017;2017(7):1–7.

44. Tantu AT, Gamebo TD, Sheno BK. Household food insecurity and associated factors among households in Wolaita Sodo town , 2015. Agric Food Secur. 2017;1–8.

45. Sisha TA. Household level food insecurity assessment : Evidence from panel data , Ethiopia. Sci African. 2020;7:1–9.

46. Tarasuk V, Fafard St-Germain AA, Mitchell A. Geographic and socio-demographic predictors of household food insecurity in Canada, 2011-12. BMC Public Health. 2019;19(1):1–12.

47. Brown EM, Tarasuk V. Money speaks : Reductions in severe food insecurity follow the Canada Child Benefit. Prev Med (Baltim) [Internet]. 2019;129(October):105876.

Available from: https://doi.org/10.1016/j.ypmed.2019.105876

48. de Sousa LRM, Segall-Corrêa AM, Ville A Saint, Melgar-Quiñonez H. Food security status in times of financial and political crisis in Brazil. Cad Saude Publica. 2019;35(7):1–13.

49. Costa NS, Santos MO, Carvalho CPO, Assunção ML, Ferreira HS. Prevalence and Factors Associated with Food Insecurity in the Context of the Economic Crisis in Brazil. Curr Dev Nutr. 2017;1(10):e000869.

50. Paranaense U, Londrina UE De, Campinas UE De. Insegurança alimentar entre beneficiários. Rev Nutr. 2012;25(2):177–89.

51. Cook JT, Frank D a, Levenson SM, Neault NB, Heeren TC, Black MM, et al. Food assistance and the well-being of low-income families child food insecurity increases risks posed by household. J Nutr. 2006;136:1073–6.

52. Broussard NH. What explains gender differences in food insecurity? Food Policy [Internet]. 2019;83(January):180–94. Available from: https://doi.org/10.1016/j.foodpol.2019.01.003

53. Stephen Devereux, Tessa Hochfeld, Abdulrazak Karriem, Clement Mensah, Matseliso Morahanye, Thabang Msimango, Agnes Mukubonda, Sigamoney Naicker, Grace Nkomo DS and MS. School Feeding in South Africa:What we know, what we don’t know, what we need to know, what we need to do Food Security SA Working Paper Series No. 004. DST-NRF Centre of Excellence in Food Security, South Africa. 2018.

54. Chakona G, Shackleton CM. Food insecurity in South Africa : To what extent can social grants and consumption of wild foods eradicate hunger ? World Dev Perspect [Internet]. 2019;13(October 2017):87–94. Available from: https://doi.org/10.1016/j.wdp.2019.02.001

55. Power M, Uphoff EP, Stewart-knox B, Small N, Doherty B, Pickett KE. Food insecurity and socio-demographic characteristics in two UK ethnic groups : an analysis of women in the Born in Bradford cohort. J Public Heal. 2017;40(1):32–40.

56. Hanson KL, Sobal J, Frongillo EA. Gender and Marital Status Clarify Associations between Food Insecurity and Body Weight 1 , 2. J Nutr. 2007;(December 2006):3–8.

57. South Africa. Department of Health, Statistics South Africa, South African Medical Research Council, Icf International (Firm. South Africa demographic and health survey, 2016. Pretoria: National Department of Health, January; 2019.

Supporting information

S1 Fig. Database management, organisation and integration of data extracted from the four household and individual level datasets of the SANHANES-1

Formatted: Font: 16 pt, Not Bold

Formatted: Font: Not Bold

Formatted: Font: Not Bold

S1 Table. Weighted univariable multinomial logistic regression analysis of factors associated with being at risk of hunger and experiencing hunger against being food secure

S2 Table. Weighted multivariable multinomial logistic regression analysis of factors associated with being at risk of hunger and experiencing hunger against being food secure in households without children

S3 Table. Weighted multivariable multinomial logistic regression analysis of factors associated with being at risk of hunger and experiencing hunger against being food secure in households with children

S1 Database; Final analytic sample of households with food security categories

Formatted: Font: Not Bold

Enquiries: Mr. Siluleko Mkhize

Contact number: +27 84 635 7216

Email: 843928@students.wits.ac.za

Date: 24 June 2021

Academic editor: Rubeena Zakar, PhD

PLOS ONE

1265 Battery Street
Floor 2
San Francisco, CA 94111
United States
Phone: +1 (415) 624-1200 | Fax: +1 (415) 546-4090

Response to reviewers' comments

Manuscript: PONE-S-20-49924

Title: Childhood food insecurity in South Africa: A household-level analysis of hunger

Queries	Responses
**Comments from academic editor Rubeena Zakar, PhD**	
1. Please ensure that your manuscript meets PLOS ONE's style requirements, including those for file naming.	Our manuscript now meets PLOS ONE's style requirements. The following changes were made directly in the final manuscript:<br>1. Title headings are now in the correct font size<br>2. Figures were re-labelled in the correct format<br>3. Acknowledgement section was moved to the correct place<br>4. Vancouver referencing was updated and numbers placed in square brackets in the text<br>5. Where necessary, files were re-named
2. Please include your tables as part of your main manuscript and remove the individual files. Please note that supplementary tables (should remain/ be uploaded) as separate "supporting information" files.	Tables were moved into the main body of the manuscript (Results section). S1 Fig, S1, S2 and S3 Tables and S1 Database are uploaded as separate "supporting information" files.
3. We note that you have indicated that data from this study are available upon request. PLOS only allows data to be available upon request if there are legal or ethical restrictions on sharing data publicly.	As no legal or ethical restrictions apply, we have included the de-identified anonymised data set used for this study as a "supporting information" file entitled S1 Database. The data availability statement should now read:

a) If there are ethical or legal restrictions on sharing a de-identified data set, please explain them in detail (e.g., data contain potentially identifying or sensitive patient information) and who has imposed them (e.g., an ethics committee). Please also provide contact information for a data access committee, ethics committee, or other institutional body to which data requests may be sent.<br><br>b) If there are no restrictions, please upload the minimal anonymized data set necessary to replicate your study findings as either Supporting Information files or to a stable, public repository and provide us with the relevant URLs, DOIs, or accession numbers. Please see http://www.bmj.com/content/340/bmj.c181.long for guidelines on how to de-identify and prepare clinical data for publication.	*The final de-identified anonymised data set used in this study is provided as a "supporting information" file in this manuscript (S1 Database). A data sharing agreement was signed with the Human Sciences Research Council (HSRC), the curator of the data collected in the 2012 SANHANES survey. Adult SANHANES data are available through registered access from the HSRC data repository at: http://curation.hsrc.ac.za/Datasets-XKAHAA.phtml. Child data are under third party rights and can be accessed upon reasonable request by contacting datahelp@hsrc.ac.za*<br><br>In addition, S1 Fig, Database Management, Organisation and Integration, has been revised with additional explanation about the comprehensive data management strategy that was employed to derive the final analytic sample and explanatory text added, to facilitate access to the data included for this study.
4. Thank you for stating the following in the Acknowledgments Section of your manuscript:<br><br>*[SM acknowledges the South African National Research Foundation for financial support of his postgraduate studies which enabled him to pursue this research.]*<br><br>We note that you have provided funding information that is not currently declared in your Funding Statement. However, funding information should not appear in the Acknowledgments section or other areas of your manuscript. We will only publish funding information present in the Funding Statement section of the online submission form.<br><br>Please remove any funding-related text from the manuscript and let us know how you would like to update your Funding Statement. Currently, your Funding Statement reads as follows:<br><br>*[The author(s) received no specific funding for this work]*<br><br>Please include your amended statements within your cover letter; we will change the online submission form on your behalf.	Funding information has been removed from the acknowledgements section of the manuscript.<br><br>Thank you for updating the online submission form to the amended Funding Statement below:<br><br>*SM received funding from the South African National Research Foundation (NRF): Freestanding, Innovation and Scarce Skills Development Fund Masters and Doctoral Scholarships (Reference; SFH170525233408 and MND190719457401) to support his postgraduate studies which enabled him to pursue this research; as well, supplemental funding was sourced from the Carnegie Corporation of New York.*
5. Please include captions for your Supporting Information files at the end of your manuscript, and update any in-text citations to match accordingly.	Captions for all "Supporting Information" files have been added at the end of the manuscript (immediately after the References section). In-text citations were revised accordingly.
**Reviewers' comments to author**	
**Reviewer #1:**<br><br>Since the previous research studies related to food insecurity lack a specific focus on households with	We appreciate the reviewer's positive assessment of the manuscript, which require no changes to the manuscript.

children, so I found the study worth reading. A comparative analysis of different household characteristics between households with and without children is made. Various descriptive and inferential statistical methods have been used by the authors to analyze the data. The findings of the study are quite interesting and believable. The language and draft of the paper is also excellent.	
**Reviewer #2:**	
Generally, I found this manuscript to be well written. However, the relevance to current circumstances is questionable and the findings are not adequately explored.<br><br>I provide specific comments below if you choose to make revisions.	We appreciate the reviewer's substantive critique of the manuscript and have addressed in detail each of the specific comments below.
1. The SANHANES-1 dataset has been used for many publications in the academic literature. The introduction needs to better summarise previous research and identify the gap in the literature that this study seeks to fill.<br><br>Please reference Shisana and colleagues 2013 report at the very least. An early blog post by Parker in 2013 on the data may also be relevant.	We have modified the text to better summarise previous research, including the SANHANES-1, and clarify the research gap.<br><br>In the final manuscript, see the Introduction section, Lines 107 – 113; 126 – 134.<br><br>References were added, including Shisana et al. [11] The Reference list has been updated to reflect these additions.
2. Lines 92 – 20 years of age seems like an arbitrary cut-off for the definition of a child.	We justify the age selection in the Methods section, Lines 190 – 193.
3. Lines 96 & 419 refers to improvements between 2002 and 2017. Source and further information is needed.	Controversial evidence in the literature, and no use of comparable measurements (eg: the CCHIP Index), resulted in us removing these statements.
4. Line 138 introduces the concept of nutrition-sensitive interventions without any definition, background, or references. Consider adding a paragraph summarising nutrition-sensitive intervention generally and in South Africa and how sociodemographic correlates can inform the design of these interventions to improve childhood health.	The comment has been addressed in Lines 146 – 153, and the notion of nutrition-sensitive interventions has been picked up in the Discussion as well.
5. Lines 140-140 The sentence spanning these lines is repeating information from earlier in the introduction. Rephrase.	Sentence removed.
6. Lines 198-199 The sentence spanning these lines is superfluous. This is rudimentary data manipulation and is expressed in the previous sentence.	Sentence removed.

7. Lines 273-275 This is unclear. What were the differences?	We have re-worded the sentence for clarity and directionality (Lines 303 – 306).<br>*A further analysis of the collapsed locality categories (formal vs informal) yielded the following: 37.2% of households with children were informally located; and, of these, only 28.9% were food secure. In contrast, of the 62.9% of households with children located in formal areas, 53.6% were food secure (p<0.001).*
8. Lines 287, 290, 305, 312 Identifying factors as being directly 'protective for experiencing hunger' is misleading. Many of these factors are likely causally related to asset endowment and/or income. They are not protective of but negatively related to. However, this term is appropriate when discussing social grants – that are designed to be protective against extreme poverty and food insecurity.	Sentences have been re-worded to "had lower odds of" instead of "protective for" experiencing hunger in the Results and Discussion sections. The language in the Abstract has also been revised.<br>The relationship to social grants, pensions and remittances retains the wording "protective against" experiencing hunger.<br>All of these changes are visible in the Revised Manuscript with track changes document.
9. Lines 312-313 The sentence spanning these lines seems more emotive than the rest of the results section.	The sentence has been revised according to scientific language.
10. A theme throughout the manuscript is this purported influence of having children on food insecurity. This appears twice in the introduction, is expressed as a poignant point in the results section and in discussion it is suggested that there is a mounting body of evidence on the topic. The evidence as presented in this manuscript is weak in supporting the assertion "children below age 18 years in households is independently associated with food insecurity, beyond sociodemographic characteristics and measures of socioeconomic status".	We have addressed this point through the following changes to the manuscript:<br>1. We present new evidence in the Results section (Table 3 and text), corresponding to Lines 376 – 387, to strengthen the evidence for the presence of children in households and their impact on food insecurity.<br>2. This has been carried forward into the Discussion, Lines 402 – 405.
11. The most interesting finding in this manuscript is that "social grants and pensions, or remittances, did not alleviate hunger or the risk of being food insecure." This needs to be analysed and discussed further.	We addressed this comment as follows:<br>1. We amended Table 1 to include Source of Income, in order to compare households with and without children (Lines 287 – 293).<br>2. We did further analysis (Table 3) and added to the Discussion (Main source of household income and social grants). The new section now reads (Lines 445 – 449):<br>*"In fact, social grants were not protective in these households, with a 76% increase in being at risk of food insecurity and double the odds for being food insecure. Furthermore, even when controlling for the sociodemographic characteristics of the household head and the main source of income, the risk of experiencing hunger was increased with the presence of children in a household."*<br>Together with Lines 461 – 465:<br>*"Furthermore, while they observed that the Older Persons Grant and remittances had substantial and favourable effects on the dietary diversity score, the Child Support Grant did not [26]. Our findings did not support a similar benefit for households without children. Moreover, when children were present, reliance on*

	social grants, pensions or remittances as the main source of income, was not protective against food insecurity."
12. The link between this manuscript and the COVID-19 pandemic is tenuous. The authors have done very little to link the dated dataset to present day.	The text has been updated in the Discussion to address COVID-19 considerations and food insecurity: Lines 488 – 498.
13. Inconsistency in p value presentation.	We amended presentation of p values to three decimal points, and added p values to the Odds Ratios throughout the manuscript.
14. Original sources need to be cited.	References have been amended to cite original sources.

We submit these changes together with the revised manuscript and supplemental files.

Kindly note that I am an MSc student at Wits University; and, while I appreciate the title of "Dr", it is a degree that I have not yet earned.

(Mr.) Siluleko A. Mkhize
