# Supplementary material for: Child and adolescent food insecurity in South Africa: A household-level analysis of hunger
Source: PLoS One. 2022 Dec 28;17(12):e0278191. doi: 10.1371/journal.pone.0278191 (PMC9797094; doi:10.1371/journal.pone.0278191)
Supplement: Attachment — Submitted filename: PONE-D-20-39991_R3_EA.pdf [file pone.0278191.s009.pdf]

PLOS ONE

Childhood food insecurity in South Africa: A household-level analysis of hunger

--Manuscript Draft--

**Manuscript Number:**	PONE-D-20-39991R3
**Article Type:**	Research Article
**Full Title:**	Childhood food insecurity in South Africa: A household-level analysis of hunger
**Short Title:**	Childhood food insecurity
**Corresponding Author:**	Laurel Baldwin-Ragaven, MDCM<br>University of the Witwatersrand<br>Johannesburg, Gauteng SOUTH AFRICA
**Keywords:**	Keywords: children; adolescents; household; food insecurity; hunger; CCHIP index; South Africa; SANHANES
**Abstract:**	Abstract<br><br>Food insecurity impacts childhood nutritional status, physical and cognitive development, and increases lifetime risk for chronic disease. Previous South African studies have examined hunger at the sub-national level without a specific focus on children and adolescents. This study determines the national prevalence of childhood food insecurity, from birth to adolescence, and identifies factors associated with hunger within the household. Individual and household-level data were extracted from the South African National Health and Nutrition Examination Survey (SANHANES-1). Prevalence of food insecurity was assessed using the Community Childhood Hunger Identification Project (CCHIP) index. Multinomial logistic regression analyses were conducted on all households (with and without children) to determine the predictors of food insecurity, with additional analyses adjusting for child dependency and sociodemographic characteristics of household heads in households with children. Of 5 098 households surveyed, 68.6% had children and adolescents present (0-19 years). Of these households, 32.5% [95% CI (29.5 – 35.7)] were experiencing hunger and 26.3% [95% CI (23.9 – 28.8)] were at risk of hunger. Among all the households, significant associations for experiencing hunger were the presence of children and adolescents: AOR [95% CI]: 1.68 [1.12 – 2.53]; being female-headed: AOR [95% CI]: 1.53 [1.21 – 1.94] and informally-located; AOR [95% CI]: 1.61 [1.07 – 2.43]. Of the racial groups, having a non-African household head, Coloured: AOR [95% CI]: 0.29 [0.19 – 0.44] and White/Indian/Asian: 0.12 [0.04 – 0.33] conferred lower odds of experiencing hunger; and, the household head having secondary/tertiary education conferred lower odds of experiencing hunger; AOR [95% CI]: 0.40 [0.28 – 0.56] as well as being at risk of hunger; AOR [95% CI]: 0.69 [0.52 – 0.92]. Receiving social grants, pensions, or remittances more than doubled the odds of experiencing hunger; AOR [95% CI]: 2.15 [1.49 – 3.09]. After adjusting for child dependency in households with children, having at least one older child (age 15-19 years old) did not change the odds of food insecurity. In summary, only 41% of South African households with children and adolescents were food secure. The associations between household head sociodemographics, household location and size on household food insecurity indicate a need for multi-sectoral interventions to bolster sustainable food systems for households with children and adolescents and to improve public protections for female-headed, African-headed and informally-located households dependent on social grants.
**Order of Authors:**	Siluleko Advice Mkhize, MSc Med
	Elena Libhaber
	Ronel Sewpaul
	Priscilla Reddy
	Laurel Baldwin-Ragaven
**Response to Reviewers:**	Kindly see attached "Response to Reviewers" file.
**Additional Information:**	

Powered by Editorial Manager® and ProduXion Manager® from Aries Systems Corporation

Question	Response
**Financial Disclosure**<br><br>Enter a financial disclosure statement that describes the sources of funding for the work included in this submission. Review the submission guidelines for detailed requirements. View published research articles from *PLOS ONE* for specific examples.<br><br>This statement is required for submission and **will appear in the published article** if the submission is accepted. Please make sure it is accurate.<br><br>**Unfunded studies**<br>Enter: *The author(s) received no specific funding for this work.*<br><br>**Funded studies**<br>Enter a statement with the following details:<br>• Initials of the authors who received each award<br>• Grant numbers awarded to each author<br>• The full name of each funder<br>• URL of each funder website<br>• Did the sponsors or funders play any role in the study design, data collection and analysis, decision to publish, or preparation of the manuscript?<br>• **NO** - Include this sentence at the end of your statement: *The funders had no role in study design, data collection and analysis, decision to publish, or preparation of the manuscript.*<br>• **YES** - Specify the role(s) played.<br><br>* typeset	SM received funding from the South African National Research Foundation (NRF): Freestanding, Innovation and Scarce Skills Development Fund Masters and Doctoral Scholarships (Reference; SFH170525233408 and MND190719457401) to support his postgraduate studies which enabled him to pursue this research; as well, supplemental funding was sourced from the Carnegie Corporation of New York.
**Competing Interests**<br><br>Use the instructions below to enter a competing interest statement for this submission. On behalf of all authors, disclose any competing interests that could be perceived to bias this work—acknowledging all financial support and any other relevant financial or non-financial competing interests.<br><br>This statement **will appear in the published article** if the submission is	The authors have declared that no competing interests exist

accepted. Please make sure it is accurate. View published research articles from *PLOS ONE* for specific examples.<br><br>**NO authors have competing interests**<br><br>Enter: *The authors have declared that no competing interests exist.*<br><br>**Authors with competing interests**<br><br>Enter competing interest details beginning with this statement:<br><br>*I have read the journal's policy and the authors of this manuscript have the following competing interests: [insert competing interests here]*<br><br>* typeset	
**Ethics Statement**<br><br>Enter an ethics statement for this submission. This statement is required if the study involved:<br><br>• Human participants<br>• Human specimens or tissue<br>• Vertebrate animals or cephalopods<br>• Vertebrate embryos or tissues<br>• Field research<br><br>Write "N/A" if the submission does not require an ethics statement.<br><br>General guidance is provided below. Consult the submission guidelines for detailed instructions. **Make sure that all information entered here is included in the Methods section of the manuscript.**	The original SANHANES-1 (2011/12) received ethics approval from the Research Ethics Committee (REC) of the HSRC (REC 6/16/11/11). The present study secondary analysis received ethics approval from the Human Research Ethics Committee (HREC) (Medical) of the University of the Witwatersrand, Johannesburg, South Africa (Clearance certificate number: M180775).

**Format for specific study types**<br><br>**Human Subject Research (involving human participants and/or tissue)**<br>• Give the name of the institutional review board or ethics committee that approved the study<br>• Include the approval number and/or a statement indicating approval of this research<br>• Indicate the form of consent obtained (written/oral) or the reason that consent was not obtained (e.g. the data were analyzed anonymously)<br><br>**Animal Research (involving vertebrate animals, embryos or tissues)**<br>• Provide the name of the Institutional Animal Care and Use Committee (IACUC) or other relevant ethics board that reviewed the study protocol, and indicate whether they approved this research or granted a formal waiver of ethical approval<br>• Include an approval number if one was obtained<br>• If the study involved *non-human primates*, add *additional details* about animal welfare and steps taken to ameliorate suffering<br>• If anesthesia, euthanasia, or any kind of animal sacrifice is part of the study, include briefly which substances and/or methods were applied<br><br>**Field Research**<br><br>Include the following details if this study involves the collection of plant, animal, or other materials from a natural setting:<br>• Field permit number<br>• Name of the institution or relevant body that granted permission	
**Data Availability**<br><br>Authors are required to make all data underlying the findings described fully available, without restriction, and from the time of publication. PLOS allows rare exceptions to address legal and ethical concerns. See the PLOS Data Policy and FAQ for detailed information.	Yes - all data are fully available without restriction

A Data Availability Statement describing where the data can be found is required at submission. Your answers to this question constitute the Data Availability Statement and **will be published in the article**, if accepted.<br><br>**Important:** Stating 'data available on request from the author' is not sufficient. If your data are only available upon request, select 'No' for the first question and explain your exceptional situation in the text box.<br><br>Do the authors confirm that all data underlying the findings described in their manuscript are fully available without restriction?	
**Describe where the data may be found in full sentences. If you are copying our sample text, replace any instances of XXX with the appropriate details.**<br><br>• If the data are **held or will be held in a public repository**, include URLs, accession numbers or DOIs. If this information will only be available after acceptance, indicate this by ticking the box below. For example: *All XXX files are available from the XXX database (accession number(s) XXX, XXX.*).<br>• If the data are all contained **within the manuscript and/or Supporting Information files**, enter the following: *All relevant data are within the manuscript and its Supporting Information files.*<br>• If neither of these applies but you are able to provide **details of access elsewhere**, with or without limitations, please do so. For example:<br><br>*Data cannot be shared publicly because of [XXX]. Data are available from the XXX Institutional Data Access / Ethics Committee (contact via XXX) for researchers who meet the criteria for access to confidential data.*<br><br>*The data underlying the results presented in the study are available from (include the name of the third party*	The final de-identified anonymised data set used in this study is provided as a "supporting information" file in this manuscript (S1 Database). A data sharing agreement was signed with the Human Sciences Research Council (HSRC), the curator of the data collected in the 2012 SANHANES survey. Adult SANHANES data are available through registered access from the HSRC data repository at: http://curation.hsrc.ac.za/Datasets-XKAHAA.phtml. Child data are under third party rights and can be accessed upon reasonable request by contacting datahelp@hsrc.ac.za

and contact information or URL).<br>• This text is appropriate if the data are owned by a third party and authors do not have permission to share the data.<br><br>* typeset	
Additional data availability information:	

Childhood food insecurity in South Africa:

A household-level analysis of hunger

Siluleko A. Mkhize[1], Elena Libhaber[2], Ronel Sewpaul[3], Priscilla Reddy[3,4] and Laurel Baldwin-Ragaven[1*]

[1]Department of Family Medicine and Primary Care, School of Clinical Medicine, Faculty of Health Sciences, University of the Witwatersrand, Johannesburg, South Africa

[2]School of Clinical Medicine and Health Sciences Research Office, Faculty of Health Sciences, University of the Witwatersrand, Johannesburg, South Africa

[3]Health and Wellbeing, Human and Social Capabilities Division, Human Sciences Research Council, Cape Town, South Africa

[4]Faculty of Health Sciences, Nelson Mandela University, Port Elizabeth, South Africa

***Corresponding author**
E-mail: laurel.baldwin-ragaven@wits.ac.za (LBR)

Contributions

SM, LBR and EL conceptualised the study and its design. SM, EL, LBR and RS conducted and interpreted the analyses. SM wrote the first draft of the manuscript; and, EL LBR and RS worked on subsequent iterations of the manuscript. RS and PR critically revised the manuscript for important intellectual content. All authors approved the final manuscript and take primary responsibility for its contents. The contents of the manuscript are solely the responsibility of the

authors and do not necessarily represent the official views of the HSRC or the University of the Witwatersrand, Johannesburg, South Africa.

ORCID

Siluleko Mkhize https://orcid.org/ 0000-0002-8351-6891

Elena Libhaber https://orcid.org/ 0000-0002-7043-4002

Laurel Baldwin-Ragaven https://orcid.org/ 0000-0002-6744-3768

Ronel Sewpaul https://orcid.org/ 0000-0002-2523-1222

Priscilla Reddy https://orcid.org/ 0000-0002-0432-9060

Abstract

Food insecurity impacts childhood nutritional status, physical and cognitive development, and increases lifetime risk for chronic disease. Previous South African studies have examined hunger at the sub-national level without a specific focus on children and adolescents. This study determines the national prevalence of childhood food insecurity, from birth to adolescence, and identifies factors associated with hunger within the household. Individual and household-level data were extracted from the South African National Health and Nutrition Examination Survey (SANHANES-1). Prevalence of food insecurity was assessed using the Community Childhood Hunger Identification Project (CCHIP) index. Multinomial logistic regression analyses were conducted on all households (with and without children) to determine the predictors of food insecurity, with additional analyses adjusting for child dependency and sociodemographic characteristics of household heads in households with children. Of 5 098 households surveyed, 68.6% had children and adolescents present (0-19 years). Of these households, 32.5% [95% CI (29.5 – 35.7)] were experiencing hunger and 26.3% [95% CI (23.9 – 28.8)] were at risk of hunger. Among all the households, significant associations for experiencing hunger were the presence of children and adolescents: AOR [95% CI]: 1.68 [1.12 – 2.53]; being female-headed: AOR [95% CI]: 1.53 [1.21 – 1.94] and informally-located; AOR [95% CI]: 1.61 [1.07 – 2.43]. Of the racial groups, having a non-African household head, Coloured: AOR [95% CI]: 0.29 [0.19 – 0.44] and White/Indian/Asian: 0.12 [0.04 – 0.33] conferred lower odds of experiencing hunger; and, the household head having secondary/tertiary education conferred lower odds of experiencing hunger; AOR [95% CI]: 0.40 [0.28 – 0.56] as well as being at risk of hunger; AOR [95% CI]: 0.69 [0.52 – 0.92]. Receiving social grants, pensions, or remittances more than doubled the odds of experiencing hunger; AOR [95% CI]: 2.15 [1.49 – 3.09]. After adjusting for child dependency in

households with children, having at least one older child (age 15-19 years old) did not change the odds of food insecurity. In summary, only 41% of South African households with children and adolescents were food secure. The associations between household head sociodemographics, household location and size on household food insecurity indicate a need for multi-sectoral interventions to bolster sustainable food systems for households with children and adolescents and to improve public protections for female-headed, African-headed and informally-located households dependent on social grants.

Keywords: children, adolescents, household, food insecurity, hunger, CCHIP index, South Africa, SANHANES

Introduction

Food insecurity has emerged as a major public health concern; and, the obligation to realise the "*right to food and basic nutrition"* is enshrined in both the United Nations Charter [1] and the South African Constitution [2]. The Food and Agriculture Organization (FAO) of the United Nations states that "food security exists when all people, at all times, have physical, social and economic access to sufficient, safe and nutritious food that meets their dietary needs and food preferences for an active and healthy life" [3], emphasising that the availability of food must be beyond mere subsistence [4]. In Sub-Saharan Africa, one in four people experienced undernutrition in the year 2017. This represents about one-third of the global population estimated to have suffered from chronic hunger in the same year [5]. According to actuarial projections, Africa will be home to 90% of the world's poorest by 2030 [6].

In 2017, 6.8 million South Africans were affected by hunger, representing a 50% decline since 2002; however, at the household-level, the burden of food insecurity remains largely unabated [7]. Moreover, a considerable but undocumented proportion of these households comprise children and adolescents who are vulnerable to food deprivation and hunger [4]. Indeed, it has been suggested that the presence of children in households may confer additional "*pressure of mouths to feed*" [7'8].

Throughout the world, household food insecurity is associated with malnutrition, especially stunting and wasting among children under five years old [9]. Nationally, Lake et al. [10] reported that approximately half of South African children under age five were malnourished; and, severe-acute and moderate-acute malnutrition accounts for between 4% to 25% of child mortality [10,11]. Such implications of food insecurity on health and nutrition have been observed

not only in children under five, but among older children as well. The largest national census of South African households conducted in 2011/12, the South African National Health and Nutrition Examination Survey (SANHANES), reported that the highest prevalence for wasting among children was actually among boys and girls aged 10–14 years (5.6% and 2.5% respectively); and, underweight was most prevalent among boys and girls in the 7–9 year-old age group (8.6% and 4.0% respectively) [12]. In older children, the South African Demographic and Health Survey (SADHS) showed that 27% and 9% of adolescent girls and boys aged 15-19 years respectively, were overweight or obese [13], highlighting the double burden of malnutrition across childhood and adolescence in South Africa. Importantly, hunger has profound health consequences that not only affect physical growth and cognitive development in childhood [14,15], but endure throughout the life-course into adulthood [16,17] and extend into subsequent generations [18,19]. Two longitudinal cohort studies of the consequences of hunger that began in the throes of the Second World War, the Dutch Winter Famine [19] and the siege of Leningrad [18,20] indicate that the effects of intra-uterine exposure to food insecurity can traverse through multiple generations, predisposing to a "thrifty phenotype" among progeny. Bjerregaard, et al. [21] found that another form of malnutrition, obesity in childhood (measured at ages 7 and 13 years), significantly increased the hazards of developing type-2 diabetes in late adulthood among Danish men. Conversely, reversal of obesity in adolescence reduced the risk by 4%, a finding that may be explained, in part, by food insecurity given that obesity and type-2 diabetes are primarily diet-sensitive adverse health outcomes [15].

In South Africa, the Community Childhood Hunger Identification Project (CCHIP) index was used to estimate the prevalence of food insecurity nationally between 1999 and 2008 in three

different studies: the National Food Consumption Surveys of 1999 and 2005, and the South African Social Attitudes Survey of 2008 [22]. A review of these surveys, which included children from 1-9 years old with at least one adult in the household, indicated a marked decline of household food insecurity from 52.3% in 1999 to 25.9% in 2008 [22]. These studies looked at a segment of children (those 1-9 years old), thus perhaps underestimating the true extent of child and adolescent food insecurity [23,24]. Much of what is known about the sociodemographic correlates of food insecurity in South Africa has been gleaned from sub-national cross-sectional studies. Food insecurity measured using the Household Food Insecurity Access Scale (HFIAS) indicated a gradient across the rural-urban continuum of South African towns, with households in rural areas reporting higher scores, and therefore more food insecurity, than peri-urban households (households located in urban-informal settlements) [25]. Households that are reliant on private food charities and government-assisted social grants are at an increased risk of food insecurity [26]. It is speculated that while social grants are regular and predictable, they are, however, not sensitive to the changing needs of a household [26,27]. The gender of the household head is also an important factor predicting household vulnerabilities [9], with the highest prevalence of food insecurity in South Africa reported among female-headed households [12]. In an analysis of international data, women are more likely to experience food insecurity in comparison to men, which is attributed mainly to disparities in income, education and access to social networks [28]. Even with increased employment rates among women, the income differentials between women and men has meant that poverty remains strongly gendered. Research into possible reasons for the gendered nature of food insecurity in South Africa include decades of migrant labour practices disrupting family structures, and household size [7]. Female-headed households are larger, on

average, than male-headed households and include more dependent children and adolescents, as well as are less likely to have any household member working [11].

There is a paucity of nationally representative data to assess the granular detail about food insecurity across the country. The critical need to address hunger in childhood necessitates particular attention to factors affecting food insecurity in households with children and adolescents, so that nutrition-sensitive interventions can be developed and/or strengthened [29]. Such interventions would address the underlying determinants of child hunger and include, for example, multi-sectoral approaches to social safety netting through minimum guaranteed income and/or cash transfers; financial, educational and parenting support to female-headed households with children; and bolstering health services and sustainable agricultural practices [5,29]. Such multi-pronged approaches are necessary to mediate the short-term, long-term and inter-generational adverse health consequences of food insecurity on childhood health. Therefore, drawing from a large-scale population-based sample of households, the SANHANES-1, we set out to determine the prevalence of food insecurity in households with and without children as well as to assess associations between the sociodemographic characteristics of the household head and degrees of hunger.

Methods

Study design

We conducted a secondary data analysis of the SANHANES-1 [12]. The SANHANES-1 is a cross-sectional nationally representative sample of households that employed a multi-stage disproportionate, stratified cluster sampling approach based on the 2001 census Enumeration Areas (EAs). The first 20 households were sampled from each of the 500 EAs yielding a sample of 10 000 households. Ensuring national representativeness, the SANHANES-1 EA sampling was layered by type of locality (urban/rural; formal/informal), and province (there are nine in South Africa) as well as by "race" in formal urban areas. Using race as a variable allows investigation of ongoing health disparities that have endured post-apartheid. In this way, the SANHANES captures the sociodemographic and economic profile of the country. Additional sample selection details have been published elsewhere [12]. In our study, we used the "household" as the unit of analysis. Statistics South Africa (Stats-SA) defines a household as "a group of people who live together (…), eat together and share resources, or a single person who lives alone" [30]. In the SANHANES-1, household membership was attributed to persons who occupied the same dwelling and slept in the household for at least four nights a week. The household head was identified as a physically present member who was designated internally as the head of that household, and also served as the main respondent for household-level data acquisition.

Data extraction and study population

The SANHANES-1 database includes people of all ages residing in households in South Africa. For the present study, only occupied households were included **(S1 Fig)**. Five separate but inter-related SANHANES-1 questionnaires were administered at the household-level by trained field workers capturing information on everyone living in that household (between 1 and 20+ people). The variables of interest for this study were extracted from each of the five questionnaires: visiting point (household) questionnaire (administered to the household head); child questionnaire (0-14 years old); child clinical examination form (0-14 years old); adult questionnaire (administered to anyone ≥ 15 years old); and the adult clinical examination form (≥ 15 years old). After extraction, these data were entered into four separate newly created databases. Children, whom we defined as aged 0-19 years old in order to include the adolescent population in our sample, and adults aged ≥20 years old were extracted from both the child (<15 years) and adult (≥15 years) individual-level datasets, respectively. This age selection aligns with global and national initiatives that target child and adolescent public health and nutrition [31-35]. After merging data for individuals aged 15-19 years with data from those 0-14 years old, thereby reclassifying everyone aged 0-19 years old as "children", we then reconciled them back into their corresponding households. The final sample that we analysed included 5 098 households and their occupants, both adults and children/adolescents. **S1 Fig** illustrates the database management, organisation and integration of the different household and individual-level variables from the five SANHANES-1 databases.

Ethical considerations

The original SANHANES-1 (2011/12) received ethics approval from the Research Ethics Committee (REC) of the HSRC (REC 6/16/11/11). The present secondary analysis received ethics approval from the Human Research Ethics Committee (HREC) (Medical) of the University of the Witwatersrand, Johannesburg, South Africa (Clearance certificate number: M180775).

Variables

Household composition and sociodemographic parameters

At the household-level, the parameters included were household size, the number of adults, the number of children occupying a household and child dependency status. Two variables were created to reflect household dependency status: those with children aged 0-14 years only (yes or no) and those with at least one child 15-19 years old (yes or no), who could theoretically contribute to overall household income given the legal age at which one can start working in South Africa [36]. Age, self-reported sex (male and female), self-reported race (African, Coloured, White/Indian/Asian), educational attainment (no schooling, primary, secondary, tertiary/higher degrees), marital status (married, living together/civil union, never married, widowed, separated/divorced) and the main source of income (salaries/wages, social grants/remittances/pensions, sale of products and services, no income) of all household heads were also included. Locality (urban, rural, formal and informal) was pre-assigned to the household from the EA sampling.

Household food insecurity

Food insecurity was measured using the CC[illegible]P index, a validated tool for the assessment of food insecurity at the household-level [37]. While five of the eight questions are child-referenced, making the CCHIP a specific tool for measuring *childhood* food insecurity, the CCHIP was also administered to households without children, generalising the child-referenced questions to everyone in the household. A score of ≥5 affirmative responses indicate the presence of hunger, understood as "*experiencing hunger*" and recorded as such. A score of 1-4 affirmative responses indicates that the household is "*at risk of hunger*". Lastly, a score of zero indicates that the household is "*food secure*".

Statistical analyses

STATA software, version 16.1 (STATA Institute Inc., College Station, TX, USA) was used for database management and statistical analyses. Results are presented as frequencies and percentages for categorical variables and Median [IQR] or arithmetic Mean ± SD in the case of continuous variables. Comparisons between households with and without children were performed using Mann-Whitney U tests for continuous non-normally distributed data. Categorical variables were compared using Chi-Square tests. All analyses were weighted to offset for the over-allocation of EAs in areas where Indian, Coloured or White racial groups prevailed, to ensure that the minimum required sample size in those minority groups were obtained. All analyses were performed using *svy* function in STATA, incorporating sample weights and stratified cluster sampling design to provide estimates. Weighted prevalence of food security in households with children, stratified by sociodemographic characteristics of the household heads was ascertained from frequencies and percentages; however, age differences in prevalence estimates were

computed from a Kruskal-Wallis test with a Bonferroni *post hoc* correction to adjust for multiple comparisons. To improve statistical power in the regression models, locality was dichotomised to formal vs informal; educational attainment (primary, secondary/tertiary/higher degrees and no schooling); marital status (married, never married, living together/civil unions, and widowed/separated/divorced). Weighted univariable and multivariable multinomial logistic regression analyses with robust standard errors were carried out to determine factors associated with being "at risk of hunger" and "experiencing hunger", where the "food secure" category was used as the referent group for comparison in all households, and separately in households with and without children. In households with children, we conducted additional univariable multinomial logistic regressions to examine associations between food security and child dependency, and between food security and female-headed households. A multivariable multinomial logistic regression was carried out to adjust for all of the sociodemographic factors related to the household. Statistical significance was set at $p<0.05$. Multivariable multinomial logistic regression for "at risk of hunger" and "experiencing hunger" using adjusted odds ratios with their 95% confidence intervals (CI) as measures of association are presented as forest plots, which were produced using GraphPad Prism, version 7.00 for Windows (GraphPad Software, La Jolla, California USA).

Results

Household composition, living arrangements and main source of income of household heads

Table 1 shows the characteristics of South African households with and without children, and the living arrangements of the heads of households. Of the 5 098 households with complete CCHIP scores, 3 499 (68.6%) households contained children aged 0-19 years. Households with children were occupied by 9 505 adults and 9 184 children, while those without children were occupied by 3 243 adults. Households with children were 2.5 times larger than those without children ($p<0.001$). Forty percent of all households in South Africa contained children and were female headed. Of the households containing children, nine were child-headed. When comparing households with and without children, the proportions of African- and Coloured-headed households were not different ($p=0.417$) and, no differences between urban formal and urban informal settings were found following two by two comparisons ($p=0.361$). Furthermore, there was no statistically significant difference in the main source of income for households with and without children ($p=0.381$).

Table 1. Characteristics of occupied South African households with and without children, and sociodemographic characteristics of household heads.

	All Households N=5 098		p value
	With children n=3 499	Without children n=1 599	
**Household characteristics**			
Household size (n=21 932)	5 [4 – 6]	2 [1 – 2]	<0.001[&]
No. of Adults, ≥20 years (n=12 748)	2 [2 – 3]	2 [1 – 2]	<0.001[&]
No. of Children, ≤19 years (n=9 183)	2 [1 – 3]	-	-
**Gender (n=5 092)**			
Male	1 532 (31.77)	899 (15.76)	<0.001[#]
Female	1 963 (40.44)	698 (12.02)	
**Age, years (n=5 093)**			
Mean ± SD	49.22 ± 14.67	52.07 ± 15.91	<0.001[&]
**Race (n=5 090) †**			
African	2 465 (60.88) [a]	849 (19.86)	
Coloured	659 (6.86) [b]	263 (2.48)	<0.001[#]
White/Indian/Asian	373 (4.54) [ab]	481 (5.39)	
**Locality (n=4 889) †**			
Urban formal	1 838 (39.25) [ab]	892 (16.15)	
Urban informal	464 (7.1) [cd]	155 (2.54)	<0.001[#]
Rural formal	359 (4.16) [ace]	222 (6.3)	
Rural informal	838 (22.82) [bde]	121 (1.68)	
**Marital status (n=4 998) †**			
Married	1 678 (34.68) [a]	687 (11.15)	
Living together/civil union	310 (6.1) [bc]	73 (1.28)	
Never married	757 (16.02) [abd]	486 (9.5)	<0.001[#]
Widowed	522 (11.38) [d]	236 (4.07)	
Separated/divorced	156 (3.9) [c]	93 (1.93)	
**Source of income (n=4 608)**			
Salaries or wages	1 330 (29.52)	591 (11.00)	
Social grants/pensions/remittances	1 006 (24.7)	498 (9.49)	0.381
Products and services	138 (3.33)	78 (1.71)	
No income	644 (13.98)	323 (6.27)	

Data presented as median [IQR]; arithmetic mean ± SD and n (%): unweighted (n) and weighted (%).
[&]Denotes p values obtained from Mann-Whitney U tests when comparing households with and without children for continuous variables (household size, number of adults and age of household head).
[#]Denotes p values obtained from overall Chi-Square tests when comparing households with and without children for categorical variables (gender, race, locality, and marital status).
[†]Denotes results from multiple 2x2 comparisons with Bonferroni correction: p<0.05 if categories share the same letter (a, b, c, d, and e) when comparing households with and without children. Specifically, for race and locality, p<0.0001 for African vs White/Indian/Asian, Coloured vs White/Indian/Asian, urban formal vs rural formal, and urban formal vs rural informal. For marital status, p=0.001 in married vs never married and

living together vs never married, p=0.036 in Separated/divorced vs living together/civil unions and lastly p=0.013 in never married vs widowed.

Prevalence of food insecurity in households with children

In households with children, the prevalence of *experiencing hunger* and being *at risk of hunger* was 32.5% [95% CI (29.5 – 35.7)] and 26.3% [95% CI (23.9 – 28.8)], respectively (**Fig 1**). **Table 2** shows the weighted prevalence of food insecurity stratified by the household head sociodemographic characteristics. Households in which the household head was female, older, of African race, unmarried, not having a formal education, dependent on social grants/pensions/remittances and having no income showed a higher prevalence of experiencing hunger or being at risk of hunger (p<0.001). Further analysis of the collapsed locality categories (formal vs informal) yielded the following: 37.2% of households with children were informally located; and, of these, only 28.9% were food secure. In contrast, of the 62.9% of households with children located in formal areas, 53.6% were food secure (p<0.001).

Fig 1. National prevalence of food insecurity in households with children.

Table 2. Weighted prevalence of household food security, stratified by sociodemographic characteristics of designated household heads of South African households with children.

	Total N	Food secure n (%)	†At risk of hunger n (%)	‡ Experiencing hunger n (%)	p value#
**Gender (n=3 495)**					
Male	1 532	813 (22.16)	356 (11.09) a	363 (10.75) a	<0.001
Female	1 963	739 (19.07)	502 (15.13) a	722 (21.8) a	
**Age, years (n=3 499)**					
Mean ± SD	3 499	47.87±13.85 *	49.76±15.15	50.70±15.25	0.001
**Race (n=3 497)**					
African	2 465	845 (30.18)	662 (23.16) ab	958 (30.89) ab	0.001
Coloured	659	388 (5.85)	155 (22.6) a	116 (1.38) ac	
White/Indian/Asian	373	319 (5.16)	43 (0.85) b	11 (0.27) bc	
**Marital status (n=3 423)**					
Married	1 678	899 (23.43)	375 (12.3) a	404 (12.39) abc	0.001
Living together/civil union	310	112 (3.16)	80 (22.9)	118 (3.01) a	
Never married	757	256 (6.85)	212 (6.25) a	289 (9.13) b	
Widowed	522	196 (5.69)	134 (3.98)	192 (6.12) c	
Separated/divorced	156	61 (1.96)	44 (1.38)	51 (2.07)	
**Educational attainment (n=3 499)**					
Primary	820	243 (6.48)	223 (6.28) a	354 (10.34) ab	<0.001
Secondary	1 524	762 (18.27)	390 (12.11) b	372 (11.49) acd	
Tertiary/higher degree	353	280 (8.4)	50 (1.81) abc	23 (0.81) bce	
No schooling/other	802	268 (8.05)	197 (6.05) c	337 (9.91) de	
**Source of income (n=3 118)**					
Salaries or wages	1 330	774 (22.93)	285 (9.32) ab	271 (9.01) ab	<0.001
Social grants/pensions/remittances	1 006	329 (10.69)	266 (9.34) a	411 (14.5) a	
Sale of products and services	138	71 (2.11)	37 (1.46)	30 (1.08) c	
No income	644	208 (5.54)	174 (5.86) b	262 (8.15) bc	
**Locality (n=3 499)**					
Urban formal	1 838	1 049 (27.85)	410 (12.9) ab	379 (12.77) abc	<0.001
Urban informal	464	131 (2.59)	140 (3.11) a	193 (3.98) a	
Rural formal	359	128 (2.18)	80 (1.19)	151 (2.3) b	
Rural informal	838	245 (8.58)	230 (9.05) b	363 (13.49) c	

#p value from overall Chi-Square test when comparing food security status (food secure, at risk of hunger and experiencing hunger) across categorical variables (gender, race, marital status, educational attainment, source of income and locality), of which all were statistically significant. *p<0.001 for age when comparing food secure vs at risk of hunger and food secure vs experiencing hunger (Kruskal-Wallis with Bonferroni correction). On multiple 2 by 2 analyses with Bonferroni correction, statistically significant differences within categories of sociodemographic characteristics are indicated by sharing the same letter: a, b, c, d or e, when comparing food secure vs at risk of hunger and food secure vs experiencing hunger. Specifically, p<0.01 between food secure vs. experiencing hunger except for: ‡ Primary vs no schooling, salaries vs products/services; pensions/grants vs product/services and no income; p<0.01 between food secure vs. at risk of hunger except for: †Coloured vs White/Indian/Asian; primary vs secondary; primary vs no schooling and secondary vs no schooling; salary vs product/services, pension/grants vs product/services, pension/grants vs no income and product /services vs no income; p<0.01 between food secure vs at risk of hunger and food secure vs experiencing hunger for married vs never married and married vs widower; p<0.01 between food secure vs experiencing hunger for married vs living together.

Factors associated with experiencing hunger and being at risk of hunger in all households with and without children

Weighted univariate multinomial logistic regression (**S1 Table**) of all the households exhibits that household heads who were female, of older age, unmarried/widowed/separated vs married were at higher risk of hunger and of experiencing hunger. However, when performing a weighted multivariable multinomial logistic regression (**Figs 2A and 2B**), age was no longer associated with experiencing hunger. Yet, female-headed households had 1.53 times increased odds of experiencing hunger as compared to male-headed households (p<0.001). In addition, having a larger household and a household with children present were also predictors of experiencing hunger; AOR [95% CI]: 1.06 [1.00 – 1.21]; 1.68 [1.12 – 2.53] respectively. Having a household head who was either Coloured or White/Indian/Asian as opposed to African conferred lower odds of both experiencing hunger AOR [95% CI]: 0.29 [0.19 – 0.44] and 0.12 [0.04 – 0.33] and being at risk of hunger AOR [95% CI]: 0.54 [0.39 – 0.75], 0.22 [0.12 – 0.40]. Furthermore, secondary/tertiary education conferred lower odds of both experiencing hunger; AOR [95% CI]: 0.40 [0.28 – 0.56] and being at risk of hunger; AOR [95% CI]: 0.69 [0.52 – 0.92]. Households relying on pensions/social grants/remittances, or not having any source of income, were ~1.76 to 2.15 times more at risk of hunger and experiencing hunger than those receiving a salary/wage.

Fig 2A. Factors associated with being at risk of hunger, data presented as adjusted odds ratios (AOR) and 95% Confidence intervals (95% CI).

Reference categories: *Presence of children (no children present), Gender (male), Race (African), Marital status (married), Educational attainment (primary), Source of income (salaries/wages), Locality (formal)*

Fig 2B. Factors associated with experiencing hunger, data presented as adjusted odds ratios (AOR) and 95% Confidence intervals (95% CI).

Reference categories: *Presence of children (no children present), Gender (male), Race (African), Marital status (married), Educational attainment (primary), Source of income (salaries/wages), Locality (formal)*

Female household heads and food security in households with children

Compared to male heads of households with children (**Table 3**), female household heads were older, predominantly African, never married/widowed, have primary/secondary education or no schooling, reside in rural informal settings and have no income or rely on social grants/pensions/remittances. Lastly, male-headed households had more adults in the household while female-headed households had more children of all ages.

Table 3. Sociodemographic characteristics of male and female-headed households with children.

	Total N=3 495	Male headed	Female headed	p value
		n=1 532	n=1 963	
**Age, years (n=3 495)**				
Mean ± SD	3 495	48.43±13.86	49.81±15.24	0.0423
**Race (n=3 493)[&]**				
African	2 462	976 (78.99)	1 486 (88.40)	
Coloured	658	324 (11.73)	334 (7.67)	0.0001
White/Indian/Asian	373	231 (9.29)	142 (3.94)	
**Marital status (n=3 420)[#]**				
Married	1 677	1 078 (71.39)	599 (29.84)	
Living together/civil unions	309	222 (13.94)	87 (4.07)	<0.001
Never married	757	132 (9.09)	625 (32.63)	
Widowed	521	53 (3.58)	468 (25.35)	
Separated/divorced	156	28 (1.99)	128 (8.11)	
**Educational attainment (n=3 495)[^]**				
Primary	820	307 (18.28)	516 (26.96)	
Secondary	1 521	706 (44.03)	815 (40.08)	<0.001
Tertiary/Higher degrees	353	215 (16.24)	138 (6.96)	
No schooling/other	801	388 (21.45)	494 (26.00)	
**Source of income (n=3 116)[$]**				
Salaries or wages	1 328	798 (55.44)	530 (29.45)	
Social grants/pensions/Remittances	1 006	305 (23.66)	701 (43.58)	<0.001
Sale of products and services	138	76 (5.46)	62 (4.00)	
No income	644	225 (15.45)	419 (22.97)	
**Locality (n=3 495)***				
Urban formal	1 836	874 (60.13)	962 (48.31)	
Urban informal	464	178 (8.28)	286 (10.82)	<0.001
Rural formal	359	191 (7.27)	168 (4.44)	
Rural informal	836	289 (24.32)	547 (36.43)	
**Composition, Median [IQR]**				
Household size	3 495	5 [4-6]	5 [3-6]	0.090
No. of adults	3 495	3 [2-4]	2 [2-3]	<0.001
No. of children	3 495	2 [1-3]	2 [1-4]	<0.001
No. of children aged 0-14 years	1814	2 [1-3]	2 [1-3]	<0.001
No. of children aged 15-19 years	1681	2 [2-4]	3 [2-4]	<0.001

&Race: African vs Coloured (p=0.001) and African vs White/Indian/Asian (p=0.001)
#Marital status: married vs never married (p<0.001), married vs widowed (p<0.001), married vs separated/divorced (p<0.001), living together/civil union vs never married (p<0.001), living together/civil union vs widowed (p<0.001), living together/civil union vs separated/divorced (p<0.001), and never married vs widowed (p=0.0015)
^Educational attainment: primary vs secondary (p=0.0002), primary vs tertiary/higher degrees (p<0.001), secondary vs tertiary/higher degrees (p=0.0001), and tertiary vs no schooling (p<0.001)
$Source of income: salaries/wages vs social grants/pensions/remittances (p<0.001), salaries/wages vs no income (p<0.001), and social grants/pensions/remittances vs sale of products and services (p=0.0002)
*Locality: urban formal vs urban informal (p=0.001), urban formal vs rural informal (p<0.001), urban informal vs rural informal (p=0.0010), and rural formal vs rural informal (p<0.001).

In the univariate analysis of the gender of the household head and food security in households with children, female-headed households had greater odds of being at risk of hunger [OR 1.58; 95% CI: 1.22-2.04, p<0.001] and experiencing hunger [OR 2.35; 95% CI: 1.86-2.98, p<0.001].

Child dependency and sociodemographic characteristics of household heads, in households with children

In the univariate analysis of child dependency and food security in households with children, having at least one child who was 15 years or older increased the odds of experiencing hunger by 28% (OR 1.28; 95% CI: 1.06-1.56, p=0.012). **Table 4** shows associations between food security and child dependency, adjusted for the sociodemographic characteristics of the household heads. Compared to households with younger children only, having at least one child aged 15-19 years old in a household was not associated with either being at risk of hunger or experiencing hunger (no significant change in adjusted odds ratio). Having a household head who was male, Coloured/White/Indian/Asian, employed and having obtained tertiary education/higher degrees conferred lower odds of experiencing hunger. Residing in informal settings increased the odds of both being at risk of hunger and experiencing hunger.

Table 4: Weighted multivariable multinomial regression analysis between food security and child dependency, adjusted for sociodemographic characteristics of the heads of households with children.

Characteristics	At risk of hunger			Experiencing hunger		
	AOR	95% CI	p value	AOR	95% CI	p value
Household size	0.98	0.92 – 1.04	0.604	1.04	0.99 – 1.10	0.102
**Child dependency**						
Children aged 0-14 years only	***REF***	***REF***	***REF***	***REF***	***REF***	***REF***
At least one child aged 15-19 years	1.01	0.78 – 1.30	0.934	1.24	0.95 – 1.62	0.105
**Age, years**						
Age	0.99	0.98 – 1.01	0.336	0.98	0.97 – 1.00	0.089
**Gender**						
Female	***REF***	***REF***	***REF***	***REF***	***REF***	***REF***
Male	0.92	0.66 – 1.27	0.603	0.72	0.53 – 0.98	0.041
**Race**						
African	***REF***	***REF***	***REF***	***REF***	***REF***	***REF***
Coloured	0.60	0.42 – 0.87	0.006	0.33	0.21 – 0.50	<0.001
White/Indian/Asian	0.17	0.09 – 0.34	<0.001	0.12	0.04 – 0.34	<0.001
**Marital status**						
Living together/civil union	***REF***	***REF***	***REF***	***REF***	***REF***	***REF***
Married	0.88	0.59 – 1.32	0.552	0.61	0.39 – 0.94	0.027
Never married	0.88	0.51 – 1.15	0.652	0.83	0.51 – 1.36	0.478
Widowed	0.67	0.37 – 1.19	0.172	0.63	0.35 – 1.12	0.117
Separated/divorced	1.28	0.59 – 2.75	0.526	1.83	0.54 – 2.56	0.673
**Educational attainment**						
No schooling/other	***REF***	***REF***	***REF***	***REF***	***REF***	***REF***
Primary	1.23	0.82 – 1.83	0.310	1.37	0.94 – 1.99	0.095
Secondary	1.06	0.72 – 1.58	0.742	0.70	0.46 – 1.05	0.086
Tertiary/Higher degree	0.50	0.28 – 0.89	0.019	0.18	0.09 – 0.34	<0.001
**Source of income**						
No income	***REF***	***REF***	***REF***	***REF***	***REF***	***REF***
Pensions/Grants/Remittances	0.91	0.60 – 1.36	0.651	0.82	0.55 – 1.21	0.331
Sale of products and services	0.73	0.40 – 1.34	0.323	0.40	0.19 – 0.81	0.011
Salaries and/or wages	0.51	0.34 – 0.77	0.001	0.38	0.25 – 0.57	<0.001
**Locality**						
Formal	***REF***	***REF***	***REF***	***REF***	***REF***	***REF***
Informal	1.57	1.15 – 2.15	0.004	1.66	1.18 – 2.34	0.003

Discussion

To the best of our knowledge, the present study is the first to objectively quantify the burden of food insecurity among children and adolescents in South Africa and draw associations using a large-scale, population-based sample of households with children. No studies in South Africa have disaggregated households with and without children aged 0-19 years old thus possibly underestimating the true extent of childhood food insecurity and limiting direct comparisons to the present study. Indeed, there is an observed increase in food insecurity (experiencing hunger—25.9% to 32.5%) by 6.6% between the prevalence estimate reported for 2008 by Labadarios et al. [22] and the estimate generated in the present study. This lack of improvement may be accounted for by the enduring socio-political and economic challenges sustained since the end of apartheid [22,38]. Drawing from a cross-sectional, population-based national sample of South African households, we have shown that the prevalence of food insecurity in households with children was nearly 60%. This is similar to the original SANHANES study where food insecurity among all households was 54% [12]. In the present study, after adjusting for household size, the sociodemographic characteristics of the household head and the main source of income, the presence of children and adolescents conferred ~1.68 increased odds of food insecurity (experiencing hunger) relative to households without children. In addition, having a female vs male household head, having an African household head compared to all other race groups, living in informal settings vs formal settings and having no income compared to relying on salaries or wages increased the odds of experiencing hunger between 53% and 300%. Secondary/tertiary/higher educational attainment of the household head reduced the odds of experiencing hunger by at least half relative to primary education. Reliance on social grants, pensions and remittances as the main source of income was associated with more than double the

odds of being food insecure. When analysing food security in households with at least one adolescent aged 15-19 years, versus those with children aged 0-14 years only, before adjusting for the sociodemographics of the household head and location, the odds of being food insecure were almost 30% higher compared to households with younger children only. However, after controlling for these factors, neither child dependency nor the ability to work were associated with any category of food security.

Household head sociodemographics and household characteristics

The majority of households with children and adolescents in our sample was headed by women, had African household heads, or were living in urban or rural informal areas. Therefore, the increased vulnerability of households with children may be explained, in part, by the additive effects of these known predictors of food insecurity [39]. Previous studies in other contexts have found that the presence of children in households is independently associated with food insecurity, beyond sociodemographic characteristics and measures of socioeconomic status [7,39-43]. Additional research has demonstrated that the sex of the household head is an important factor predicting household vulnerabilities, with the highest prevalence of hunger reported among female-headed households [27], a risk that worsens with rural location [25,44]. Our comparative analysis of female- versus male-headed households points to the stark sociodemographic disadvantages that women heads endure in households with children, confirming previous research in South Africa on the gendered nature of poverty and food insecurity [27,45]. In their large-scale, population-based longitudinal analysis, the Indonesian Family Life Survey, Vaezghasemi et al.

[46] further argue that although women may be conscious of healthy food options, because they are at the bottom of the family hierarchy and have low social capital, they remain defenseless against household hunger. Given South Africa's colonial and apartheid history, racial differences still persist in the prevalence of food insecurity, with black or African households bearing the greatest burden compared to historically more advantaged minority groups in the country [7,11,12,25].

Previous studies have demonstrated strong positive associations between the lowest levels of educational attainment and food insecurity, which can be mitigated by higher levels of educational attainment [5,47-50]. Likewise, we found that improved levels of education (secondary and tertiary educational attainment) reduced the risk of food insecurity by at least half; yet, having no formal schooling was not associated with food insecurity. As Chakona and Shackleton also found [25], we suggest that household heads without a formal education may rely on subsistence farming or implement other skills-based practices which might act as a safety net against hunger.

The Born in Bradford cohort, a longitudinal study in the United Kingdom, showed associations between cohabitation status and food insecurity, where pregnant women not living with a partner had a two-fold increased risk of food insecurity [51]. Hanson, Sobal, and Frongillo [52] further demonstrated that marital status was associated with food insecurity among men, but not women. We found significant associations between the marital status of the household head and risk of experiencing hunger. Even after adjustments for sex, living together/civil unions and never married increased the odds of experiencing hunger by 61% and 41%, respectively.

Main source of household income, social grants and child dependency

Using self-reported main source of income to indicate a household's economic stability, we note that there were no differences in income sources between households with and without children. However, household size was larger in households with children, including an increased number of adults. We show that not having any source of income predicts that a household will experience a three-fold increase in experiencing hunger. Furthermore, similar to Ruiters and Wildschutt [27], receiving government social grants, pensions or remittances did not alleviate hunger nor the risk of being food insecure for households with and without children. Despite a substantial social safety net wherein 16.2 million South Africans in 2013, representing nearly one third of the country's population received different types of government grants [53], these were not protective in households with children, with a 76% increase in being at risk of food insecurity and double the odds for being food insecure. Other contexts show mixed results for government assistance programmes on alleviating food insecurity. Tarasuk, Fafard St-Germain and Mitchell. [54] have shown that in Canada, household before-tax income adjusted for family size was protective against food insecurity; however, households receiving social assistance were three times more likely to be food insecure. In contrast, Brown and Tarasuk. [55] showed a significant decline in severe food insecurity subsequent to the roll-out of the Canada Child Benefit (CCB) in households with children, a country-wide non-means-tested cash transfer programme. In Brazil, participation in the Bolsa Familia Programme [56] and in the United States receiving Supplemental Nutrition Assistance Program (SNAP, formerly the Food Stamp Program) support [8,57], were not effective in preventing food insecurity. In one South African study where social grants did show

improvements in household food insecurity and dietary quality, the authors note that these findings were not accompanied by concomitant improvements in anthropometric indicators of child malnutrition [26]. Even when controlling for the sociodemographic characteristics of the household head and the main source of income, the risk of experiencing hunger was increased with the presence of children in all households. This is similar to the findings of a 2017 national study comparing households with and without children under the age of 5 years, whereby households with no children were less likely to experience inadequate access to food [7]. In our sample of households with children, controlling for child dependency status (ages 0-14 versus 15-19 years old) did not lessen the odds of experiencing hunger. Regarding an older child's potential to contribute towards household income through work eligibility, Stats-SA reported in 2012, the year of the SANHANES-1, an overall youth unemployment rate of 35.8% (age 15-34 years old) as compared to an adult unemployment rate of 15.1% [53]. After disaggregating youth unemployment by age and sex, males and females aged 15-19 years had rates of 50.2% versus 74% respectively [53], indicating that child dependency on an adult household head extends throughout adolescence in South Africa.

Contemporary considerations and current context

The present COVID-19 pandemic, occurring alongside household and child food and nutrition insecurity, presents a disconcerting syndemic [58] that is capable of reversing any recent mitigation of hunger at household and individual levels in the country [59]. Indeed, the COVID-19 pandemic has forced South Africa into an abrupt and prolonged lockdown threatening job and societal security [59]. Data from the National Income Dynamics Study-Coronavirus Rapid Mobile Survey (NID-CRAMS) [60], a four-wave longitudinal survey of adult South Africans, indicate that

even with re-opening of the economy, lifting restrictions on movement and returning to work, 37-47% households did not have sufficient money to buy food, with 16-22% stating that they experienced hunger, during the period from May/June 2020 to February/March 2021 [60]. Overall, while hunger declined somewhat over this period, the poorest quintile suffered more hunger than before [60]. Without augmented social supports for households with children and adolescents, and an emphasis on nutrition-sensitive interventions [29,50,61], it is likely that food insecurity will pervade [14] and possibly impede the country from realising its 2030 Sustainable Development Goal of achieving zero hunger [31,58].

Limitations

The present study is not without limitations. The SANHANES-1 was conducted in 2011/12. Although never repeated, the data are at least nine years old and may not reflect the current status of food security in South Africa. Nonetheless, the SANHANES provides the most recent and comprehensive dataset on food security at the national and household-level using the CCHIP index that explores the context and loci within which food insecurity exists. This is in contrast with other national surveys which only record the frequency of problems satisfying food needs "in the past 12 months" [13]. Secondly, a comprehensive assessment of household socioeconomic status was beyond the scope of this research. Having considered the primary source of household income and educational status as contributing factors to household economic stability, a more robust measure of socioeconomic status using household assets could add a more granular dimension to the analysis.

Conclusion

Our study confirms that South African children and adolescents face a major challenge of food insecurity that is driven by a number of household head characteristics and household location. The untoward effects of food insecurity on child and adolescent health are not evanescent and have far-reaching adverse health outcomes. Therefore, this is a clarion call to bolster multi-sectoral interventions and healthy public policies, with a strong focus on the social determinants of health and poverty eradication strategies targeting these vulnerable households.

Acknowledgements

The authors acknowledge Dr Innocent Maposa for his assistance with data analysis.

References

1. United Nations Office of the High Commissioner for Human Rights. Fact Sheet No. 34, The Right to Adequate Food [Internet]. Refworld. 2021 [cited 2021 Jun 7]. Available from: https://www.refworld.org/docid/4ca460b02.html.
2. Food and Agriculture Organization. The Right to Food around the Globe [Internet]. Food and Agriculture Organization of the United Nations. 2015 [cited 2021 Jun 6]. Available from: http://www.fao.org/right-to-food-around-the-globe/countries/zaf/en/.
3. Skoet J, Stamoulis KG. The state of food insecurity in the world 2006 [Internet]. Food and Agriculture Organization. 2006 [cited 2021 Jun 7]. Available from: http://www.fao.org/3/a0750e/a0750e00.pdf.
4. Neuman N, Eli K, Nowicka P. Feeding the extended family: gender, generation, and socioeconomic disadvantage in food provision to children. Food, Cult Soc [Internet]. 2019; 22(1):45–62. https://doi.org/10.1080/15528014.2018.1547066.
5. Fraval S, Hammond J, Bogard JR, Ng'endo M, van Etten J, Herrero M, et al. Food access deficiencies in sub-Saharan Africa: prevalence and implications for agricultural interventions. Front. Sustain. Food Syst. 2019 Nov 19; 3:104. https://doi.org/10.3389/fsufs.2019.00104.
6. The World Bank Annual Report 2019: Ending Poverty, Investing in Opportunity [Internet]. Washington, DC: World Bank. 2019 [cited 2021 Jun 7]. Available from: https://openknowledge.worldbank.org/handle/10986/32333.
7. Towards measuring food security in South Africa: An examination of hunger and food inadequacy [Internet]. Stats-SA. 2019. [cited 2021 Jun 7]. Available from: http://www.statssa.gov.za/publications/03-00-14/03-00-142017.pdf.

8. Nord M. Food Insecurity in Households with Children: Prevalence, Severity, and Household Characteristics. Economic Information Bulletin Number 56. US Department of Agriculture. 2009 Sep. http://www.usda.gov/wps/portal/usdahome.
9. Webb P, Stordalen GA, Singh S, Wijesinha-Bettoni R, Shetty P, Lartey A. Hunger and malnutrition in the 21st century. BMJ [Internet]. 2018 Jun 13 [cited 2021 Jun 14]. doi: https://doi.org/10.1136/bmj.k2238
10. Lake L, Shung-King M, Hendricks M, Heywood M, Nannan N, Laubscher R, et al. Prioritising child and adolescent health: A human rights imperative. In: Shung-King M, Lake L, Sanders D, Hendricks M (eds). South African Child Gauge 2019: Child and adolescent health 2019. Cape Town: Children's Institute, University of Cape Town. p.32-62. ISBN: 978-0-620-85838-0.
11. Hall K, Nannan N, Sambu W. Child health. In: Hall K, Richter L, Mokomane Z, Lake L (eds). South African Child Gauge 2018: Children, Families, and the State 2018. Cape Town: Children's Institute, University of Cape Town. p.144-149. ISBN: 978-0-620-81934-3.
12. Shisana O, Labadarios D, Rehle T, Simbayi L, Zuma K, Dhansay A, et al. South African National Health and Nutrition Examination Survey, 2012 [Internet]. Human Sciences Research Council. 2013 [cited 2021 Jun 7]. Available at: ISBN (e-pub) 978-0-7969-2478-0.
13. South Africa Demographic and Health Survey 2016 Key Findings [Internet]. Available from: https://dhsprogram.com/pubs/pdf/SR248/SR248.pdf.
14. Perez-Escamilla F, de Toledo Vianna RP. Food Insecurity and the Behavioral and Intellectual Development of Children: A Review of the Evidence. J. Appl. Res. Child.

2012; 3(1):9. Available at: http://digitalcommons.library.tmc.edu/childrenatrisk/vol3/iss1/9.

15. Seligman HK, Laraia BA, Kushel MB. Food insecurity is associated with chronic disease among low-income NHANES participants. J. Nutr. 2010 Feb 1; 140(2):304-10. https://doi.org/10.3945/jn.109.112573.

16. Kirkpatrick SI, McIntyre L, Potestio ML. Child hunger and long-term adverse consequences for health. Arch. pediatr. adolesc. med. 2010 Aug 1; 164(8):754-762. doi:10.1001/archpediatrics.2010.117.

17. Ke J, Ford-Jones EL. Food insecurity and hunger: A review of the effects on children's health and behaviour. Paediatr Child Health. 2015 Mar 1; 20(2):89-91. doi.org/10.1093/pch/20.2.89.

18. Sparén P, Vågerö D, Shestov DB, Plavinskaja S, Parfenova N, Hoptiar V, et al. Long term mortality after severe starvation during the siege of Leningrad: prospective cohort study. BMJ. 2004 Jan 1; 328(7430):11. https:// doi.org/10.1136/bmj.37942.603970.9A.

19. Lumey LH, Stein AD, Kahn HS, Van der Pal-de Bruin KM, Blauw GJ, Zybert PA, et al. Cohort profile: the Dutch Hunger Winter families study. Int. J. Epidemiol. 2007 Dec 1; 36(6):1196-204. https://doi.org/10.1093/ije/dym126.

20. Calkins K, Devaskar SU. Fetal origins of adult disease. Curr Probl Pediatr Adolesc Health Care. 2011 Jul 1; 41(6):158-76. https://doi.org/10.1016/j.cppeds.2011.01.001.

21. Bjerregaard LG, Jensen BW, Ängquist L, Osler M, Sørensen TI, Baker JL. Change in overweight from childhood to early adulthood and risk of type 2 diabetes. NEJM. 2018 Apr 4. doi: 10.1056/NEJMoa1713231.

22. Labadarios D, Mchiza ZJ, Steyn NP, Gericke G, Maunder EM, Davids YD, et al. Food security in South Africa: a review of national surveys. Bull. World Health Organ. 2011; 89:891-9. doi: 10.2471/BLT.11.089243.

23. Labadarios D, Steyn NP, Maunder E, Macintryre U, Gericke G, Swart R, et al. The national food consumption survey (NFCS): South Africa, 1999. Public Health Nutr. 2005 Aug; 8(5):533-43. https://doi.org/10.1079/PHN2005816.

24. Labadarios D, Swart R, Maunder EM, Kruger HS, Gericke GJ, Kuzwayo PM, et al. Executive summary of the National Food consumption Survey Fortification Baseline (NFCS-FB-I). S Afr J Clin Nutr. 2008; 21(3 Suppl 2): 247–300.

25. Chakona G, Shackleton CM. Household food insecurity along an agro-ecological gradient influences children's nutritional status in South Africa. Front. Nutr. 2018 Jan 22; 4:72. https://doi.org/10.3389/fnut.2017.00072.

26. Waidler J, Devereux S. Social grants, remittances, and food security: does the source of income matter? Food Secur. 2019 Jun; 11(3):679-702. https://doi.org/10.1007/s12571-019-00918-x.

27. Ruiters M, Wildschutt A. Food insecurity in South Africa: Where does gender matter?. Agenda. 2010 Jan 1; 24(86):8-24. doi: 10.1080/10130950.2010.10540516.

28. Broussard NH. What explains gender differences in food insecurity? Food Policy. 2019 Feb 1; 83:180-94. https://doi.org/10.1016/j.foodpol.2019.01.003.

29. Ruel MT, Alderman H, Maternal and Child Nutrition Study Group. Nutrition-sensitive interventions and programmes: how can they help to accelerate progress in improving maternal and child nutrition? Lancet. 2013 Aug 10; 382(9891):536-51. https://doi.org/10.1016/S0140-6736(13)60843-0.

30. Statistics South Africa. General Household Survey 2017 [Internet]. Stats-SA. 2018 [cited 2021 Dec 31]. Available from: https://www.statssa.gov.za/publications/P0318/P03182018.pdf

31. Pérez-Escamilla R. Food security and the 2015–2030 sustainable development goals: From human to planetary health: Perspectives and opinions. Curr Dev Nutr. 2017 Jul 1; 1(7):e000513. https://doi.org/10.3945/cdn.117.000513.

32. Onis MD, Onyango AW, Borghi E, Siyam A, Nishida C, Siekmann J. Development of a WHO growth reference for school-aged children and adolescents. Bull. World Health Organ. 2007; 85:660-7. doi: 10.2471/blt.07.043497.

33. Republic of South Africa—National Planning Commission. National Development Plan 2030: Our future-make it work. ISBN: 978-0-621-41180-5

34. Kuruvilla S, Bustreo F, Kuo T, Mishra CK, Taylor K, Fogstad H, et al. The Global strategy for women's, children's and adolescents' health (2016–2030): a roadmap based on evidence and country experience. Bull. World Health Organ. 2016 May 1; 94(5):398. doi: 10.2471/BLT.16.170431.

35. Dick B, Ferguson BJ. Health for the world's adolescents: a second chance in the second decade. J Adolesc Health. 2015 Jan 1; 56(1):3-6. https://doi.org/10.1016/j.jadohealth.2014.10.260.

36. Statistics SA. National and provincial labour market: Youth. Statistics South Africa, Pretoria. 2015. https://www.statssa.gov.za/publications/P02114.2/P02114.22015.pdf

37. Swindale A, Bilinsky P. Development of a universally applicable household food insecurity measurement tool: process, current status, and outstanding issues. J. Nutr. 2006 May 1; 136(5):1449S-52S. https://doi.org/10.1093/jn/136.5.1449S.

38. Drimie S, Ruysenaar S. The integrated food security strategy of South Africa: an institutional analysis. Agrekon. 2010 Sep 1;49(3):316-37. https://doi.org/10.1080/03031853.2010.503377.

39. Grobler WC. Perceptions of poverty: a study of food secure and food insecure households in an urban area in South Africa. Procedia Economics and Finance. 2016 Jan 1; 35:224-31. https://doi.org/10.1016/S2212-5671(16)00028-9

40. Chinnakali P, Upadhyay RP, Shokeen D, Singh K, Kaur M, Singh AK, et al. Prevalence of household-level food insecurity and its determinants in an urban resettlement colony in north India. J Health Popul Nutr. 2014 Jun;32(2):227. PMID: 25076660.

41. Mortazavi Z, Dorosty AR, Eshraghian MR, Ghaffari M, Ansari-Moghaddam A, Mohammadi M. Household food insecurity in Southeastern Iran: severity and related factors. Int. J. Food Sci. 2017 Nov 15;2017. https://doi.org/10.1155/2017/7536024.

42. Tantu AT, Gamebo TD, Sheno BK, Kabalo MY. Household food insecurity and associated factors among households in Wolaita Sodo town, 2015. Agric. Food Secur. 2017 Dec; 6(1):1-8. https://doi.org/10.1186/s40066-017-0098-4.

43. Sisha TA. Household level food insecurity assessment: Evidence from panel data, Ethiopia. Sci. Afr. 2020 Mar 1; 7:e00262. https://doi.org/10.1016/j.sciaf.2019.e00262.

44. Hossain M, Choudhury N, Abdullah KA, Mondal P, Jackson AA, Walson J, et al. Evidence-based approaches to childhood stunting in low and middle income countries: a systematic review. Arch. Dis. Child. 2017 Oct 1; 102(10):903-9. http://dx.doi.org/10.1136/archdischild-2016-311050.

45. Molestane R, Reddy V. The gendered dimensions of food security in South Africa: A review of the literature. Policy Brief, Human Sciences Research Council, Pretoria. 2011. Available at: http://hdl.handle.net/20.500.11910/4887

46. Vaezghasemi M, Öhman A, Eriksson M, Hakimi M, Weinehall L, Kusnanto H, et al. The effect of gender and social capital on the dual burden of malnutrition: a multilevel study in Indonesia. PloS one. 2014 Aug 25; 9(8):e103849. https://doi.org/10.1371/journal.pone.0103849.

47. Kim J, Lim H. Nutritional management in childhood obesity. J. obes. metab. syndr. 2019 Dec; 28(4):225. doi: 10.7570/jomes.2019.28.4.225.

48. Gebremedhin S. Prevalence and differentials of overweight and obesity in preschool children in Sub-Saharan Africa. BMJ open. 2015 Dec 1; 5(12):e009005. http://dx.doi.org/10.1136/bmjopen-2015-009005.

49. Devereux S, Hochfeld T, Karriem A, Mensah C, Morahanye M, Msimango T, et al. Food Security SA Working Paper Series: #004 School Feeding in South Africa: What we know, what we don't know, what we need to know, what we need to do Authors [Internet]. ; Available from: https://foodsecurity.ac.za/wp-content/uploads/2018/06/CoE-FS-WP4-School-Feeding-in-South-Africa-11-jun-18.pdf.

50. Bhutta ZA, Das JK, Rizvi A, Gaffey MF, Walker N, Horton S, et al. Evidence-based interventions for improvement of maternal and child nutrition: what can be done and at what cost? Lancet. 2013 Aug 3; 382(9890):452-77. https://doi.org/10.1016/S0140-6736(13)60996-4.

51. Power M, Uphoff EP, Stewart-Knox B, Small N, Doherty B, Pickett KE. Food insecurity and socio-demographic characteristics in two UK ethnic groups: an analysis of women in

the Born in Bradford cohort. J Public Health. 2018 Mar 1; 40(1):32-40. https://doi.org/10.1093/pubmed/fdx029.

52. Hanson KL, Sobal J, Frongillo EA. Gender and marital status clarify associations between food insecurity and body weight. J Nutr. 2007 Jun 1; 137(6):1460-5. https://doi.org/10.1093/jn/137.6.1460.

53. Cassim A, Morne Oosthuizen. The State of Youth Unemployment in South Africa [Internet]. Brookings. Brookings; 2014 [cited 2021 Dec 26]. Available from: https://www.brookings.edu/blog/africa-in-focus/2014/08/15/the-state-of-youth-unemployment-in-south-africa/

54. Tarasuk V, St-Germain AA, Mitchell A. Geographic and socio-demographic predictors of household food insecurity in Canada, 2011–12. BMC public health. 2019 Dec; 19(1):1-2. https://doi.org/10.1186/s12889-018-6344-2.

55. Brown EM, Tarasuk V. Money speaks: Reductions in severe food insecurity follow the Canada Child Benefit. Prev. Med. 2019 Dec 1; 129:105876. https://doi.org/10.1016/j.ypmed.2019.105876.

56. Costa NS, Santos MO, Carvalho CP, Assunção ML, Ferreira HS. Prevalence and factors associated with food insecurity in the context of the economic crisis in Brazil. Curr Dev Nutr. 2017 Oct 1; 1(10):e000869. https://doi.org/10.3945/cdn.117.000869.

57. Coleman-Jensen A, Gregory C, Singh A. Household food security in the United States in 2013. USDA-ERS Economic Research Report. 2014 Sep 1(173). http://dx.doi.org/10.2139/ssrn.2504067.

58. Pérez- Escamilla R, Cunningham K, Moran VH. COVID- 19 and maternal and child food and nutrition insecurity: a complex syndemic. Matern. Child Nutr. 2020 Jul; 16(3):e13036. https://doi.org/10.1111/mcn.13036.

59. International Labour Organization. ILO Monitor: COVID-19 and the World of Work. Updated estimates and analysis. Int Labour Organ. 2020 Apr 29. Available from: https://www.ilo.org/global/about-the-ilo/WCMS_738753/lang--en/index.htm.

60. Patel L, Bridgman G. Hunger in South Africa during 2020: Results from Wave 3 of NIDS-CRAM [Internet]. CRAM. 2021 [cited 2021 Jun 7]. Available from: https://cramsurvey.org/wp-content/uploads/2021/02/10.-Van-der-Berg-S.-Patel-L.-Bridgman-G.-2021-Hunger-in-South-Africa-during-2020-Results-from-Wave-3-of-NIDS-CRAM-1.pdf.

61. Bhutta ZA, Das JK, Rizvi A, Gaffey MF, Walker N, Horton S, et al. Maternal and Child Nutrition Study Group. Evidence-based interventions for improvement of maternal and child nutrition: what can be done and at what cost? Lancet. 2013 Aug 3; 382(9890):452-77. https://doi.org/10.1016/S0140-6736(13)60996-4.

Supporting information

S1 Fig. Database management, organisation and integration of data extracted from the four household and individual - level datasets of the SANHANES-1

S1 Table. Weighted univariable multinomial logistic regression analysis of factors associated with being at risk of hunger and experiencing hunger against being food secure in households with and without children

S1 Dataset. Final analytic sample of households with food security categories

Fig1

Click here to access/download;Figure;Fig 1.tif

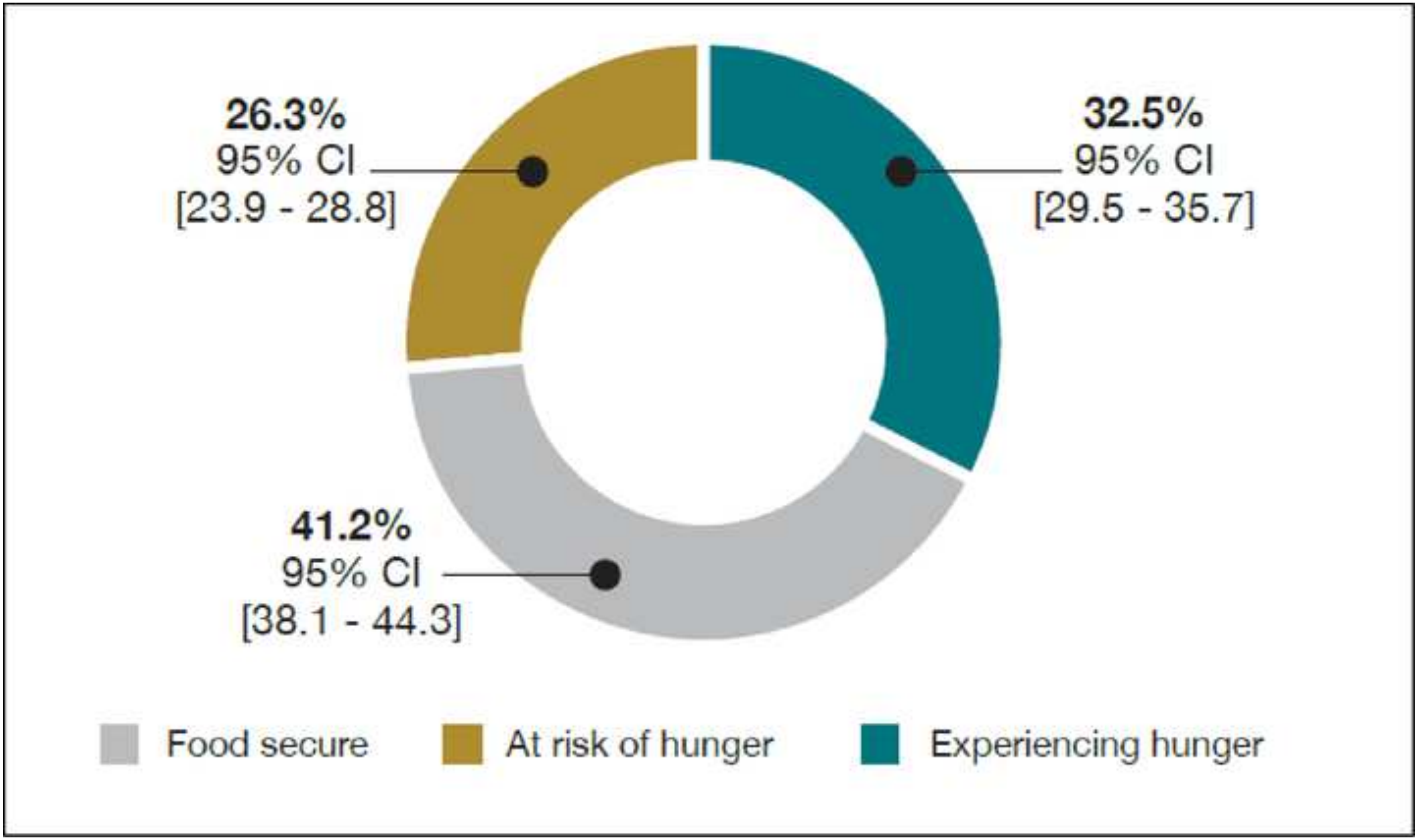

Fig2A

Click here to access/download;Figure;Fig 2A.tif

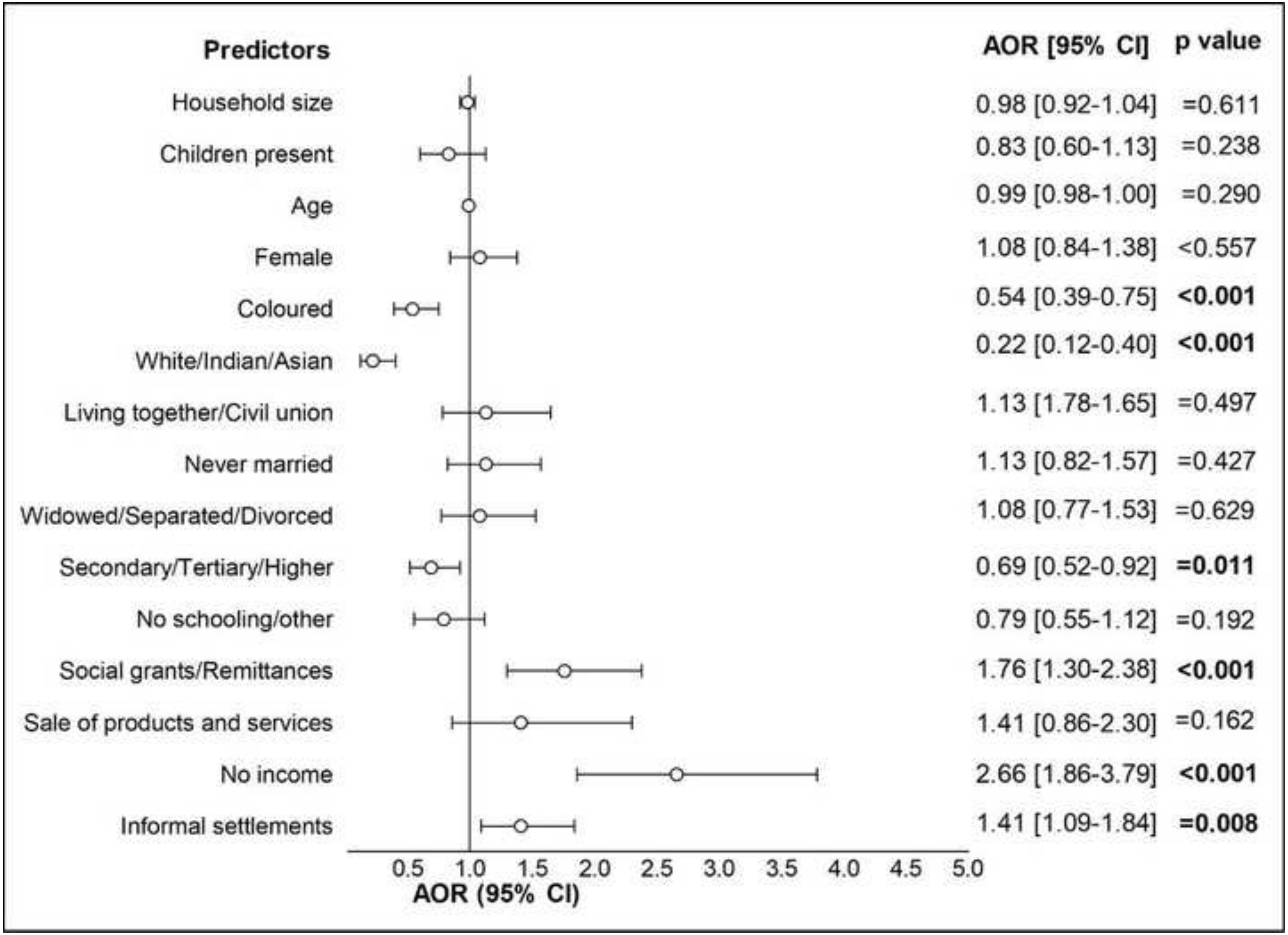

Fig2B

Click here to access/download;Figure;Fig 2B.tif

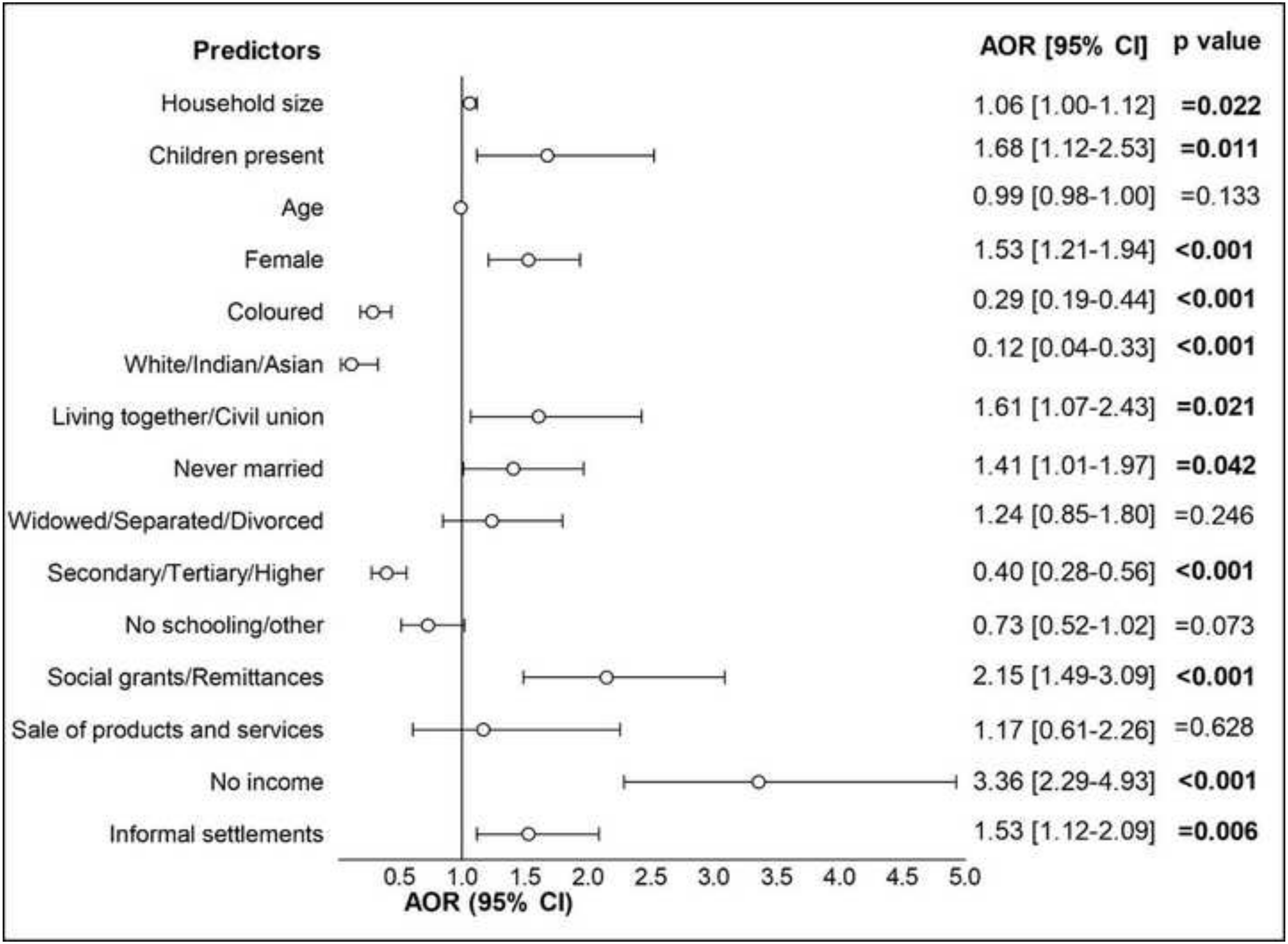

Click here to access/download
Supporting Information
S1 Fig.docx

Supporting Information_S1_data

Click here to access/download
Supporting Information
S1 Database.csv

Click here to access/download
Supporting Information
S1 Table file.docx

Childhood food insecurity in South Africa:

A household-level analysis of hunger

Siluleko A. Mkhize[1], Elena Libhaber[2], Ronel Sewpaul[3], Priscilla Reddy[3,4] and Laurel Baldwin-Ragaven[1*]

[1]Department of Family Medicine and Primary Care, School of Clinical Medicine, Faculty of Health Sciences, University of the Witwatersrand, Johannesburg, South Africa

[2]School of Clinical Medicine and Health Sciences Research Office, Faculty of Health Sciences, University of the Witwatersrand, Johannesburg, South Africa

[3]Health and Wellbeing, Human and Social Capabilities Division, Human Sciences Research Council, Cape Town, South Africa

[4]Faculty of Health Sciences, Nelson Mandela University, Port Elizabeth, South Africa

*****Corresponding author

E-mail: laurel.baldwin-ragaven@wits.ac.za (LBR)

Contributions

SM, LBR and EL conceptualised the study and its design. SM, EL, LBR and RS conducted and interpreted the analyses. SM wrote the first draft of the manuscript; and, EL LBR and RS worked on subsequent iterations of the manuscript. RS and PR critically revised the manuscript for important intellectual content. All authors approved the final manuscript and take primary responsibility for its contents. The contents of the manuscript are solely the responsibility of the

authors and do not necessarily represent the official views of the HSRC or the University of the Witwatersrand, Johannesburg, South Africa.

ORCID

Siluleko Mkhize https://orcid.org/ 0000-0002-8351-6891

Elena Libhaber https://orcid.org/ 0000-0002-7043-4002

Laurel Baldwin-Ragaven https://orcid.org/ 0000-0002-6744-3768

Ronel Sewpaul https://orcid.org/ 0000-0002-2523-1222

Priscilla Reddy https://orcid.org/ 0000-0002-0432-9060

Abstract

Food insecurity impacts childhood nutritional status, physical and cognitive development, and increases lifetime risk for chronic disease. Previous South African studies have examined hunger at the sub-national level without a specific focus on children and adolescents. This study determines the national prevalence of childhood food insecurity, from birth to adolescence, and identifies factors associated with hunger within the household. Individual and household-level data were extracted from the South African National Health and Nutrition Examination Survey (SANHANES-1). Prevalence of food insecurity was assessed using the Community Childhood Hunger Identification Project (CCHIP) index. Multinomial logistic regression analyses were conducted on all households (with and without children) to determine the predictors of food insecurity, with additional analyses adjusting for child dependency and sociodemographic characteristics of household heads in households with children. Of 5 098 households surveyed, 68.6% had children and adolescents present (0-19 years). Of these households, 32.5% [95% CI (29.5 – 35.7)] were experiencing hunger and 26.3% [95% CI (23.9 – 28.8)] were at risk of hunger. Among all the households, significant associations for experiencing hunger were the presence of children and adolescents: AOR [95% CI]: 1.68 [1.12 – 2.53]; being female-headed: AOR [95% CI]: 1.53 [1.21 – 1.94] and informally-located; AOR [95% CI]: 1.61 [1.07 – 2.43]. Of the racial groups, having a non-African household head, Coloured: AOR [95% CI]: 0.29 [0.19 – 0.44] and White/Indian/Asian: 0.12 [0.04 – 0.33] conferred lower odds of experiencing hunger; and, the household head having secondary/tertiary education conferred lower odds of experiencing hunger; AOR [95% CI]: 0.40 [0.28 – 0.56] as well as being at risk of hunger; AOR [95% CI]: 0.69 [0.52 – 0.92]. Receiving social grants, pensions, or remittances more than doubled the odds of experiencing hunger; AOR [95% CI]: 2.15 [1.49 – 3.09]. After adjusting for child dependency in

households with children, having at least one older child (age 15-19 years old) did not change the odds of food insecurity. In summary, only 41% of South African households with children and adolescents were food secure. The associations between household head sociodemographics, household location and size on household food insecurity indicate a need for multi-sectoral interventions to bolster sustainable food systems for households with children and adolescents and to improve public protections for female-headed, African-headed and informally-located households dependent on social grants.

Keywords: children, adolescents, household, food insecurity, hunger, CCHIP index, South Africa, SANHANES

Introduction

Food insecurity has emerged as a major public health concern; and, the obligation to realise the "*right to food and basic nutrition"* is enshrined in both the United Nations Charter [1] and the South African Constitution [2]. The Food and Agriculture Organization (FAO) of the United Nations states that "food security exists when all people, at all times, have physical, social and economic access to sufficient, safe and nutritious food that meets their dietary needs and food preferences for an active and healthy life" [3], emphasising that the availability of food must be beyond mere subsistence [4]. In Sub-Saharan Africa, one in four people experienced undernutrition in the year 2017. This represents about one-third of the global population estimated to have suffered from chronic hunger in the same year [5]. According to actuarial projections, Africa will be home to 90% of the world's poorest by 2030 [6].

In 2017, 6.8 million South Africans were affected by hunger, representing a 50% decline since 2002; however, at the household-level, the burden of food insecurity remains largely unabated [7]. Moreover, a considerable but undocumented proportion of these households comprise children and adolescents who are vulnerable to food deprivation and hunger [4]. Indeed, it has been suggested that the presence of children in households may confer additional "*pressure of mouths to feed*" [7'8].

Throughout the world, household food insecurity is associated with malnutrition, especially stunting and wasting among children under five years old [9]. Nationally, Lake et al. [10] reported that approximately half of South African children under age five were malnourished; and, severe-acute and moderate-acute malnutrition accounts for between 4% to 25% of child mortality [10,11]. Such implications of food insecurity on health and nutrition have been observed

not only in children under five, but among older children as well. The largest national census of South African households conducted in 2011/12, the South African National Health and Nutrition Examination Survey (SANHANES), reported that the highest prevalence for wasting among children was actually among boys and girls aged 10–14 years (5.6% and 2.5% respectively); and, underweight was most prevalent among boys and girls in the 7–9 year-old age group (8.6% and 4.0% respectively) [12]. In older children, the South African Demographic and Health Survey (SADHS) showed that 27% and 9% of adolescent girls and boys aged 15-19 years respectively, were overweight or obese [13], highlighting the double burden of malnutrition across childhood and adolescence in South Africa. Importantly, hunger has profound health consequences that not only affect physical growth and cognitive development in childhood [14,15], but endure throughout the life-course into adulthood [16,17] and extend into subsequent generations [18,19]. Two longitudinal cohort studies of the consequences of hunger that began in the throes of the Second World War, the Dutch Winter Famine [19] and the siege of Leningrad [18,20] indicate that the effects of intra-uterine exposure to food insecurity can traverse through multiple generations, predisposing to a “thrifty phenotype” among progeny. Bjerregaard, et al. [21] found that another form of malnutrition, obesity in childhood (measured at ages 7 and 13 years), significantly increased the hazards of developing type-2 diabetes in late adulthood among Danish men. Conversely, reversal of obesity in adolescence reduced the risk by 4%, a finding that may be explained, in part, by food insecurity given that obesity and type-2 diabetes are primarily diet-sensitive adverse health outcomes [15].

In South Africa, the Community Childhood Hunger Identification Project (CCHIP) index was used to estimate the prevalence of food insecurity nationally between 1999 and 2008 in three

different studies: the National Food Consumption Surveys of 1999 and 2005, and the South African Social Attitudes Survey of 2008 [22]. A review of these surveys, which included children from 1-9 years old with at least one adult in the household, indicated a marked decline of household food insecurity from 52.3% in 1999 to 25.9% in 2008 [22]. These studies looked at a segment of children (those 1-9 years old), thus perhaps underestimating the true extent of child and adolescent food insecurity [23,24]. Much of what is known about the sociodemographic correlates of food insecurity in South Africa has been gleaned from sub-national cross-sectional studies. Food insecurity measured using the Household Food Insecurity Access Scale (HFIAS) indicated a gradient across the rural-urban continuum of South African towns, with households in rural areas reporting higher scores, and therefore more food insecurity, than peri-urban households (households located in urban-informal settlements) [25]. Households that are reliant on private food charities and government-assisted social grants are at an increased risk of food insecurity [26]. It is speculated that while social grants are regular and predictable, they are, however, not sensitive to the changing needs of a household [26,27]. The gender of the household head is also an important factor predicting household vulnerabilities [9], with the highest prevalence of food insecurity in South Africa reported among female-headed households [12]. In an analysis of international data, women are more likely to experience food insecurity in comparison to men, which is attributed mainly to disparities in income, education and access to social networks [28]. Even with increased employment rates among women, the income differentials between women and men has meant that poverty remains strongly gendered. Research into possible reasons for the gendered nature of food insecurity in South Africa include decades of migrant labour practices disrupting family structures, and household size [7]. Female-headed households are larger, on

average, than male-headed households and include more dependent children and adolescents, as well as are less likely to have any household member working [11].

There is a paucity of nationally representative data to assess the granular detail about food insecurity across the country. The critical need to address hunger in childhood necessitates particular attention to factors affecting food insecurity in households with children and adolescents, so that nutrition-sensitive interventions can be developed and/or strengthened [29]. Such interventions would address the underlying determinants of child hunger and include, for example, multi-sectoral approaches to social safety netting through minimum guaranteed income and/or cash transfers; financial, educational and parenting support to female-headed households with children; and bolstering health services and sustainable agricultural practices [5,29]. Such multi-pronged approaches are necessary to mediate the short-term, long-term and inter-generational adverse health consequences of food insecurity on childhood health. Therefore, drawing from a large-scale population-based sample of households, the SANHANES-1, we set out to determine the prevalence of food insecurity in households with and without children as well as to assess associations between the sociodemographic characteristics of the household head and degrees of hunger.

Methods

Study design

We conducted a secondary data analysis of the SANHANES-1 [12]. The SANHANES-1 is a cross-sectional nationally representative sample of households that employed a multi-stage disproportionate, stratified cluster sampling approach based on the 2001 census Enumeration Areas (EAs). The first 20 households were sampled from each of the 500 EAs yielding a sample of 10 000 households. Ensuring national representativeness, the SANHANES-1 EA sampling was layered by type of locality (urban/rural; formal/informal), and province (there are nine in South Africa) as well as by "race" in formal urban areas. Using race as a variable allows investigation of ongoing health disparities that have endured post-apartheid. In this way, the SANHANES captures the sociodemographic and economic profile of the country. Additional sample selection details have been published elsewhere [12]. In our study, we used the "household" as the unit of analysis. Statistics South Africa (Stats-SA) defines a household as "a group of people who live together ([…)], eat together and share resources, or a single person who lives alone" [30]. In the SANHANES-1, household membership was attributed to persons who occupied the same dwelling and slept in the household for at least four nights a week. The household head was identified as a physically present member who was designated internally as the head of that household, and also served as the main respondent for household-level data acquisition.

Data extraction and study population

The SANHANES-1 database includes people of all ages residing in households in South Africa. For the present study, only occupied households were included **(S1 Fig)**. Five separate but inter-related SANHANES-1 questionnaires were administered at the household-level by trained field workers capturing information on everyone living in that household (between 1 and 20+ people). The variables of interest for this study were extracted from each of the five questionnaires: visiting point (household) questionnaire (administered to the household head); child questionnaire (0-14 years old); child clinical examination form (0-14 years old); adult questionnaire (administered to anyone ≥ 15 years old); and the adult clinical examination form (≥ 15 years old). After extraction, these data were entered into four separate newly created databases. Children, whom we defined as aged 0-19 years old in order to include the adolescent population in our sample, and adults aged ≥20 years old were extracted from both the child (<15 years) and adult (≥15 years) individual-level datasets, respectively. This age selection aligns with global and national initiatives that target child and adolescent public health and nutrition [31-35]. After merging data for individuals aged 15-19 years with data from those 0-14 years old, thereby reclassifying everyone aged 0-19 years old as "children", we then reconciled them back into their corresponding households. The final sample that we analysed included 5 098 households and their occupants, both adults and children/adolescents. **S1 Fig** illustrates the database management, organisation and integration of the different household and individual-level variables from the five SANHANES-1 databases.

Ethical considerations

The original SANHANES-1 (2011/12) received ethics approval from the Research Ethics Committee (REC) of the HSRC (REC 6/16/11/11). The present secondary analysis received ethics approval from the Human Research Ethics Committee (HREC) (Medical) of the University of the Witwatersrand, Johannesburg, South Africa (Clearance certificate number: M180775).

Variables

Household composition and sociodemographic parameters

At the household-level, the parameters included were household size, the number of adults, the number of children occupying a household and child dependency status. Two variables were created to reflect household dependency status: those with children aged 0-14 years only (yes or no) and those with at least one child 15-19 years old (yes or no), who could theoretically contribute to overall household income given the legal age at which one can start working in South Africa [36]. Age, self-reported sex (male and female), self-reported race (African, Coloured, White/Indian/Asian), educational attainment (no schooling, primary, secondary, tertiary/higher degrees), marital status (married, living together/civil union, never married, widowed, separated/divorced) and the main source of income (salaries/wages, social grants/remittances/pensions, sale of products and services, no income) of all household heads were also included. Locality (urban, rural, formal and informal) was pre-assigned to the household from the EA sampling.

Household food insecurity

Food insecurity was measured using the CCHIP index, a validated tool for the assessment of food insecurity at the household-level [37]. While five of the eight questions are child-referenced, making the CCHIP a specific tool for measuring *childhood* food insecurity, the CCHIP was also administered to households without children, generalising the child-referenced questions to everyone in the household. A score of ≥5 affirmative responses indicate the presence of hunger, understood as "*experiencing hunger*" and recorded as such. A score of 1-4 affirmative responses indicates that the household is "*at risk of hunger*". Lastly, a score of zero indicates that the household is "*food secure*".

Statistical analyses

STATA software, version 16.1 (STATA Institute Inc., College Station, TX, USA) was used for database management and statistical analyses. Results are presented as frequencies and percentages for categorical variables and Median [IQR] or arithmetic Mean ± SD in the case of continuous variables. Comparisons between households with and without children were performed using Mann-Whitney U tests for continuous non-normally distributed data. Categorical variables were compared using Chi-Square tests. All analyses were weighted to offset for the over-allocation of EAs in areas where Indian, Coloured or White racial groups prevailed, to ensure that the minimum required sample size in those minority groups were obtained. All analyses were performed using *svy* function in STATA, incorporating sample weights and stratified cluster sampling design to provide estimates. Weighted prevalence of food security in households with children, stratified by sociodemographic characteristics of the household heads was ascertained from frequencies and percentages; however, age differences in prevalence estimates were

computed from a Kruskal-Wallis test with a Bonferroni *post hoc* correction to adjust for multiple comparisons. To improve statistical power in the regression models, locality was dichotomised to formal vs informal; educational attainment (primary, secondary/tertiary/higher degrees and no schooling); marital status (married, never married, living together/civil unions, and widowed/separated/divorced). Weighted univariable and multivariable multinomial logistic regression analyses with robust standard errors were carried out to determine factors associated with being "at risk of hunger" and "experiencing hunger", where the "food secure" category was used as the referent group for comparison in all households, and separately in households with and without children. In households with children, we conducted additional univariable multinomial logistic regressions to examine associations between food security and child dependency, and between food security and female-headed households. A multivariable multinomial logistic regression was carried out to adjust for all of the sociodemographic factors related to the household. Statistical significance was set at $p<0.05$. Multivariable multinomial logistic regression for "at risk of hunger" and "experiencing hunger" using adjusted odds ratios with their 95% confidence intervals (CI) as measures of association are presented as forest plots, which were produced using GraphPad Prism, version 7.00 for Windows (GraphPad Software, La Jolla, California USA).

Results

Household composition, living arrangements and main source of income of household heads

Table 1 shows the characteristics of South African households with and without children, and the living arrangements of the heads of households. Of the 5 098 households with complete CCHIP scores, 3 499 (68.6%) households contained children aged 0-19 years. Households with children were occupied by 9 505 adults and 9 184 children, while those without children were occupied by 3 243 adults. Households with children were 2.5 times larger than those without children ($p<0.001$). Forty percent of all households in South Africa contained children and were female headed. Of the households containing children, nine were child-headed. When comparing households with and without children, the proportions of African- and Coloured-headed households were not different ($p=0.417$) and, no differences between urban formal and urban informal settings were found following two by two comparisons ($p=0.361$). Furthermore, there was no statistically significant difference in the main source of income for households with and without children ($p=0.381$).

Table 1. Characteristics of occupied South African households with and without children, and sociodemographic characteristics of household heads.

	All Households N=5 098		p value
	With children n=3 499	Without children n=1 599	
**Household characteristics**			
Household size (n=21 932)	5 [4 – 6]	2 [1 – 2]	<0.001[&]
No. of Adults, ≥20 years (n=12 748)	2 [2 – 3]	2 [1 – 2]	<0.001[&]
No. of Children, ≤19 years (n=9 183)	2 [1 – 3]	-	-
**Gender (n=5 092)**			
Male	1 532 (31.77)	899 (15.76)	<0.001[#]
Female	1 963 (40.44)	698 (12.02)	
**Age, years (n=5 093)**			
Mean ± SD	49.22 ± 14.67	52.07 ± 15.91	<0.001[&]
**Race (n=5 090) †**			
African	2 465 (60.88) [a]	849 (19.86)	
Coloured	659 (6.86) [b]	263 (2.48)	<0.001[#]
White/Indian/Asian	373 (4.54) [ab]	481 (5.39)	
**Locality (n=4 889) †**			
Urban formal	1 838 (39.25) [ab]	892 (16.15)	
Urban informal	464 (7.1) [cd]	155 (2.54)	<0.001[#]
Rural formal	359 (4.16) [ace]	222 (6.3)	
Rural informal	838 (22.82) [bde]	121 (1.68)	
**Marital status (n=4 998) †**			
Married	1 678 (34.68) [a]	687 (11.15)	
Living together/civil union	310 (6.1) [bc]	73 (1.28)	
Never married	757 (16.02) [abd]	486 (9.5)	<0.001[#]
Widowed	522 (11.38) [d]	236 (4.07)	
Separated/divorced	156 (3.9) [c]	93 (1.93)	
**Source of income (n=4 608)**			
Salaries or wages	1 330 (29.52)	591 (11.00)	
Social grants/pensions/remittances	1 006 (24.7)	498 (9.49)	0.381
Products and services	138 (3.33)	78 (1.71)	
No income	644 (13.98)	323 (6.27)	

Data presented as median [IQR]; arithmetic mean ± SD and n (%): unweighted (n) and weighted (%).
[&]Denotes p values obtained from Mann-Whitney U tests when comparing households with and without children for continuous variables (household size, number of adults and age of household head).
[#]Denotes p values obtained from overall Chi-Square tests when comparing households with and without children for categorical variables (gender, race, locality, and marital status).
[†]Denotes results from multiple 2x2 comparisons with Bonferroni correction: p<0.05 if categories share the same letter (a, b, c, d, and e) when comparing households with and without children. Specifically, for race and locality, p<0.0001 for African vs White/Indian/Asian, Coloured vs White/Indian/Asian, urban formal vs rural formal, and urban formal vs rural informal. For marital status, p=0.001 in married vs never married and

living together vs never married, p=0.036 in Separated/divorced vs living together/civil unions and lastly p=0.013 in never married vs widowed.

Prevalence of food insecurity in households with children

In households with children, the prevalence of *experiencing hunger* and being *at risk of hunger* was 32.5% [95% CI (29.5 – 35.7)] and 26.3% [95% CI (23.9 – 28.8)], respectively (**Fig 1**). **Table 2** shows the weighted prevalence of food insecurity stratified by the household head sociodemographic characteristics. Households in which the household head was female, older, of African race, unmarried, not having a formal education, dependent on social grants/pensions/remittances and having no income showed a higher prevalence of experiencing hunger or being at risk of hunger (p<0.001). Further analysis of the collapsed locality categories (formal vs informal) yielded the following: 37.2% of households with children were informally located; and, of these, only 28.9% were food secure. In contrast, of the 62.9% of households with children located in formal areas, 53.6% were food secure (p<0.001).

Fig 1. National prevalence of food insecurity in households with children.

Table 2. Weighted prevalence of household food security, stratified by sociodemographic characteristics of designated household heads of South African households with children.

	Total N	Food secure n (%)	†At risk of hunger n (%)	‡Experiencing hunger n (%)	p value[#]
**Gender (n=3 495)**					
Male	1 532	813 (22.16)	356 (11.09) a	363 (10.75) a	<0.001
Female	1 963	739 (19.07)	502 (15.13) a	722 (21.8) a	
**Age, years (n=3 499)**					
Mean ± SD	3 499	47.87±13.85 *	49.76±15.15	50.70±15.25	0.001
**Race (n=3 497)**					
African	2 465	845 (30.18)	662 (23.16) ab	958 (30.89) ab	0.001
Coloured	659	388 (5.85)	155 (22.6) a	116 (1.38) ac	
White/Indian/Asian	373	319 (5.16)	43 (0.85) b	11 (0.27) bc	
**Marital status (n=3 423)**					
Married	1 678	899 (23.43)	375 (12.3) a	404 (12.39) abc	0.001
Living together/civil union	310	112 (3.16)	80 (22.9)	118 (3.01) a	
Never married	757	256 (6.85)	212 (6.25) a	289 (9.13) b	
Widowed	522	196 (5.69)	134 (3.98)	192 (6.12) c	
Separated/divorced	156	61 (1.96)	44 (1.38)	51 (2.07)	
**Educational attainment (n=3 499)**					
Primary	820	243 (6.48)	223 (6.28) a	354 (10.34) ab	<0.001
Secondary	1 524	762 (18.27)	390 (12.11) b	372 (11.49) acd	
Tertiary/higher degree	353	280 (8.4)	50 (1.81) abc	23 (0.81) bce	
No schooling/other	802	268 (8.05)	197 (6.05) c	337 (9.91) de	
**Source of income (n=3 118)**					
Salaries or wages	1 330	774 (22.93)	285 (9.32) ab	271 (9.01) ab	<0.001
Social grants/pensions/remittances	1 006	329 (10.69)	266 (9.34) a	411 (14.5) a	
Sale of products and services	138	71 (2.11)	37 (1.46)	30 (1.08) c	
No income	644	208 (5.54)	174 (5.86) b	262 (8.15) bc	
**Locality (n=3 499)**					
Urban formal	1 838	1 049 (27.85)	410 (12.9) ab	379 (12.77) abc	<0.001
Urban informal	464	131 (2.59)	140 (3.11) a	193 (3.98) a	
Rural formal	359	128 (2.18)	80 (1.19)	151 (2.3) b	
Rural informal	838	245 (8.58)	230 (9.05) b	363 (13.49) c	

[#]p value from overall Chi-Square test when comparing food security status (food secure, at risk of hunger and experiencing hunger) across categorical variables (gender, race, marital status, educational attainment, source of income and locality), of which all were statistically significant. *p<0.001 for age when comparing food secure vs at risk of hunger and food secure vs experiencing hunger (Kruskal-Wallis with Bonferroni correction). On multiple 2 by 2 analyses with Bonferroni correction, statistically significant differences within categories of sociodemographic characteristics are indicated by sharing the same letter: a, b, c, d or e, when comparing food secure vs at risk of hunger and food secure vs experiencing hunger. Specifically, p<0.01 between food secure vs. experiencing hunger except for: ‡ Primary vs no schooling, salaries vs products/services; pensions/grants vs product/services and no income; p<0.01 between food secure vs. at risk of hunger except for: †Coloured vs White/Indian/Asian; primary vs secondary; primary vs no schooling and secondary vs no schooling; salary vs product/services, pension/grants vs product/services, pension/grants vs no income and product /services vs no income; p<0.01 between food secure vs at risk of hunger and food secure vs experiencing hunger for married vs never married and married vs widower; p<0.01 between food secure vs experiencing hunger for married vs living together.

Factors associated with experiencing hunger and being at risk of hunger in all households with and without children

Weighted univariate multinomial logistic regression (**S1 Table**) of all the households exhibits that household heads who were female, of older age, unmarried/widowed/separated vs married were at higher risk of hunger and of experiencing hunger. However, when performing a weighted multivariable multinomial logistic regression (**Figs 2A and 2B**), age was no longer associated with experiencing hunger. Yet, female-headed households had 1.53 times increased odds of experiencing hunger as compared to male-headed households ($p<0.001$). In addition, having a larger household and a household with children present were also predictors of experiencing hunger; AOR [95% CI]: 1.06 [1.00 – 1.21]; 1.68 [1.12 – 2.53] respectively. Having a household head who was either Coloured or White/Indian/Asian as opposed to African conferred lower odds of both experiencing hunger AOR [95% CI]: 0.29 [0.19 – 0.44] and 0.12 [0.04 – 0.33] and being at risk of hunger AOR [95% CI]: 0.54 [0.39 – 0.75], 0.22 [0.12 – 0.40]. Furthermore, secondary/tertiary education conferred lower odds of both experiencing hunger; AOR [95% CI]: 0.40 [0.28 – 0.56] and being at risk of hunger; AOR [95% CI]: 0.69 [0.52 – 0.92]. Households relying on pensions/social grants/remittances, or not having any source of income, were ~1.76 to 2.15 times more at risk of hunger and experiencing hunger than those receiving a salary/wage.

Fig 2A. Factors associated with being at risk of hunger, data presented as adjusted odds ratios (AOR) and 95% Confidence intervals (95% CI).

Reference categories: *Presence of children (no children present), Gender (male), Race (African), Marital status (married), Educational attainment (primary), Source of income (salaries/wages), Locality (formal)*

Fig 2B. Factors associated with experiencing hunger, data presented as adjusted odds ratios (AOR) and 95% Confidence intervals (95% CI).

Reference categories: *Presence of children (no children present), Gender (male), Race (African), Marital status (married), Educational attainment (primary), Source of income (salaries/wages), Locality (formal)*

Female household heads and food security in households with children

Compared to male heads of households with children (**Table 3**), female household heads were older, predominantly African, never married/widowed, have primary/secondary education or no schooling, reside in rural informal settings and have no income or rely on social grants/pensions/remittances. Lastly, male-headed households had more adults in the household while female-headed households had more children of all ages.

Table 3. Sociodemographic characteristics of male and female-headed households with children.

	Total N=3 495	Male headed	Female headed	p value
		n=1 532	n=1 963	
**Age, years (n=3 495)**				
Mean ± SD	3 495	48.43±13.86	49.81±15.24	0.0423
**Race (n=3 493)[&]**				
African	2 462	976 (78.99)	1 486 (88.40)	
Coloured	658	324 (11.73)	334 (7.67)	0.0001
White/Indian/Asian	373	231 (9.29)	142 (3.94)	
**Marital status (n=3 420)[#]**				
Married	1 677	1 078 (71.39)	599 (29.84)	
Living together/civil unions	309	222 (13.94)	87 (4.07)	<0.001
Never married	757	132 (9.09)	625 (32.63)	
Widowed	521	53 (3.58)	468 (25.35)	
Separated/divorced	156	28 (1.99)	128 (8.11)	
**Educational attainment (n=3 495)[^]**				
Primary	820	307 (18.28)	516 (26.96)	
Secondary	1 521	706 (44.03)	815 (40.08)	<0.001
Tertiary/Higher degrees	353	215 (16.24)	138 (6.96)	
No schooling/other	801	388 (21.45)	494 (26.00)	
**Source of income (n=3 116)[$]**				
Salaries or wages	1 328	798 (55.44)	530 (29.45)	
Social grants/pensions/Remittances	1 006	305 (23.66)	701 (43.58)	<0.001
Sale of products and services	138	76 (5.46)	62 (4.00)	
No income	644	225 (15.45)	419 (22.97)	
**Locality (n=3 495)***				
Urban formal	1 836	874 (60.13)	962 (48.31)	
Urban informal	464	178 (8.28)	286 (10.82)	<0.001
Rural formal	359	191 (7.27)	168 (4.44)	
Rural informal	836	289 (24.32)	547 (36.43)	
**Composition, Median [IQR]**				
Household size	3 495	5 [4-6]	5 [3-6]	0.090
No. of adults	3 495	3 [2-4]	2 [2-3]	<0.001
No. of children	3 495	2 [1-3]	2 [1-4]	<0.001
No. of children aged 0-14 years	1814	2 [1-3]	2 [1-3]	<0.001
No. of children aged 15-19 years	1681	2 [2-4]	3 [2-4]	<0.001

&Race: African vs Coloured (p=0.001) and African vs White/Indian/Asian (p=0.001)
#Marital status: married vs never married (p<0.001), married vs widowed (p<0.001), married vs separated/divorced (p<0.001), living together/civil union vs never married (p<0.001), living together/civil union vs widowed (p<0.001), living together/civil union vs separated/divorced (p<0.001), and never married vs widowed (p=0.0015)
^Educational attainment: primary vs secondary (p=0.0002), primary vs tertiary/higher degrees (p<0.001), secondary vs tertiary/higher degrees (p=0.0001), and tertiary vs no schooling (p<0.001)
$Source of income: salaries/wages vs social grants/pensions/remittances (p<0.001), salaries/wages vs no income (p<0.001), and social grants/pensions/remittances vs sale of products and services (p=0.0002)
*Locality: urban formal vs urban informal (p=0.001), urban formal vs rural informal (p<0.001), urban informal vs rural informal (p=0.0010), and rural formal vs rural informal (p<0.001).

In the univariate analysis of the gender of the household head and food security in households with children, female-headed households had greater odds of being at risk of hunger [OR 1.58; 95% CI: 1.22-2.04, p<0.001] and experiencing hunger [OR 2.35; 95% CI: 1.86-2.98, p<0.001].

Child dependency and sociodemographic characteristics of household heads, in households with children

In the univariate analysis of child dependency and food security in households with children, having at least one child who was 15 years or older increased the odds of experiencing hunger by 28% (OR 1.28; 95% CI: 1.06-1.56, p=0.012). **Table 4** shows associations between food security and child dependency, adjusted for the sociodemographic characteristics of the household heads. Compared to households with younger children only, having at least one child aged 15-19 years old in a household was not associated with either being at risk of hunger or experiencing hunger (no significant change in adjusted odds ratio). Having a household head who was male, Coloured/White/Indian/Asian, employed and having obtained tertiary education/higher degrees conferred lower odds of experiencing hunger. Residing in informal settings increased the odds of both being at risk of hunger and experiencing hunger.

Table 4: Weighted multivariable multinomial regression analysis between food security and child dependency, adjusted for sociodemographic characteristics of the heads of households with children.

Characteristics	At risk of hunger		p value	Experiencing hunger		p value
	AOR	95% CI		AOR	95% CI	
Household size	0.98	0.92 – 1.04	0.604	1.04	0.99 – 1.10	0.102
**Child dependency**						
Children aged 0-14 years only	***REF***	***REF***	***REF***	***REF***	***REF***	***REF***
At least one child aged 15-19 years	1.01	0.78 – 1.30	0.934	1.24	0.95 – 1.62	0.105
**Age, years**						
Age	0.99	0.98 – 1.01	0.336	0.98	0.97 – 1.00	0.089
**Gender**						
Female	***REF***	***REF***	***REF***	***REF***	***REF***	***REF***
Male	0.92	0.66 – 1.27	0.603	0.72	0.53 – 0.98	0.041
**Race**						
African	***REF***	***REF***	***REF***	***REF***	***REF***	***REF***
Coloured	0.60	0.42 – 0.87	0.006	0.33	0.21 – 0.50	<0.001
White/Indian/Asian	0.17	0.09 – 0.34	<0.001	0.12	0.04 – 0.34	<0.001
**Marital status**						
Living together/civil union	***REF***	***REF***	***REF***	***REF***	***REF***	***REF***
Married	0.88	0.59 – 1.32	0.552	0.61	0.39 – 0.94	0.027
Never married	0.88	0.51 – 1.15	0.652	0.83	0.51 – 1.36	0.478
Widowed	0.67	0.37 – 1.19	0.172	0.63	0.35 – 1.12	0.117
Separated/divorced	1.28	0.59 – 2.75	0.526	1.83	0.54 – 2.56	0.673
**Educational attainment**						
No schooling/other	***REF***	***REF***	***REF***	***REF***	***REF***	***REF***
Primary	1.23	0.82 – 1.83	0.310	1.37	0.94 – 1.99	0.095
Secondary	1.06	0.72 – 1.58	0.742	0.70	0.46 – 1.05	0.086
Tertiary/Higher degree	0.50	0.28 – 0.89	0.019	0.18	0.09 – 0.34	<0.001
**Source of income**						
No income	***REF***	***REF***	***REF***	***REF***	***REF***	***REF***
Pensions/Grants/Remittances	0.91	0.60 – 1.36	0.651	0.82	0.55 – 1.21	0.331
Sale of products and services	0.73	0.40 – 1.34	0.323	0.40	0.19 – 0.81	0.011
Salaries and/or wages	0.51	0.34 – 0.77	0.001	0.38	0.25 – 0.57	<0.001
**Locality**						
Formal	***REF***	***REF***	***REF***	***REF***	***REF***	***REF***
Informal	1.57	1.15 – 2.15	0.004	1.66	1.18 – 2.34	0.003

Discussion

To the best of our knowledge, the present study is the first to objectively quantify the burden of food insecurity among children and adolescents in South Africa and draw associations using a large-scale, population-based sample of households with children. No studies in South Africa have disaggregated households with and without children aged 0-19 years old thus possibly underestimating the true extent of childhood food insecurity and limiting direct comparisons to the present study. Indeed, there is an observed increase in food insecurity (experiencing hunger—25.9% to 32.5%) by 6.6% between the prevalence estimate reported for 2008 by Labadarios et al. [22] and the estimate generated in the present study. This lack of improvement may be accounted for by the enduring socio-political and economic challenges sustained since the end of apartheid [22,38]. Drawing from a cross-sectional, population-based national sample of South African households, we have shown that the prevalence of food insecurity in households with children was nearly 60%. This is similar to the original SANHANES study where food insecurity among all households was 54% [12]. In the present study, after adjusting for household size, the sociodemographic characteristics of the household head and the main source of income, the presence of children and adolescents conferred ~1.68 increased odds of food insecurity (experiencing hunger) relative to households without children. In addition, having a female vs male household head, having an African household head compared to all other race groups, living in informal settings vs formal settings and having no income compared to relying on salaries or wages increased the odds of experiencing hunger between 53% and 300%. Secondary/tertiary/higher educational attainment of the household head reduced the odds of experiencing hunger by at least half relative to primary education. Reliance on social grants, pensions and remittances as the main source of income was associated with more than double the

odds of being food insecure. When analysing food security in households with at least one adolescent aged 15-19 years, versus those with children aged 0-14 years only, before adjusting for the sociodemographics of the household head and location, the odds of being food insecure were almost 30% higher compared to households with younger children only. However, after controlling for these factors, neither child dependency nor the ability to work were associated with any category of food security.

Household head sociodemographics and household characteristics

The majority of households with children and adolescents in our sample was headed by women, had African household heads, or were living in urban or rural informal areas. Therefore, the increased vulnerability of households with children may be explained, in part, by the additive effects of these known predictors of food insecurity [39]. Previous studies in other contexts have found that the presence of children in households is independently associated with food insecurity, beyond sociodemographic characteristics and measures of socioeconomic status [7,39-43]. Additional research has demonstrated that the sex of the household head is an important factor predicting household vulnerabilities, with the highest prevalence of hunger reported among female-headed households [27], a risk that worsens with rural location [25,44]. Our comparative analysis of female- versus male-headed households points to the stark sociodemographic disadvantages that women heads endure in households with children, confirming previous research in South Africa on the gendered nature of poverty and food insecurity [27,45]. In their large-scale, population-based longitudinal analysis, the Indonesian Family Life Survey, Vaezghasemi et al.

[46] further argue that although women may be conscious of healthy food options, because they are at the bottom of the family hierarchy and have low social capital, they remain defenseless against household hunger. Given South Africa's colonial and apartheid history, racial differences still persist in the prevalence of food insecurity, with black or African households bearing the greatest burden compared to historically more advantaged minority groups in the country [7,11,12,25].

Previous studies have demonstrated strong positive associations between the lowest levels of educational attainment and food insecurity, which can be mitigated by higher levels of educational attainment [5,47-50]. Likewise, we found that improved levels of education (secondary and tertiary educational attainment) reduced the risk of food insecurity by at least half; yet, having no formal schooling was not associated with food insecurity. As Chakona and Shackleton also found [25], we suggest that household heads without a formal education may rely on subsistence farming or implement other skills-based practices which might act as a safety net against hunger.

The Born in Bradford cohort, a longitudinal study in the United Kingdom, showed associations between cohabitation status and food insecurity, where pregnant women not living with a partner had a two-fold increased risk of food insecurity [51]. Hanson, Sobal, and Frongillo [52] further demonstrated that marital status was associated with food insecurity among men, but not women. We found significant associations between the marital status of the household head and risk of experiencing hunger. Even after adjustments for sex, living together/civil unions and never married increased the odds of experiencing hunger by 61% and 41%, respectively.

Main source of household income, social grants and child dependency

Using self-reported main source of income to indicate a household's economic stability, we note that there were no differences in income sources between households with and without children. However, household size was larger in households with children, including an increased number of adults. We show that not having any source of income predicts that a household will experience a three-fold increase in experiencing hunger. Furthermore, similar to Ruiters and Wildschutt [27], receiving government social grants, pensions or remittances did not alleviate hunger nor the risk of being food insecure for households with and without children. Despite a substantial social safety net wherein 16.2 million South Africans in 2013, representing nearly one third of the country's population received different types of government grants [53], these were not protective in households with children, with a 76% increase in being at risk of food insecurity and double the odds for being food insecure. Other contexts show mixed results for government assistance programmes on alleviating food insecurity. Tarasuk, Fafard St-Germain and Mitchell. [54] have shown that in Canada, household before-tax income adjusted for family size was protective against food insecurity; however, households receiving social assistance were three times more likely to be food insecure. In contrast, Brown and Tarasuk. [55] showed a significant decline in severe food insecurity subsequent to the roll-out of the Canada Child Benefit (CCB) in households with children, a country-wide non-means-tested cash transfer programme. In Brazil, participation in the Bolsa Familia Programme [56] and in the United States receiving Supplemental Nutrition Assistance Program (SNAP, formerly the Food Stamp Program) support [8,57], were not effective in preventing food insecurity. In one South African study where social grants did show

improvements in household food insecurity and dietary quality, the authors note that these findings were not accompanied by concomitant improvements in anthropometric indicators of child malnutrition [26]. Even when controlling for the sociodemographic characteristics of the household head and the main source of income, the risk of experiencing hunger was increased with the presence of children in all households. This is similar to the findings of a 2017 national study comparing households with and without children under the age of 5 years, whereby households with no children were less likely to experience inadequate access to food [7]. In our sample of households with children, controlling for child dependency status (ages 0-14 versus 15-19 years old) did not lessen the odds of experiencing hunger. Regarding an older child's potential to contribute towards household income through work eligibility, Stats-SA reported in 2012, the year of the SANHANES-1, an overall youth unemployment rate of 35.8% (age 15-34 years old) as compared to an adult unemployment rate of 15.1% [53]. After disaggregating youth unemployment by age and sex, males and females aged 15-19 years had rates of 50.2% versus 74% respectively [53], indicating that child dependency on an adult household head extends throughout adolescence in South Africa.

Contemporary considerations and current context

The present COVID-19 pandemic, occurring alongside household and child food and nutrition insecurity, presents a disconcerting syndemic [58] that is capable of reversing any recent mitigation of hunger at household and individual levels in the country [59]. Indeed, the COVID-19 pandemic has forced South Africa into an abrupt and prolonged lockdown threatening job and societal security [59]. Data from the National Income Dynamics Study-Coronavirus Rapid Mobile Survey (NID-CRAMS) [60], a four-wave longitudinal survey of adult South Africans, indicate that

even with re-opening of the economy, lifting restrictions on movement and returning to work, 37-47% households did not have sufficient money to buy food, with 16-22% stating that they experienced hunger, during the period from May/June 2020 to February/March 2021 [60]. Overall, while hunger declined somewhat over this period, the poorest quintile suffered more hunger than before [60]. Without augmented social supports for households with children and adolescents, and an emphasis on nutrition-sensitive interventions [29,50,61], it is likely that food insecurity will pervade [14] and possibly impede the country from realising its 2030 Sustainable Development Goal of achieving zero hunger [31,58].

Limitations

The present study is not without limitations. The SANHANES-1 was conducted in 2011/12. Although never repeated, the data are at least nine years old and may not reflect the current status of food security in South Africa. Nonetheless, the SANHANES provides the most recent and comprehensive dataset on food security at the national and household-level using the CCHIP index that explores the context and loci within which food insecurity exists. This is in contrast with other national surveys which only record the frequency of problems satisfying food needs "in the past 12 months" [13]. Secondly, a comprehensive assessment of household socioeconomic status was beyond the scope of this research. Having considered the primary source of household income and educational status as contributing factors to household economic stability, a more robust measure of socioeconomic status using household assets could add a more granular dimension to the analysis.

Conclusion

Our study confirms that South African children and adolescents face a major challenge of food insecurity that is driven by a number of household head characteristics and household location. The untoward effects of food insecurity on child and adolescent health are not evanescent and have far-reaching adverse health outcomes. Therefore, this is a clarion call to bolster multi-sectoral interventions and healthy public policies, with a strong focus on the social determinants of health and poverty eradication strategies targeting these vulnerable households.

Acknowledgements

The authors acknowledge Dr Innocent Maposa for his assistance with data analysis.

References

1. United Nations Office of the High Commissioner for Human Rights. Fact Sheet No. 34, The Right to Adequate Food [Internet]. Refworld. 2021 [cited 2021 Jun 7]. Available from: https://www.refworld.org/docid/4ca460b02.html.
2. Food and Agriculture Organization. The Right to Food around the Globe [Internet]. Food and Agriculture Organization of the United Nations. 2015 [cited 2021 Jun 6]. Available from: http://www.fao.org/right-to-food-around-the-globe/countries/zaf/en/.
3. Skoet J, Stamoulis KG. The state of food insecurity in the world 2006 [Internet]. Food and Agriculture Organization. 2006 [cited 2021 Jun 7]. Available from: http://www.fao.org/3/a0750e/a0750e00.pdf.
4. Neuman N, Eli K, Nowicka P. Feeding the extended family: gender, generation, and socioeconomic disadvantage in food provision to children. Food, Cult Soc [Internet]. 2019; 22(1):45–62. https://doi.org/10.1080/15528014.2018.1547066.
5. Fraval S, Hammond J, Bogard JR, Ng'endo M, van Etten J, Herrero M, et al. Food access deficiencies in sub-Saharan Africa: prevalence and implications for agricultural interventions. Front. Sustain. Food Syst. 2019 Nov 19; 3:104. https://doi.org/10.3389/fsufs.2019.00104.
6. The World Bank Annual Report 2019: Ending Poverty, Investing in Opportunity [Internet]. Washington, DC: World Bank. 2019 [cited 2021 Jun 7]. Available from: https://openknowledge.worldbank.org/handle/10986/32333.
7. Towards measuring food security in South Africa: An examination of hunger and food inadequacy [Internet]. Stats-SA. 2019. [cited 2021 Jun 7]. Available from: http://www.statssa.gov.za/publications/03-00-14/03-00-142017.pdf.

8. Nord M. Food Insecurity in Households with Children: Prevalence, Severity, and Household Characteristics. Economic Information Bulletin Number 56. US Department of Agriculture. 2009 Sep. http://www.usda.gov/wps/portal/usdahome.
9. Webb P, Stordalen GA, Singh S, Wijesinha-Bettoni R, Shetty P, Lartey A. Hunger and malnutrition in the 21st century. BMJ [Internet]. 2018 Jun 13 [cited 2021 Jun 14]. doi: https://doi.org/10.1136/bmj.k2238
10. Lake L, Shung-King M, Hendricks M, Heywood M, Nannan N, Laubscher R, et al. Prioritising child and adolescent health: A human rights imperative. In: Shung-King M, Lake L, Sanders D, Hendricks M (eds). South African Child Gauge 2019: Child and adolescent health 2019. Cape Town: Children's Institute, University of Cape Town. p.32-62. ISBN: 978-0-620-85838-0.
11. Hall K, Nannan N, Sambu W. Child health. In: Hall K, Richter L, Mokomane Z, Lake L (eds). South African Child Gauge 2018: Children, Families, and the State 2018. Cape Town: Children's Institute, University of Cape Town. p.144-149. ISBN: 978-0-620-81934-3.
12. Shisana O, Labadarios D, Rehle T, Simbayi L, Zuma K, Dhansay A, et al. South African National Health and Nutrition Examination Survey, 2012 [Internet]. Human Sciences Research Council. 2013 [cited 2021 Jun 7]. Available at: ISBN (e-pub) 978-0-7969-2478-0.
13. South Africa Demographic and Health Survey 2016 Key Findings [Internet]. Available from: https://dhsprogram.com/pubs/pdf/SR248/SR248.pdf.
14. Perez-Escamilla F, de Toledo Vianna RP. Food Insecurity and the Behavioral and Intellectual Development of Children: A Review of the Evidence. J. Appl. Res. Child.

2012; 3(1):9. Available at: http://digitalcommons.library.tmc.edu/childrenatrisk/vol3/iss1/9.

15. Seligman HK, Laraia BA, Kushel MB. Food insecurity is associated with chronic disease among low-income NHANES participants. J. Nutr. 2010 Feb 1; 140(2):304-10. https://doi.org/10.3945/jn.109.112573.

16. Kirkpatrick SI, McIntyre L, Potestio ML. Child hunger and long-term adverse consequences for health. Arch. pediatr. adolesc. med. 2010 Aug 1; 164(8):754-762. doi:10.1001/archpediatrics.2010.117.

17. Ke J, Ford-Jones EL. Food insecurity and hunger: A review of the effects on children's health and behaviour. Paediatr Child Health. 2015 Mar 1; 20(2):89-91. doi.org/10.1093/pch/20.2.89.

18. Sparén P, Vågerö D, Shestov DB, Plavinskaja S, Parfenova N, Hoptiar V, et al. Long term mortality after severe starvation during the siege of Leningrad: prospective cohort study. BMJ. 2004 Jan 1; 328(7430):11. https:// doi.org/10.1136/bmj.37942.603970.9A.

19. Lumey LH, Stein AD, Kahn HS, Van der Pal-de Bruin KM, Blauw GJ, Zybert PA, et al. Cohort profile: the Dutch Hunger Winter families study. Int. J. Epidemiol. 2007 Dec 1; 36(6):1196-204. https://doi.org/10.1093/ije/dym126.

20. Calkins K, Devaskar SU. Fetal origins of adult disease. Curr Probl Pediatr Adolesc Health Care. 2011 Jul 1; 41(6):158-76. https://doi.org/10.1016/j.cppeds.2011.01.001.

21. Bjerregaard LG, Jensen BW, Ängquist L, Osler M, Sørensen TI, Baker JL. Change in overweight from childhood to early adulthood and risk of type 2 diabetes. NEJM. 2018 Apr 4. doi: 10.1056/NEJMoa1713231.

22. Labadarios D, Mchiza ZJ, Steyn NP, Gericke G, Maunder EM, Davids YD, et al. Food security in South Africa: a review of national surveys. Bull. World Health Organ. 2011; 89:891-9. doi: 10.2471/BLT.11.089243.

23. Labadarios D, Steyn NP, Maunder E, Macintryre U, Gericke G, Swart R, et al. The national food consumption survey (NFCS): South Africa, 1999. Public Health Nutr. 2005 Aug; 8(5):533-43. https://doi.org/10.1079/PHN2005816.

24. Labadarios D, Swart R, Maunder EM, Kruger HS, Gericke GJ, Kuzwayo PM, et al. Executive summary of the National Food consumption Survey Fortification Baseline (NFCS-FB-I). S Afr J Clin Nutr. 2008; 21(3 Suppl 2): 247–300.

25. Chakona G, Shackleton CM. Household food insecurity along an agro-ecological gradient influences children's nutritional status in South Africa. Front. Nutr. 2018 Jan 22; 4:72. https://doi.org/10.3389/fnut.2017.00072.

26. Waidler J, Devereux S. Social grants, remittances, and food security: does the source of income matter? Food Secur. 2019 Jun; 11(3):679-702. https://doi.org/10.1007/s12571-019-00918-x.

27. Ruiters M, Wildschutt A. Food insecurity in South Africa: Where does gender matter?. Agenda. 2010 Jan 1; 24(86):8-24. doi: 10.1080/10130950.2010.10540516.

28. Broussard NH. What explains gender differences in food insecurity? Food Policy. 2019 Feb 1; 83:180-94. https://doi.org/10.1016/j.foodpol.2019.01.003.

29. Ruel MT, Alderman H, Maternal and Child Nutrition Study Group. Nutrition-sensitive interventions and programmes: how can they help to accelerate progress in improving maternal and child nutrition? Lancet. 2013 Aug 10; 382(9891):536-51. https://doi.org/10.1016/S0140-6736(13)60843-0.

30. Statistics South Africa. General Household Survey 2017 [Internet]. Stats-SA. 2018 [cited 2021 Dec 31]. Available from: https://www.statssa.gov.za/publications/P0318/P03182018.pdf

31. Pérez-Escamilla R. Food security and the 2015–2030 sustainable development goals: From human to planetary health: Perspectives and opinions. Curr Dev Nutr. 2017 Jul 1; 1(7):e000513. https://doi.org/10.3945/cdn.117.000513.

32. Onis MD, Onyango AW, Borghi E, Siyam A, Nishida C, Siekmann J. Development of a WHO growth reference for school-aged children and adolescents. Bull. World Health Organ. 2007; 85:660-7. doi: 10.2471/blt.07.043497.

33. Republic of South Africa—National Planning Commission. National Development Plan 2030: Our future-make it work. ISBN: 978-0-621-41180-5

34. Kuruvilla S, Bustreo F, Kuo T, Mishra CK, Taylor K, Fogstad H, et al. The Global strategy for women's, children's and adolescents' health (2016–2030): a roadmap based on evidence and country experience. Bull. World Health Organ. 2016 May 1; 94(5):398. doi: 10.2471/BLT.16.170431.

35. Dick B, Ferguson BJ. Health for the world's adolescents: a second chance in the second decade. J Adolesc Health. 2015 Jan 1; 56(1):3-6. https://doi.org/10.1016/j.jadohealth.2014.10.260.

36. Statistics SA. National and provincial labour market: Youth. Statistics South Africa, Pretoria. 2015. https://www.statssa.gov.za/publications/P02114.2/P02114.22015.pdf

37. Swindale A, Bilinsky P. Development of a universally applicable household food insecurity measurement tool: process, current status, and outstanding issues. J. Nutr. 2006 May 1; 136(5):1449S-52S. https://doi.org/10.1093/jn/136.5.1449S.

38. Drimie S, Ruysenaar S. The integrated food security strategy of South Africa: an institutional analysis. Agrekon. 2010 Sep 1;49(3):316-37. https://doi.org/10.1080/03031853.2010.503377.

39. Grobler WC. Perceptions of poverty: a study of food secure and food insecure households in an urban area in South Africa. Procedia Economics and Finance. 2016 Jan 1; 35:224-31. https://doi.org/10.1016/S2212-5671(16)00028-9

40. Chinnakali P, Upadhyay RP, Shokeen D, Singh K, Kaur M, Singh AK, et al. Prevalence of household-level food insecurity and its determinants in an urban resettlement colony in north India. J Health Popul Nutr. 2014 Jun;32(2):227. PMID: 25076660.

41. Mortazavi Z, Dorosty AR, Eshraghian MR, Ghaffari M, Ansari-Moghaddam A, Mohammadi M. Household food insecurity in Southeastern Iran: severity and related factors. Int. J. Food Sci. 2017 Nov 15;2017. https://doi.org/10.1155/2017/7536024.

42. Tantu AT, Gamebo TD, Sheno BK, Kabalo MY. Household food insecurity and associated factors among households in Wolaita Sodo town, 2015. Agric. Food Secur. 2017 Dec; 6(1):1-8. https://doi.org/10.1186/s40066-017-0098-4.

43. Sisha TA. Household level food insecurity assessment: Evidence from panel data, Ethiopia. Sci. Afr. 2020 Mar 1; 7:e00262. https://doi.org/10.1016/j.sciaf.2019.e00262.

44. Hossain M, Choudhury N, Abdullah KA, Mondal P, Jackson AA, Walson J, et al. Evidence-based approaches to childhood stunting in low and middle income countries: a systematic review. Arch. Dis. Child. 2017 Oct 1; 102(10):903-9. http://dx.doi.org/10.1136/archdischild-2016-311050.

45. Molestane R, Reddy V. The gendered dimensions of food security in South Africa: A review of the literature. Policy Brief, Human Sciences Research Council, Pretoria. 2011. Available at: http://hdl.handle.net/20.500.11910/4887

46. Vaezghasemi M, Öhman A, Eriksson M, Hakimi M, Weinehall L, Kusnanto H, et al. The effect of gender and social capital on the dual burden of malnutrition: a multilevel study in Indonesia. PloS one. 2014 Aug 25; 9(8):e103849. https://doi.org/10.1371/journal.pone.0103849.

47. Kim J, Lim H. Nutritional management in childhood obesity. J. obes. metab. syndr. 2019 Dec; 28(4):225. doi: 10.7570/jomes.2019.28.4.225.

48. Gebremedhin S. Prevalence and differentials of overweight and obesity in preschool children in Sub-Saharan Africa. BMJ open. 2015 Dec 1; 5(12):e009005. http://dx.doi.org/10.1136/bmjopen-2015-009005.

49. Devereux S, Hochfeld T, Karriem A, Mensah C, Morahanye M, Msimango T, et al. Food Security SA Working Paper Series: #004 School Feeding in South Africa: What we know, what we don’t know, what we need to know, what we need to do Authors [Internet]. ; Available from: https://foodsecurity.ac.za/wp-content/uploads/2018/06/CoE-FS-WP4-School-Feeding-in-South-Africa-11-jun-18.pdf.

50. Bhutta ZA, Das JK, Rizvi A, Gaffey MF, Walker N, Horton S, et al. Evidence-based interventions for improvement of maternal and child nutrition: what can be done and at what cost? Lancet. 2013 Aug 3; 382(9890):452-77. https://doi.org/10.1016/S0140-6736(13)60996-4.

51. Power M, Uphoff EP, Stewart-Knox B, Small N, Doherty B, Pickett KE. Food insecurity and socio-demographic characteristics in two UK ethnic groups: an analysis of women in

the Born in Bradford cohort. J Public Health. 2018 Mar 1; 40(1):32-40. https://doi.org/10.1093/pubmed/fdx029.

52. Hanson KL, Sobal J, Frongillo EA. Gender and marital status clarify associations between food insecurity and body weight. J Nutr. 2007 Jun 1; 137(6):1460-5. https://doi.org/10.1093/jn/137.6.1460.

53. Cassim A, Morne Oosthuizen. The State of Youth Unemployment in South Africa [Internet]. Brookings. Brookings; 2014 [cited 2021 Dec 26]. Available from: https://www.brookings.edu/blog/africa-in-focus/2014/08/15/the-state-of-youth-unemployment-in-south-africa/

54. Tarasuk V, St-Germain AA, Mitchell A. Geographic and socio-demographic predictors of household food insecurity in Canada, 2011–12. BMC public health. 2019 Dec; 19(1):1-2. https://doi.org/10.1186/s12889-018-6344-2.

55. Brown EM, Tarasuk V. Money speaks: Reductions in severe food insecurity follow the Canada Child Benefit. Prev. Med. 2019 Dec 1; 129:105876. https://doi.org/10.1016/j.ypmed.2019.105876.

56. Costa NS, Santos MO, Carvalho CP, Assunção ML, Ferreira HS. Prevalence and factors associated with food insecurity in the context of the economic crisis in Brazil. Curr Dev Nutr. 2017 Oct 1; 1(10):e000869. https://doi.org/10.3945/cdn.117.000869.

57. Coleman-Jensen A, Gregory C, Singh A. Household food security in the United States in 2013. USDA-ERS Economic Research Report. 2014 Sep 1(173). http://dx.doi.org/10.2139/ssrn.2504067.

58. Pérez- Escamilla R, Cunningham K, Moran VH. COVID- 19 and maternal and child food and nutrition insecurity: a complex syndemic. Matern. Child Nutr. 2020 Jul; 16(3):e13036. https://doi.org/10.1111/mcn.13036.

59. International Labour Organization. ILO Monitor: COVID-19 and the World of Work. Updated estimates and analysis. Int Labour Organ. 2020 Apr 29. Available from: https://www.ilo.org/global/about-the-ilo/WCMS_738753/lang--en/index.htm.

60. Patel L, Bridgman G. Hunger in South Africa during 2020: Results from Wave 3 of NIDS-CRAM [Internet]. CRAM. 2021 [cited 2021 Jun 7]. Available from: https://cramsurvey.org/wp-content/uploads/2021/02/10.-Van-der-Berg-S.-Patel-L.-Bridgman-G.-2021-Hunger-in-South-Africa-during-2020-Results-from-Wave-3-of-NIDS-CRAM-1.pdf.

61. Bhutta ZA, Das JK, Rizvi A, Gaffey MF, Walker N, Horton S, et al. Maternal and Child Nutrition Study Group. Evidence-based interventions for improvement of maternal and child nutrition: what can be done and at what cost? Lancet. 2013 Aug 3; 382(9890):452-77. https://doi.org/10.1016/S0140-6736(13)60996-4.

Supporting information

S1 Fig. Database management, organisation and integration of data extracted from the four household and individual - level datasets of the SANHANES-1

S1 Table. Weighted univariable multinomial logistic regression analysis of factors associated with being at risk of hunger and experiencing hunger against being food secure in households with and without children

S1 Dataset. Final analytic sample of households with food security categories

RESPONSE TO REVIEWERS – 2022/05/12 Revision required [PONE-D-20-39991R2] - [EMID:61e94e729c497602]

Reviewer 2	1. Recommendation for publication: *No response*<br>2. Technically sound and data supporting conclusions: *Partly*<br>3. Review Comments to the Author: *Some of these revisions have, indeed, improved this manuscript - particularly in incorporating Table 3 and Table 4. Unfortunately, my underlying concerns remain.*	Reviewer 2 acknowledges improvement in the manuscript following the additional analyses and reporting of results in Tables 3 and 4. However, Reviewer 2 has persistent concerns.
Reviewer 3	*Please check L-187, a reference is missing, and please add the reference*	No reference is missing here. An appropriate reference was provided for the direct quote. Rather, we are indicating partially deleted text from a direct quotation. To avoid confusion and conform to the Chicago Style Manual, we have now changed this from brackets with three dots [...] to an ellipsis with three dots (. . .) to signal that part of the text has not been included in the quotation.
