# Supplementary material for: Child and adolescent food insecurity in South Africa: A household-level analysis of hunger
Source: PLoS One. 2022 Dec 28;17(12):e0278191. doi: 10.1371/journal.pone.0278191 (PMC9797094; doi:10.1371/journal.pone.0278191)
Supplement: Attachment — Submitted filename: Response to Reviewers_01 October 2022.pdf [file pone.0278191.s011.pdf]

RESPONSE TO REVIEWERS – 2022/10/02 Revision required [PONE-D-20-39991R3] - [EMID:5e43ac9b00b617d8]

**Reviewers' comments**	**Authors' response**
Reviewer #3<br><br>*Excellent effort to respond to all the comments has improved the manuscript tremendously. Please see the minor edits and comments inserted in the manuscript for your consideration.*	Line 190: These three dots are called an ellipsis, indicating "the omission of a word, phrase, line, paragraph, or more..." from a direct quotation. We have represented the words left out of the SANHANES definition of a household with three dots and spaces either side. This is consistent with both *The Chicago Manual of Style, 17th ed.* (Chicago: University of Chicago Press, 2017), 13.50*.,* and the *MLA Handbook*, 8th ed. (New York: Modern Language Association of America, 2016), 85. We have removed the parentheses.<br><br>Line 221: HSRC is now spelled out with first time use in the manuscript (Human Sciences Research Council)<br><br>Line 239: The CCHIP Index is spelled out in full with first time use in Line 132 of the manuscript
Reviewer #4<br><br>*Thank you for the opportunity I have to provide a review for this important article on Childhood food insecurity in South Africa. The authors have done a good job of identifying the extent of childhood hunger and factors which contribute to it.*<br><br>*Title*<br>*• The title clearly depicts the research that was carried out with focus on the outcome, however the period or time of investigation is missing*	- We do not specify the dates of the original SANHANES study (2011-2012) or when we undertook the secondary analysis (2019-2020) in the title of the manuscript. We have, however, now indicated these timelines in the Methods section to ensure greater clarity.

• Each author’s contribution was clearly stated, and corresponding author’s contact is clear.<br><br>*Abstract*<br>*• The abstract is unstructured, and it allowed for a good read. It is a good summary of the work done.*<br><br>*Introduction*<br>*• The introduction revealed the intent of the study and raise curiosity as to what could be the finding of the study.*<br>*• However, the authors used very old references-I think they could use more recent references. I could not relate well with when the study was conducted because I could not find anywhere it was mentioned. I could have missed it.*<br>*• The objectives were well stated: The authors wanted to determine the prevalence of food insecurity in households with and without children as well as to assess associations between the sociodemographic characteristics of the household head and degrees of hunger.*<br><br>*Methods and Materials*<br>*• The methods were well explained, and it is repeatable.*<br>*• The SANHANES-1 database was used for this study, however the question is when was the data extracted or when was this study conducted?*<br>*• Ethical consideration should have been the last in method. Authors can consider making the changes if*	- We have updated the references as requested; however, many of the “older” references provide an historical context to this work.<br>- In Methods, we clarified when the SANHANES-1 data were collected originally, and when we undertook this data extraction and conducted our analyses.<br><br>- We clarified when the original SANHANES-1 study was conducted, as well as when the secondary data analysis was undertaken.<br>- The Ethical Considerations sub-section has been moved to the end of Methods.

they want.<br><br>*Results*<br>*• Well written results and clearly stated*<br>*• In lines 354-6 – larger household as a predictor of experiencing hunger seems not to be related. LCL of 1.00 shows that if repeated a researcher can get AOR of 1.00. I think 4 digits LCL should be used if not it should not be reported because it is an unlikely association.*<br><br>*Discussion*<br>*• The discussion is well written and provides the necessary reasoning behind the results of the study*<br>*• It is was nice that they provided sub-headings which made an easy understanding of the discussion.*<br>*• The limitation of the study was clearly stated which eventually clarifies some of my earlier questions on when this study was conducted. But I think the authors should clearly state the year of study in the title.*<br><br>*Conclusion*<br>*• Provides answers to the objectives of the study*<br>*• The recommendations made by the authors may not be relevant since the authors agreed that this is a very old data, but the only data available for this kind of secondary data analysis.*	- As regards the association between household size and experiencing hunger, we re-ran the analysis<br>- The LCL was 1.009218, which we've now rounded up to 1.01, and updated both the manuscript (Line 485) and the Fig2B to reflect these changes.<br><br>- We have clarified earlier in the manuscript when the original SANHANES-1 data collection happened, as well as when we undertook the secondary analysis.<br><br>- Until the SANHANES-2 is conducted, these data provide the only baseline for food insecurity in South African households with children and adolescents.

Recommendation<br>*• Accept with minor revision*	
Reviewer #5<br><br>*It is an article with clear objective and met it if the method of analysis used was correct.*<br>*But I doubt on the selection of method for analysis. Is it difficult to dichotomize the outcome variable? If so, why you did not use another method of data analysis? Because, multinomial logistic regression is usually not preferred except when the the outcome variable is difficult to dichotomize or the categories of the outcome variable is worth as it is or multinomial.*	- The main outcome indicator for our study was the degree of household food insecurity as measured by the Community Childhood Hunger Identification Project (CCHIP) Index. The index has been internationally validated according to a particular scale with three different outcome measures: 'food secure', 'at risk of hunger' and 'experiencing hunger'. These three categories are standard, so it would not possible to re-categorise the score as dichotomous.
Reviewer #6<br><br>*¬ The title is better to be "prevalence of food in security and associated factors among households in South Africa" Or "Childhood and adolescent food insecurity in south Africa"*<br>*¬ The gap to be filled by this study should be clearly stated, such as the variables added other than other similar studies, method change or modification etc.*<br><br>*¬ The AOR better to precede the CI like AOR=1.68[1.12-2.53] with 95%CI.*	- We have changed the title of the manuscript to: "Child and adolescent food insecurity in South Africa: A household-level analysis of hunger"<br><br>- Please see lines 271-275 where we have adapted the language to address this point and clarify the research gap: "There is a paucity of nationally representative data to assess the granular detail about food insecurity across the country. The critical need to address hunger in childhood necessitates particular attention to the factors affecting food insecurity in households with children and adolescents—such as the household size, household head sociodemographics and dependency status of children—so that nutrition-sensitive interventions can be developed and/or strengthened"<br>- We have reconfigured the presentation of the AORs and 95% CIs to make it clearer throughout the text.

¬ *Instead of saying lower odds of experiencing hunger, I recommend to say there was a preventive/protective risk for hunger for those AOR<1*<br><br>¬ *Please minimize the key words*<br><br>¬ *The table titles don't contain time period*<br><br>¬ *In the discussion part the implication of the study should be stated after comparing and contrasting.*<br><br>¬ *The conclusion is better to declare that food insecurity (experiencing hunger), at risk of hunger and food secured were low, medium or high.*<br>*Regardless of these minor corrections the paper is very interesting and informative. It is well organized; better to be published. Thank you!!!*	- This is how we had previously expressed our results; however, earlier reviewers indicated that using the language of "preventive/protective risk for hunger" was not appropriate in this context. Therefore we changed the language according to their preferences.<br><br>- We removed three key words: "household", "hunger" and "CCHIP Index"<br><br>- The time period throughout the study is the same. We have stated this in the Methods and in all other references to the SANHANES-1 as the original data source<br><br>- We have included the implications of our study in the Conclusion section of the manuscript<br><br>- The main outcome indicator for our study was the degree of household food insecurity as measured by the Community Childhood Hunger Identification Project (CCHIP) Index. The CCHIP has been internationally validated according to a particular scale with three different outcome measures: 'food secure', 'at risk of hunger' and 'experiencing hunger'. These three categories are standard, so it would not possible to re-categorise into 'low', 'medium' or 'high' as suggested.
Journal Requirements<br><br>*Please review your reference list to ensure that it is complete and correct. If you have cited papers that have been retracted, please include the rationale for doing so in the manuscript text, or remove these references and replace them with relevant current references. Any changes to the reference list should be mentioned in the rebuttal letter that accompanies your revised manuscript. If you need to cite a retracted article, indicate the article's retracted status*	- We went through each reference to determine its retraction status. We do not cite any publication that has been retracted.

in the References list and also include a citation and full reference for the retraction notice.	
