# Supplementary material for: Child and adolescent food insecurity in South Africa: A household-level analysis of hunger
Source: PLoS One. 2022 Dec 28;17(12):e0278191. doi: 10.1371/journal.pone.0278191 (PMC9797094; doi:10.1371/journal.pone.0278191)
Supplement: Attachment — Submitted filename: Review Report PLoS.docx [file pone.0278191.s004.docx]

**Review Report**

**PONE-D-20-39991**

Dear editor, I am submitting the review report on Manuscript ID: PONE-D-20-39991 titled “**Childhood food insecurity in South Africa: a household level analysis of hunger**”. Since the previous research studies related to food insecurity lack a specific focus on households with children, so I found the study worth reading. A comparative analysis of different household characteristics between households with and without children is made. Various descriptive and inferential statistical methods have been used by the authors to analyze the data. The findings of the study are quite interesting and believable. The language and draft of the paper is also excellent.

In my view, this paper will be worth reading for the readers of PLoS ONE. I therefore recommend this paper for publication in PLoS ONE.
