# Supplementary material for: Child and adolescent food insecurity in South Africa: A household-level analysis of hunger
Source: PLoS One. 2022 Dec 28;17(12):e0278191. doi: 10.1371/journal.pone.0278191 (PMC9797094; doi:10.1371/journal.pone.0278191)
Supplement: Attachment — Submitted filename: Response to Reviewers_2021_06_24.docx [file pone.0278191.s005.docx]

Enquiries: Mr. Siluleko Mkhize

Contact number: +27 84 635 7216

Email: [843928@students.wits.ac.za](mailto:843928@students.wits.ac.za)

Date: 24 June 2021

Academic editor: Rubeena Zakar, PhD

PLOS ONE

1265 Battery Street
Floor 2
San Francisco, CA 94111
United States
Phone: +1 (415) 624-1200 | Fax: +1 (415) 546-4090

**Response to reviewers’ comments**

**Manuscript:** PONE-S-20-49924

**Title:** Childhood food insecurity in South Africa: A household-level analysis of hunger

| **Queries** | **Responses** |
| --- | --- |
| **Comments from academic editor Rubeena Zakar, PhD** |  |
| 1. Please ensure that your manuscript meets PLOS ONE's style requirements, including those for file naming. | Our manuscript now meets PLOS ONE’s style requirements. The following changes were made directly in the final manuscript:   1. Title headings are now in the correct font size 2. Figures were re-labelled in the correct format 3. Acknowledgement section was moved to the correct place 4. Vancouver referencing was updated and numbers placed in square brackets in the text 5. Where necessary, files were re-named |
| 2. Please include your tables as part of your main manuscript and remove the individual files. Please note that supplementary tables (should remain/ be uploaded) as separate "supporting information" files. | Tables were moved into the main body of the manuscript (Results section). S1 Fig, S1, S2 and S3 Tables and S1 Database are uploaded as separate “supporting information” files. |
| 3. We note that you have indicated that data from this study are available upon request. PLOS only allows data to be available upon request if there are legal or ethical restrictions on sharing data publicly. | As no legal or ethical restrictions apply, we have included the de-identified anonymised data set used for this study as a “supporting information” file entitled S1 Database. The data availability statement should now read:  *The final de-identified anonymised data set used in this study is provided as a “supporting information” file in this manuscript (S1 Database). A data sharing agreement was signed with the Human Sciences Research Council (HSRC), the curator of the data collected in the 2012 SANHANES survey. Adult SANHANES data are available through registered access from the HSRC data repository at:* [[*http://curation.hsrc.ac.za/Datasets-XKAHAA.phtml*](http://curation.hsrc.ac.za/Datasets-XKAHAA.phtml)](https://witscloud-my.sharepoint.com/personal/843928_students_wits_ac_za/Documents/Book.xlsx?web=1)*. Child data are under third party rights and can be accessed upon reasonable request by contacting* [*datahelp@hsrc.ac.za*](mailto:datahelp@hsrc.ac.za)  In addition, S1 Fig, Database Management, Organisation and Integration, has been revised with additional explanation about the comprehensive data management strategy that was employed to derive the final analytic sample and explanatory text added, to facilitate access to the data included for this study. |
| a) If there are ethical or legal restrictions on sharing a de-identified data set, please explain them in detail (e.g., data contain potentially identifying or sensitive patient information) and who has imposed them (e.g., an ethics committee). Please also provide contact information for a data access committee, ethics committee, or other institutional body to which data requests may be sent. b) If there are no restrictions, please upload the minimal anonymized data set necessary to replicate your study findings as either Supporting Information files or to a stable, public repository and provide us with the relevant URLs, DOIs, or accession numbers. Please see <http://www.bmj.com/content/340/bmj.c181.long> for guidelines on how to de-identify and prepare clinical data for publication. |  |
| 4. Thank you for stating the following in the Acknowledgments Section of your manuscript:   *[SM acknowledges the South African National Research Foundation for financial support of his postgraduate studies which enabled him to pursue this research.]*  We note that you have provided funding information that is not currently declared in your Funding Statement. However, funding information should not appear in the Acknowledgments section or other areas of your manuscript. We will only publish funding information present in the Funding Statement section of the online submission form.  Please remove any funding-related text from the manuscript and let us know how you would like to update your Funding Statement. Currently, your Funding Statement reads as follows:  *[The author(s) received no specific funding for this work]*  Please include your amended statements within your cover letter; we will change the online submission form on your behalf. | Funding information has been removed from the acknowledgements section of the manuscript.  Thank you for updating the online submission form to the amended Funding Statement below:  *SM received funding from the South African National Research Foundation (NRF): Freestanding, Innovation and Scarce Skills Development Fund Masters and Doctoral Scholarships (Reference; SFH170525233408 and MND190719457401) to support his postgraduate studies which enabled him to pursue this research; as well, supplemental funding was sourced from the Carnegie Corporation of New York.* |
| 5. Please include captions for your Supporting Information files at the end of your manuscript, and update any in-text citations to match accordingly. | Captions for all “Supporting Information” files have been added at the end of the manuscript (immediately after the References section). In-text citations were revised accordingly. |
| **Reviewers’ comments to author** |  |
| **Reviewer #1:**  Since the previous research studies related to food insecurity lack a specific focus on households with children, so I found the study worth reading. A comparative analysis of different household characteristics between households with and without children is made. Various descriptive and inferential statistical methods have been used by the authors to analyze the data. The findings of the study are quite interesting and believable. The language and draft of the paper is also excellent. | We appreciate the reviewer’s positive assessment of the manuscript, which require no changes to the manuscript. |
| **Reviewer #2:** |  |
| Generally, I found this manuscript to be well written. However, the relevance to current circumstances is questionable and the findings are not adequately explored.  I provide specific comments below if you choose to make revisions. | We appreciate the reviewer’s substantive critique of the manuscript and have addressed in detail each of the specific comments below. |
| 1. The SANHANES-1 dataset has been used for many publications in the academic literature. The introduction needs to better summarise previous research and identify the gap in the literature that this study seeks to fill.   Please reference Shisana and colleagues 2013 report at the very least. An early blog post by Parker in 2013 on the data may also be relevant. | We have modified the text to better summarise previous research, including the SANHANES-1, and clarify the research gap.  In the final manuscript, see the Introduction section, Lines 107 – 113; 126 – 134.  References were added, including Shisana et al. [11] The Reference list has been updated to reflect these additions. |
| 2. Lines 92 – 20 years of age seems like an arbitrary cut-off for the definition of a child. | We justify the age selection in the Methods section, Lines 190 – 193. |
| 3. Lines 96 & 419 refers to improvements between 2002 and 2017. Source and further information is needed. | Controversial evidence in the literature, and no use of comparable measurements (eg: the CCHIP Index), resulted in us removing these statements. |
| 4. Line 138 introduces the concept of nutrition-sensitive interventions without any definition, background, or references. Consider adding a paragraph summarising nutrition-sensitive intervention generally and in South Africa and how sociodemographic correlates can inform the design of these interventions to improve childhood health. | The comment has been addressed in Lines 146 – 153, and the notion of nutrition-sensitive interventions has been picked up in the Discussion as well. |
| 5. Lines 140-140 The sentence spanning these lines is repeating information from earlier in the introduction. Rephrase. | Sentence removed. |
| 6. Lines 198-199 The sentence spanning these lines is superfluous. This is rudimentary data manipulation and is expressed in the previous sentence. | Sentence removed. |
| 7. Lines 273-275 This is unclear. What were the differences? | We have re-worded the sentence for clarity and directionality (Lines 303 – 306).  *A further analysis of the collapsed locality categories (formal vs informal) yielded the following: 37.2% of households with children were informally located; and, of these, only 28.9% were food secure. In contrast, of the 62.9% of households with children located in formal areas, 53.6% were food secure (p<0.001).* |
| 8. Lines 287, 290, 305, 312 Identifying factors as being directly ‘protective for experiencing hunger’ is misleading. Many of these factors are likely causally related to asset endowment and/or income. They are not protective of but negatively related to. However, this term is appropriate when discussing social grants – that are designed to be protective against extreme poverty and food insecurity. | Sentences have been re-worded to “had lower odds of” instead of “protective for” experiencing hunger in the Results and Discussion sections. The language in the Abstract has also been revised.  The relationship to social grants, pensions and remittances retains the wording “protective against” experiencing hunger.  All of these changes are visible in the Revised Manuscript with track changes document. |
| 9. Lines 312-313 The sentence spanning these lines seems more emotive than the rest of the results section. | The sentence has been revised according to scientific language. |
| 10. A theme throughout the manuscript is this purported influence of having children on food insecurity. This appears twice in the introduction, is expressed as a poignant point in the results section and in discussion it is suggested that there is a mounting body of evidence on the topic. The evidence as presented in this manuscript is weak in supporting the assertion “children below age 18 years in households is independently associated with food insecurity, beyond sociodemographic characteristics and measures of socioeconomic status”. | We have addressed this point through the following changes to the manuscript:   1. We present new evidence in the Results section (Table 3 and text), corresponding to Lines 376 – 387, to strengthen the evidence for the presence of children in households and their impact on food insecurity.      2. This has been carried forward into the Discussion, Lines 402 – 405. |
| 11. The most interesting finding in this manuscript is that “social grants and pensions, or remittances, did not alleviate hunger or the risk of being food insecure.” This needs to be analysed and discussed further. | We addressed this comment as follows:   1. We amended Table 1 to include Source of Income, in order to compare households with and without children (Lines 287 – 293). 2. We did further analysis (Table 3) and added to the Discussion (Main source of household income and social grants). The new section now reads (Lines 445 – 449):   *“In fact, social grants were not protective in these households, with a 76% increase in being at risk of food insecurity and double the odds for being food insecure. Furthermore, even when controlling for the sociodemographic characteristics of the household head and the main source of income, the risk of experiencing hunger was increased with the presence of children in a household.”*  Together with Lines 461 – 465:  *“Furthermore, while they observed that the Older Persons Grant and remittances had substantial and favourable effects on the dietary diversity score, the Child Support Grant did not [26]. Our findings did not support a similar benefit for households without children. Moreover, when children were present, reliance on social grants, pensions or remittances as the main source of income, was not protective against food insecurity.”* |
| 12. The link between this manuscript and the COVID-19 pandemic is tenuous. The authors have done very little to link the dated dataset to present day. | The text has been updated in the Discussion to address COVID-19 considerations and food insecurity: Lines 488 – 498. |
| 13. Inconsistency in p value presentation. | We amended presentation of p values to three decimal points, and added p values to the Odds Ratios throughout the manuscript. |
| 14. Original sources need to be cited. | References have been amended to cite original sources. |

We submit these changes together with the revised manuscript and supplemental files.

Kindly note that I am an MSc student at Wits University; and, while I appreciate the title of “Dr”, it is a degree that I have not yet earned.

(Mr.) Siluleko A. Mkhize
