# Supplementary material for: Child and adolescent food insecurity in South Africa: A household-level analysis of hunger
Source: PLoS One. 2022 Dec 28;17(12):e0278191. doi: 10.1371/journal.pone.0278191 (PMC9797094; doi:10.1371/journal.pone.0278191)
Supplement: Attachment — Submitted filename: Response to Reviewers_06_Jan_2022.docx [file pone.0278191.s007.docx]

Enquiries: Prof Laurel Baldwin-Ragaven

Contact number: +27 791625228

Email: laurel.baldwin-ragaven@wits.ac.za

11 January 2021

Academic editor: Rubeena Zakar, PhD

PLOS ONE

1265 Battery Street
Floor 2
San Francisco, CA 94111
United States
Phone: +1 (415) 624-1200 | Fax: +1 (415) 546-4090

Dear Dr. Zakar,

**Manuscript:** PONE-D-20-39991R1

**Title:** Childhood food insecurity in South Africa: A household-level analysis of hunger

We appreciate the reviewers’ comments on the manuscript and welcome the opportunity to address the specific queries raised below. Kindly note that the line numbers referenced below correspond to the PDF version of the manuscript.

| **Reviewer comments** | **Authors’ responses** |
| --- | --- |
| **Reviewer #2** |  |
| 1. […] there are still substantial deficiencies in the interpretation of the literature and the interpretation of results. In general, I find that the language used in the manuscript suggests that causality has not been appropriately interpreted. In addition, my initial points still stand on: i) the purported influence of having children on food insecurity and, ii) social grants and pensions, or remittances. | We revisited the literature and the results. This study is a secondary data analysis with a cross-sectional design and therefore cannot assess causality. The model was built to determine associations between food security and the independent variables (the presence of children in a household; and social grants/pensions/remittances) adjusting for sociodemographic characteristics of the household head. Our analysis showed a statistically significant relationship between food insecurity and the presence of children of any age in a household. In addition, the source of income through social grants, pensions, or remittances showed persistent statistically significant higher odds of being at risk of hunger or experiencing hunger in the household **(Figures 2A and 2B)** |
| 2. The classification of children being under 0-19 is problematic. In the introduction, the authors emphasise the implications of household food insecurity among children, where the implications of undernutrition for wasting and stunting are most relevant for young children, less so for adolescence. Given the age threshold, this background information is irrelevant to the present study. Further, the cited studies do not justify the threshold used in this study. Revisit the analysis in order to i) better account for the gender of household head and child association and, ii) assess the sensitivity of your results to age - using thresholds more relevant to dependency on a carer. | Lines 107-119 in the Introduction have been edited to address the reviewer’s comments on malnutrition in children and adolescents.  Lines 205-206 of the Methods section clarifies the inclusion of children and adolescents up to age 19 years.  In order to better account for the relationship between the gender of household head and the presence of children, we conducted an analysis comparing the sociodemographic characteristics of male and female heads and added a new **Table 3.**  In order to assess the dependency threshold of children, we ran multinomial regression analysis between food security and sociodemographic characteristics of the household head and household location while adjusting for the presence of at least one child aged 15-19 years old (**Table 4**).  The Methods and Discussion sections of the manuscript have been amended (lines 222-226, 263-265, 361-367, 383-386, 388-399, 429-434, 445-448, 509-545) to reflect these additional  analyses.  NB: We removed Table 3 from the previous version of the manuscript and supplemental files: S2, S3 and S4 Tables to highlight the central findings of this study. |
| **Comments from Reviewer #3** |  |
| 1. L141-143: Any reason found in the literature for this. It will be nice if you add few reasons as being found in the literature. If there is none, it may be stated. | We reviewed the literature and amended the Introduction in lines 149-157. |
| 2. L202-204: Was the ethical clearance obtained for the use of the secondary data or this was the ethical clearance obtained for the collection of the primary data? If the latter holds, please re-phrase to indicate that. | Rephrased: see lines 215-218 |
| 3. L207: Household-level or Household level? Please check the entire manuscript and be consistent with one | We now use ‘household-level’ throughout the manuscript |
| 4. L227: Is it arithmetic or geometric mean? Please indicate? | This is an arithmetic mean, see lines 245 and 292 |
| 5. Please check for other comments that were inserted directly in the manuscript. | All comments that were inserted directly into the manuscript were addressed and visible in the track changes version of the manuscript. |

We submit these changes together with the revised manuscript in both PDF and track changes mode.

Kind regards,

**Prof Laurel Baldwin-Ragaven**
