# Supplementary material for: Child and adolescent food insecurity in South Africa: A household-level analysis of hunger
Source: PLoS One. 2022 Dec 28;17(12):e0278191. doi: 10.1371/journal.pone.0278191 (PMC9797094; doi:10.1371/journal.pone.0278191)
Supplement: Attachment — Submitted filename: RESPONSE TO REVIEWERS_2022_05_12.docx [file pone.0278191.s008.docx]

RESPONSE TO REVIEWERS – 2022/05/12 Revision required [PONE-D-20-39991R2] - [EMID:61e94e729c497602]

| Reviewer 2 | 1. Recommendation for publication: *No response* 2. Technically sound and data supporting conclusions: *Partly* 3. Review Comments to the Author: *Some of these revisions have, indeed, improved this manuscript - particularly in incorporating Table 3 and Table 4. Unfortunately, my underlying concerns remain.* | Reviewer 2 acknowledges improvement in the manuscript following the additional analyses and reporting of results in Tables 3 and 4. However, Reviewer 2 has persistent concerns. |
| --- | --- | --- |
|  |  |  |
| Reviewer 3 | *Please check L-187, a reference is missing, and please add the reference* | No reference is missing here. An appropriate reference was provided for the direct quote. Rather, we are indicating partially deleted text from a direct quotation. To avoid confusion and conform to the Chicago Style Manual, we have now changed this from brackets with three dots […] to an ellipsis with three dots ( . . . ) to signal that part of the text has not been included in the quotation. |
