# Supplementary material for: Child and adolescent food insecurity in South Africa: A household-level analysis of hunger
Source: PLoS One. 2022 Dec 28;17(12):e0278191. doi: 10.1371/journal.pone.0278191 (PMC9797094; doi:10.1371/journal.pone.0278191)
Supplement: Attachment — Submitted filename: Peter_Review_39991.docx [file pone.0278191.s010.docx]

**Reviewer’s Comments**

**Summary**

Thank you for the opportunity I have to provide a review for this important article on Childhood food insecurity in South Africa. The authors have done a good job of identifying the extent of childhood hunger and factors which contribute to it.

**Title**

- The title clearly depicts the research that was carried out with focus on the outcome, however the period or time of investigation is missing
- Each author’s contribution was clearly stated, and corresponding author’s contact is clear.

**Abstract**

- The abstract is unstructured, and it allowed for a good read. It is a good summary of the workdone.

**Introduction**

- The introduction revealed the intent of the study and raise curiosity as to what could be the finding of the study.
- However, the authors used very old references-I think they could use more recent references. I could not relate well with when the study weas conducted because I could not find anywhere it was mentioned. I could have missed it.
- The objectives were well stated: The authors wanted to determine the prevalence of food insecurity in households with and without children as well as to assess associations between the sociodemographic characteristics of the household head and degrees of hunger.

**Methods and Materials**

- The methods were well explained, and it is repeatable.
- The SANHANES-1 database was used for this study, however the question is when was the data extracted or when was this study conducted?
- Ethical consideration should have been the last in method. Authors can consider making the changes if they want.

**Results**

- Well written results and clearly stated
- In lines 354-6 – larger household as a predictor of experiencing hunger seems not to be related. LCL of 1.00 shows that if repeated a researcher can get AOR of 1.00. I think 4 digits LCL should be used if not it should not be reported because it is an unlikely association.

**Discussion**

- The discussion is well written and provides the necessary reasoning behind the results of the study
- It is was nice that they provided sub-headings which made an easy understanding of the discussion.
- The limitation of the study was clearly stated which eventually clarifies some of my earlier questions on when this study was conducted. But I think the authors should clearly state the year of study in the title.

**Conclusion**

- Provides answers to the objectives of the study
- The recommendations made by the authors may not be relevant since the authors agreed that this is a very old data, but the only data available for this kind of secondary data analysis.

**Recommendation**

- Accept with minor revision
